# Nurr1 Orchestrates Claustrum Development and Functionality

**DOI:** 10.1002/advs.202508999

**Published:** 2025-12-22

**Authors:** Kuo Yan, Andrew G. Newman, Pauline Lange, Susanne Müller, Marco Foddis, Stefan Paul Koch, Philipp Böhm‐Sturm, Maron Mantwill, Carsten Finke, Penghui Deng, Melissa Long, Dietmar Schmitz, Victor Tarabykin

**Affiliations:** ^1^ Institute of Cell Biology and Neurobiology, Charité‐Universitätsmedizin Berlin Corporate Member of Freie Universität Berlin and Humboldt‐Universität zu Berlin Berlin Germany; ^2^ Charité 3R – Replace | Reduce | Refine Charité – Universitätsmedizin Berlin Berlin Germany; ^3^ Department of Experimental Neurology and Center for Stroke Research Berlin Charité – Universitätsmedizin Berlin Berlin Germany; ^4^ Charité Core Facility Experimental MRIs, Charité‐Universitätsmedizin Berlin Corporate Member of Freie Universität Berlin and Humboldt‐Universität zu Berlin Berlin Germany; ^5^ Department of Neurology, Charité‐Universitätsmedizin Berlin Corporate Member of Freie Universität Berlin and Humboldt‐Universität zu Berlin Berlin Germany; ^6^ Berlin School of Mind and Brain Faculty of Philosophy Humboldt‐Universität Zu Berlin Berlin Germany; ^7^ Animal Behavior Phenotyping Facility, Charité‐Universitätsmedizin Berlin Corporate Member of Freie Universität Berlin and Humboldt‐Universität zu Berlin Berlin Germany; ^8^ Department of Genetics and Life Sciences Sirius University of Science and Technology Sirius Russia

**Keywords:** claustrum morphogenesis, claustrum dependent behaviors, functional connectivity, Nurr1 (Nr4a2), single cell transcriptome

## Abstract

Claustrum is the part of the forebrain known to be most widely interconnected tissue with almost all brain regions. It is believed to be involved in coordinating multiple cognitive behaviors including consciousness formation. However, little is known about the molecular mechanisms underlying its development and involvement in behavioral control. Here we show that Nurr1 (Nr4a2) is the key transcription factor orchestrating claustral morphogenesis, cell fate specification, connectivity and thus behaviors. Nurr1‐deficient claustral cells aberrantly migrate into insular cortex, shaping claustrum from a crescent‐like into a wing‐like structure. These cells ectopically turn on insular cortex genetic programs that were verified by single cell transcriptomics. Accordingly, functional connectivity of the claustrum is not properly formed and relevant behaviors are dysregulated in Nurr1‐deficient mice. Additionally, we show that Nurr1 regulates claustral neuron positioning and cell fate by suppressing Gαs‐PKA signaling.

## Graphical abstract

Nurr1 (Nr4a2) is the master transcription factor to control claustrum morphogenesis and cell fate decision postmitotically by inhibiting intracellular G‐protein signaling. Nurr1 deficiency alters the transcriptomic profiles of subcortical claustral neurons into neocortical insular neurons, resulting in defected claustrum development, impaired axonal wiring and functional connectivity, and thus disturbed claustrum dependent cognition.

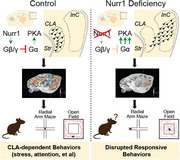

## 1 Introduction

The claustrum (CLA), a sheet‐like gray matter structure resident between the striatum and insular cortex (InC) in mice. It exists in most vertebrate species, ranging from birds, reptiles, rodents to human [1, 2, 3]. Little attention has been paid to this thin structure since it was identified 200 years ago until Francis Crick and Christof Koch proposed that CLA is the processing hub of sensory information from multiple cerebral sources to integrate coherent consciousness [4]. Though whether CLA is indeed the core hardware of consciousness is still uncertain to date, it is intensively interconnected to almost all global subareas [5, 6, 7], empowering its involvement in diverse cognitive activities, including but not limited to stress responses, attention, impulsiveness, salient sensation, sensorimotor cross‐modal selection, and sleep [2, 8, 9, 10, 11, 12, 13]. The insights into nerve circuitry underlying these physiological functions are accumulating due to recent advancement of optogenetics [8, 11, 14, 15]. However, very little has been elucidated about the molecular mechanisms governing CLA development and CLA‐dependent cognition yet.

Nurr1 (Nr4a2) is a transcription factor (TF) that contains a single zinc‐finger DNA‐binding domain. It is not only renowned for promoting dopaminergic neurogenesis as well as dopamine production in the substantia nigra [16, 17], but also the most frequently used marker for CLA glutamatergic neurons [1, 18, 19]. Nurr1 expression in CLA neurons is already detectable as early as E13.5 and lasts throughout life in mice (*Allen brain atlas*), but its role in CLA remains largely unknown. Here, we demonstrate experimental evidence that Nurr1's transcription activity is indispensable for orchestrating claustral morphogenesis, cell fate specification, axonal connectivity, and CLA‐regulated cognitive behaviors.

## 2 Results

### 2.1 Nurr1 Controls Claustrum Morphogenesis and Cell Fate Specification Postmitotically

**FIGURE 1:**
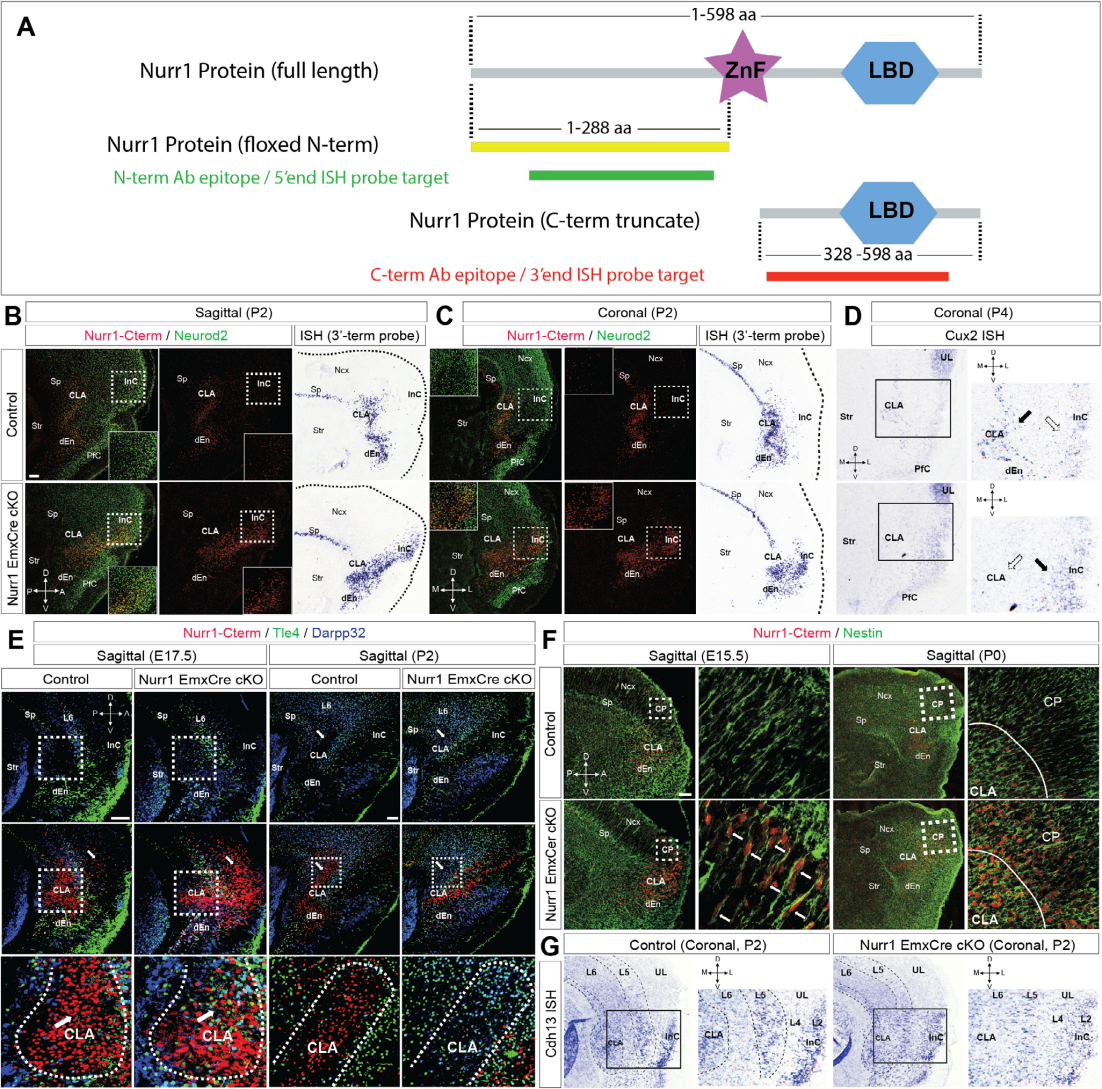
Nurr1 is indispensable for claustrum morphogenesis. (A) Illustration of Nurr1 molecular structure. The full‐length Nurr1 protein consists of a DNA‐binding zinc finger (ZnF) domain and a ligand‐binding domain (LBD). The coding sequence of N‐terminal region (Nterm, yellow, 1–288 amino acids (aa)) of Nurr1 protein is floxed. The truncated C‐terminal portion (Cterm, red, 328–598 aa) of Nurr1 protein in the same original open reading frame loses ZnF domain after Cre‐mediated recombination. The protein epitopes targeted by the antibodies (ab) or the transcript sequences targeted by the ISH probes are represented in green (Nterm ab or 5’end probe) and red (Cterm ab or 3’end probe), respectively. (B, C) Immunofluorescence (IF) for Nurr1‐Cterm and Neurod2 on sagittal and coronal sections of control and Emx1^Cre/Wt^ Nurr1‐deficient (cKO) brains at P2. Nurr1/Neurod2 double positive (Nurr1+/Neurod2+) claustral (CLA) neurons are mostly positioned between striatum (str) and insular cortex (InC), however, Nurr1‐deficient cells over‐migrate into InC. ISH using the Nurr1 3’end probe shows the same migration deficits of Nurr1‐deficient CLA cells as in IF. The framed images show magnification views of the dotted‐line boxed areas (the same in following figures). Sp, subplate; dEn, dorsal endopiriform nucleus; PfC, piriform cortex, Ncx, neocortex. D, dorsal; A, anterior; V, ventral; P, posterior, M, medial; L, lateral. Scale bar: 200 µm. (D) ISH using the Cux2 probe on coronal sections of control and Nurr1 cKO brains at P4. Cux2 is strongly expressed in a subpopulation of CLA neurons (solid arrowhead) and weakly expressed in InC (hollow arrowhead), but its expression was ectopically detected in the InC of Nurr1 cKO brains but no longer in CLA, implying that Cux2+ CLA neurons migrate into InC. UL, upper layers. (E) IF for Nurr1‐Cterm, Tle4 and Darpp32 on sagittal sections of control and cKO brains at E17.5 and P2. CLA neurons are already largely ceased in the area surrounded by Tle4+/Darpp32+ deeper layer (DL) neurons at E17.5, however, the majority of Nurr1‐deficient CLA cells already migrate across Tle4+/Darpp32+ DL neurons at E17.5 and are eventually located in InC anterior to DL neurons at P2. The original CLA area is filled with Tle4+/Darpp32+ DL neurons in Nurr1‐deficient brains. The arrowheads indicate CLA cells in control and cKO brains. The dotted lines delineate CLA territories from DL neurons. (F) IF for Nurr1‐Cterm and Nestin on sagittal sections of control and cKO brains at E15.5 and P0. Nurr1‐deficient CLA neurons always keep radial migration along Nestin+ radial glia. The lines in P0 images delineate the borders between CLA and Ncx. CP, cortical plate. (G) ISH using the Cdh13 probe on coronal sections of control and cKO brains at P2. The laminated expression of Cdh13 depicts the cortical cytoarchitecture by displaying its stronger expression in CLA, layer 5 (L5) and L2 of Ncx, but weaker expression in L6 and L4 along medio‐lateral axis. However, this cytoarchitecture is disrupted by migrating Nurr1‐deficient cells.

To characterize Nurr1's roles in forebrain development, we generated Nurr1 conditional “knock‐out” mice by crossing Nurr1^flox/flox^ with Emx1^Cre/Wt^ mice (Nurr1^flox/flox^;Emx1^Cre/Wt^; Nurr1‐deficient mice throughout text unless otherwise noted), where the floxed exon three of Nurr1 gene containing the start codon and DNA‐binding domain was deleted in dorsal telencephalon at ≈E10 by Cre‐mediated recombination [17, 20]. We reasoned that the design of Nurr1^flox/flox^ genetic targeting construct might enable a likelihood of tracing Nurr1‐expressing (Nurr1+) cells lacking its transcription activity, in that a C‐terminal (Cterm) truncated version of Nurr1 protein consistent with the original open reading frame under control of the same promoter might be still be present after recombination. To test this, we generated in situ hybridization (ISH) probes to target either Nurr1 5’‐ or 3’‐terminal transcripts (5’end or 3’end) (Figure 1A). While ISH using the 5’end probe verified Nurr1 expression depletion in mutant brains, Nurr1 3’end mRNA was still detectable in CLA, subplate (Sp), dorsal endopiriform nuclei (dEn), subiculum (Sb), and cortical plate (CP), similar to Nurr1 expression in controls (Figure S1A,B). Additionally, we procured antibodies to target either N‐terminal (Nterm) or Cterm portions of Nurr1 protein. Likewise, immunofluorescence (IF) using these antibodies confirmed the same results (Figure S1C). Notably, the truncated Nurr1‐Cterm polypeptide did not exhibit nuclear localization due to the removal of the nuclear localization signal peptide and DNA binding domain [21], indicating its inability to regulate transcription.

The general morphology of Nurr1‐deficient cortex was normal (Figure S2), consistent with previous report [16, 17]. Nurr1+ forebrain structures, such as Sp, Sb, and perisubicular complex, appeared also normal in morphology in Nurr1‐deficient brains as assessed by ISH for respective cell type markers—CTGF and S100a10 for Sp [22, 23] and Zbtb20 for hippocampus [24] (Figure S3A–D). Moreover, IF for Nurr1 and Zbtb20 depicted that the border, as separated cingulate cortex from hippocampal distal CA1, was comparable in control and Nurr1‐deficient brains (Figure S3E).

In contrast, Nurr1‐deficient CLA cells that still expressed Cterm truncated Nurr1 (Nurr1‐Cterm+) detached from Sp and invaded InC territory, as observed in both sagittal and coronal views in IF and ISH experiments (Figure 1B,C). To further verify the mislocalization of Nurr1‐deficient CLA cells, we performed ISH for Cux2, another CLA marker [19], and found Cux2+ CLA cells aberrantly present in InC of Nurr1‐deficient brains (Figure 1D). Consequently, CLA was reshaped from a crescent‐like into a wing‐like structure (Figure S4A). We next asked if the ectopic Nurr1+ neurons in InC of Nurr1‐deficient brains were present due to cell over‐production. We quantified the number of Nurr1‐Cterm+ cells in claustro‐insular region of Nurr1‐deficient brains, and found it similar to that in controls (Figure S4B). Furthermore, a few cleaved‐Caspase3+ cells were detected in the claustro‐insular regions of control and Nurr1‐deficient brains (Figure S4C,D). Nurr1 thus, is neither involved in the control of CLA neuron production nor survival.

We then questioned what happened to the original CLA territory when Nurr1‐deficient cells moved away. IF for Nurr1 and Tle4/Darpp32 delineated CLA cells from neighboring InC deeper layer (DL) neurons, depicting normal CLA cells are bilaterally restricted in an area between the stratum and Tle4+ DL neurons in control brains, consistent with another report [25]. However, Nurr1‐Cterm+ cells in Nurr1‐deficient brains had largely bypassed InC DL cells since E17.5 (Figure 1E). Intriguingly, Tle4/Darpp32‐positive DL cells, which were rarely detected in normal CLA area, became abundant in the original CLA territory of postnatal mutant brains (Figure 1E), an observation further strengthened by ISH for Fezf2 and Nfib (Figure S5). Nurr1+ neurons have been hypothesized to migrate either radially or tangentially to lateral, dorsal or ventral pallial destinations [1, 18], but solid experimental evidence still lacks. We next investigated the migration mode of Nurr1‐Cterm+ cells in Nurr1‐deficient brains, and found these cells constitutively migrated toward pia along Nestin+ radial glia throughout corticogenesis (Figure 1F), which disturbed the normal laminated organization of claustro‐insular complex (Figure 1G). Another key question is whether Nurr1 determines CLA cell positioning pre‐ or postmitotically. To answer this, we generated Nurr1^flox/flox^;Nex^Cre/Wt^ mice, where Nurr1 was selectively inactivated in cortical postmitotic compartment [26]. It turned out that Nex^Cre/Wt^ Nurr1‐deficient brains fully recapitulated the CLA morphological phenotype as in Emx1^Cre/Wt^ mutant brains (Figure S6A,B), suggesting Nurr1 acts in postmitotic neurons. Collectively, Nurr1 coordinates CLA cell positioning and interaction with surrounding tissues to shape the distinct morphology of CLA.

**FIGURE 2:**
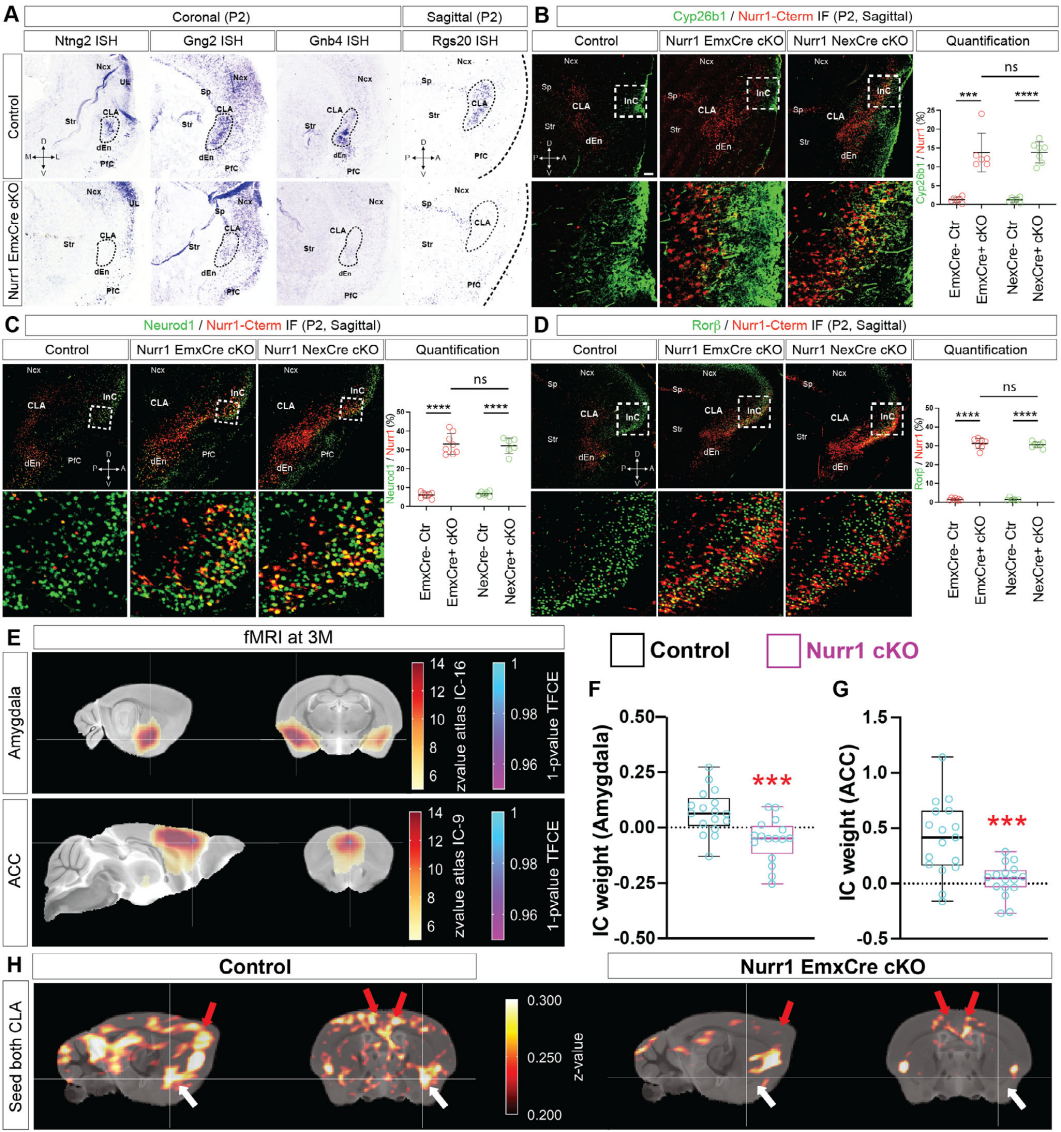
Nurr1 is a key regulator to control claustral cell fate and functional connectivity. (A) ISH using the Ntng2, Gng2, Gnb4 and Rgs20 probes on brain sections of control and Nurr1 cKO mice at P2. Ntng2 is expressed in CLA, UL and PfC in control brains but selectively downregulated in CLA of cKO brains. Gng2 expression is widely detectable in CLA, Sp, PfC and Ncx in control brains, while it is severely reduced in CLA but not in surrounding cells in cKO brains. Gnb4 is specifically expressed in CLA/dEn neurons in control brains but becomes rarely detectable in Nurr1 cKO brains. Rgs20 is normally expressed in Sp and CLA but selectively absent in CLA of cKO brains. The dotted‐line circles indicate CLA areas. (B–D) IF for Nurr1‐Cterm with Cyp26b1 (B), Neurod1 (C) and Rorβ (D) on sagittal sections of control, Emx1^Cre/Wt^ and Nex1^Cre/Wt^ Nurr1 cKO brains at P2. Nurr1+/Cyp26b1+ cells are rarely detectable in control brains but significantly increased in both Emx1^Cre/Wt^ and Nex1^Cre/Wt^ cKO brains. The proportion of Nurr1+/Cyp26b1+ cells in Emx1^Cre/Wt^ cKO brains (≈13.77%; *n* = 6) is increased by ≈9.70 ± 1.64 folds (*p* = 0.00010) relative to controls (≈1.29%; *n* = 6). The proportion in Nex1^Cre/Wt^ cKO brains (≈13.85%; *n* = 7) is increased by ≈9.95 ± 0.92 folds (*p* < 0.0001) relative to controls (≈1.27%; *n* = 6), to the similar level with Emx1^Cre/Wt^ cKO brains (*p* = 0.97, not significant, ns). The proportions of Nurr1+/Neurod1+ cells in Emx1^Cre/Wt^ and Nex1^Cre/Wt^ cKO brains are also elevated to similar levels (*p* = 0.72): the proportion in Emx1^Cre/Wt^ cKO brains (≈33.11%; *n* = 8) is increased by ≈4.49 ± 0.36 folds (*p* < 0.0001) relative to controls (≈6.03%; *n* = 7), and the proportion in Nex1^Cre/Wt^ cKO brains (≈32.09%; *n* = 6) is increased by ≈3.73 ± 0.26 folds (*p* < 0.0001) relative to controls (≈6.78%; *n* = 6). Likewise, the proportions of Nurr1+/Rorβ+ cells in Emx1^Cre/Wt^ and Nex1^Cre/Wt^ cKO brains are elevated to similar levels (*p* = 0.60): the proportion in Emx1^Cre/Wt^ cKO brains (≈31.23%; *n* = 7) is increased by ≈19.92 ± 0.72 folds (*p* < 0.0001) relative to controls (≈1.49%; *n* = 7), and the proportion in Nex1^Cre/Wt^ cKO brains (≈30.59%; *n* = 7) is increased by ≈19.02 ± 0.44 folds (*p* < 0.0001) relative to controls (≈1.53%; *n* = 6). The statistics for (B–D) was analyzed by two‐sided Student's *t*‐test. Scale bar: 200 µm. (E–G) Resting state fMRI (rs‐fMRI) was used to examine functional connectivity of 17 networks in control and Nurr1 cKO brains. (E) Blood‐oxygen‐level dependent (BOLD) signal intensities in amygdala and anterior cingulate cortex (ACC) networks (red z‐score overlaid on top of grayscale anatomical MRI template) of control brains were positively over the threshold values relative to those of Nurr1 cKO brains, suggesting these networks were impaired in Nurr1 cKO brains. Clusters of significant voxels shown in blue were reconstructed by voxel‐wise group statistics followed by threshold free cluster enhancement to correct for multiple comparisons (also see “*Methods and Materials*”). Mean values of independent component (IC) weight in the significant clusters were quantified. The average IC weight in the amygdala network cluster is ≈0.071 (*n* = 17) in control brains, but significantly reduced to ≈−0.056 (*p* = 0.00090; *n* = 16) in Nurr1 cKO brains (F). The value in the ACC network cluster in control brains is ≈0.41, but reduced to ≈0.030 (*p* = 0.00027) in Nurr1 cKO brains (G). The statistics for (F, G) was analyzed by two‐sided *Student's t‐test*. (H) Seed‐based connectivity mapping approach to analyze the specifically connected networks to CLA. The synchronous BOLD signals of amygdala (white arrowheads) and ACC (red arrowheads) networks in Nurr1 cKO brains are weakened in comparison to control brains when both hemispheric CLA ensembles were seeded as regions of interest.

We next tested if Nurr1 also played a role in CLA cell specification. To this end, we first performed ISH for several CLA signature genes, such as Ntng2, Gnb4, and Gng2 [19, 27], and found all these genes to be significantly downregulated in CLA of both Emx1^Cre/Wt^ (Figure 2A) and Nex^Cre/Wt^ (Figure S6C–E) mutant brains. Moreover, single‐cell transcriptomics revealed a large spectrum of misregulated genes in the claustro‐insular tissues of Nurr1‐deficient brains (see below Figure 4). Rgs20, a member of G‐protein signaling regulator (RGS) family, was selectively attenuated in CLA, but not in Sp (Figure 2A). These results indicate that the specific genetic profile of CLA neurons is altered due to Nurr1 deficiency. Secondly, we tested if Nurr1‐deficient CLA neurons activated expression of InC genes thereupon their ectopic localization. Cyp26b1 is a widely accepted InC marker [1, 28, 29]. We quantified the proportions of Nurr1/Cyp26b1 double‐positive (Nurr1+/Cyp26b1+) cells relative to total Nurr1+ cells in claustro‐insular region. While few Nurr1+/Cyp26b1+ cells were detected in controls, a considerably increased portion of Nurr1‐Cterm+ cells (by ≈9.70 ± 1.64 folds, *p* = 0.00010) in the InC of Nurr1‐deficient brains switched on Cyp26b1 expression (Figure 2B). By the same token, we quantified the proportions of Nurr1+/Neurod1+ and Nurr1+/Rorβ+ cells in claustro‐insular region as Neurod1 and Rorβ are also preferentially expressed in InC but hardly in CLA *(Allen brain atlas*). The proportions of Nurr1+/Neurod1+ and Nurr1+/Rorβ+ cells were increased by ≈4.49 ± 0.36 folds (*p* < 0.0001) and by ≈19.92 ± 0.72 folds (*p* < 0.0001) in Emx1^Cre/Wt^ mutant brains relative to those of control brains, respectively (Figure 2C,D). Furthermore, the expression levels of these genes in Nex^Cre/Wt^ mutant brains were elevated to the comparable levels as those in Emx1^Cre/Wt^ mutant brains (Figures 2B‐D). These results suggest that Nurr1 plays a fundamental role in CLA cell fate decision postmitotically.

### 2.2 Functional Connectivity Associated With Claustrum Is Disrupted in Nurr1‐deficient Mice

Axonal projection pattern is one of the major aspects of neuron subtype identity [30]. To test if the axonal pathfinding of Nurr1‐deficient CLA neurons is altered accordingly, we performed retrograde axonal tracing by placing DiI crystals into three target regions—primary motor cortex (PMC, telencephalic), hypothalamus (HT, secondary prosencephalic), and thalamus (diencephalic). PMC and HT have been reported to be connected intensively with InC, but very weakly with CLA [5, 31, 32, 33, 34]. While efferent neurons to PMC were rarely rooted in CLA in control brains, Nurr1‐deficient cell somas in InC frequently colocalized with DiI retrograde signals, supported by that the ratio of Nurr1‐Cterm+/DiI+ cells in Nurr1‐deficient brains was increased by ≈28.52 ± 4.49 folds (*p* = 0.00026) relative to that in controls (Figure S7A). Likewise, the ratio of Nurr1‐Cterm+/DiI+ cells in Nurr1‐deficient brains was approximately triple (*p* = 0.0023) of that in controls in the scenario of HT retrograde labeling (Figure S7B). Additionally, DiI+ signals were detected in a fraction (≈21.78%) of Nurr1+ CLA cells in control brains when the dye crystals were placed at thalamic nuclei. However, Nurr1+/DiI+ cells were seldom detectable when Nurr1‐deficient CLA neurons over‐migrate into InC (Figure S7C). These data indicate that Nurr1‐deficient CLA neurons acquire axonal projection patterns of InC neurons.

Next we studied functional connectivity (FC) of CLA in the Nurr1‐deficient adult mice using resting state functional magnetic resonance imaging (rs‐fMRI). We first analyzed the FC of 17 cerebral networks (see “*Methods and Materials*”), and found amygdala and anterior cingulate cortex (ACC) networks were the most severely affected by Nurr1 deficiency (Figure 2E). Their clusters of network activity (i.e. independent component weight) were significantly lower than those of control brains (Figure 2F,G). In order to find out if the compromised connectivity in these networks is specifically correlated to CLA, we employed the method of seed‐based differential mapping according to the *Allen brain atlas* [7]. When both hemispheric CLA were seeded as the regions of interest (ROI), strong synchronous activity was detected in amygdala and ACC networks in control brains, but was considerably weakened in respective networks in Nurr1‐deficient brains (Figure 2H). This indicates that the impaired FC of these networks in Nurr1‐deficient brains is due to CLA abnormality. Analysis based on single hemispheric CLA seed depicted similar results (sagittal view), and also revealed that CLA was correlated to the FC of contralateral amygdala and ACC networks, which were reduced in Nurr1‐deficient brains as well (coronal view) (Figure S8A). Collectively, Nurr1 is necessary for CLA neurons to properly attain axonal wiring and FC.

### 2.3 Nurr1 is Essential for Claustrum‐dependent Cognitive Behaviors

**FIGURE 3:**
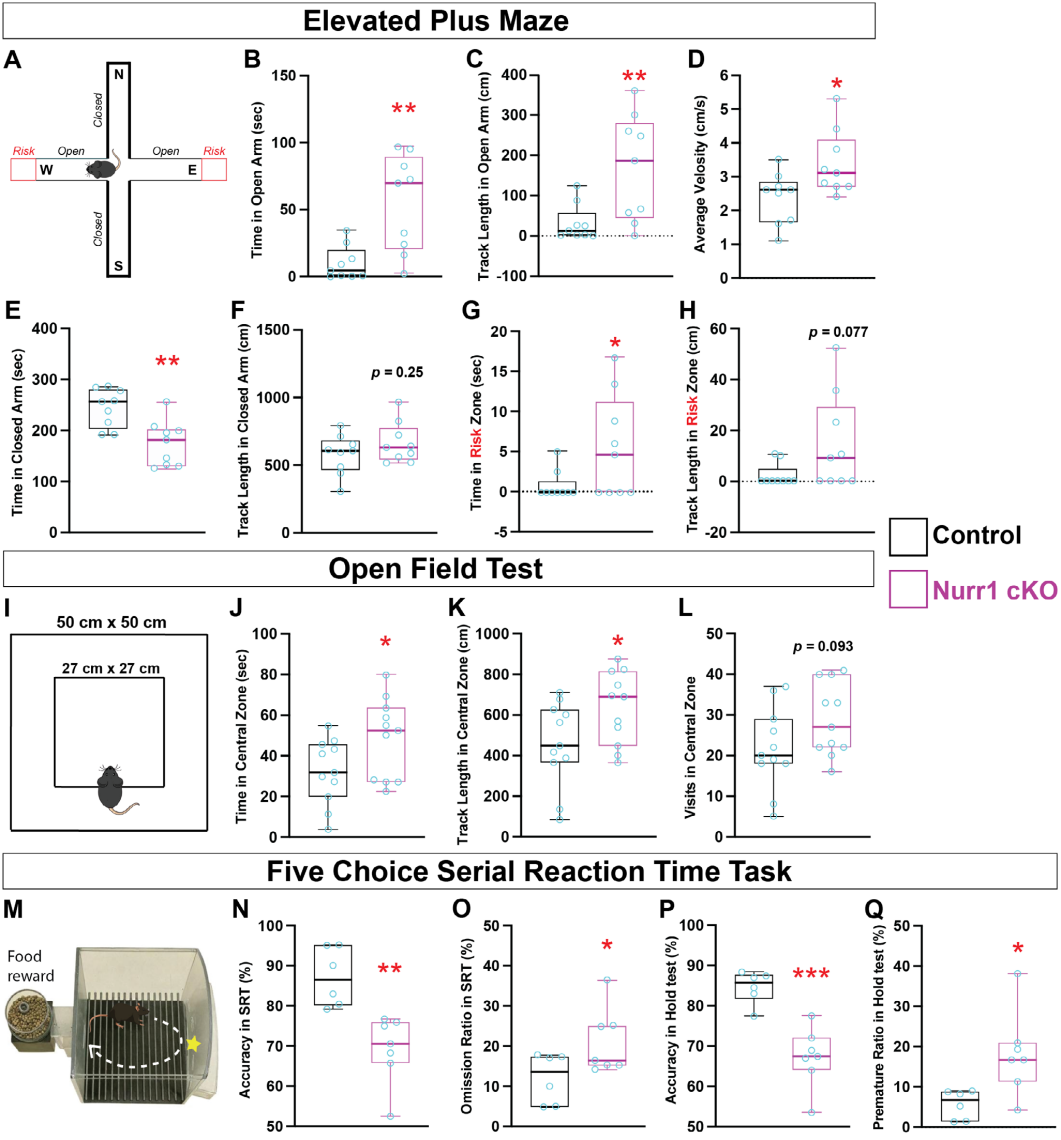
Claustrum associated behaviors are disturbed in Nurr1‐deficient mice. (A–H) Analysis of mouse behaviors in elevated plus maze (EPM). (A) The apparatus used in EPM (also see “Methods and Materials”). The north (N) and south (S) arms are closed with barriers and west (W) and east (E) arms are open arms. The far‐end ledge of open arms (10 cm) are defined as risk zones (red). (B, C) Nurr1 cKO mice spent more time (≈55.03 sec; increased by ≈4.41 ± 1.25 folds; *n* = 9; *p* = 0.0028) than control mice (≈10.18 sec; *n* = 9), and accordingly walked longer distance (≈167.9 cm; increased by ≈4.34 ± 1.47 folds; *p* = 0.0095) than control mice (≈31.42 cm) in open arms. (D) The average velocity of Nurr1 cKO mice (≈3.38 cm/s) in EPM was statistically higher than control mice (≈2.37 cm/s), increased by ≈42.71% ± 17.10% (*p* = 0.024). (E, F) Nurr1 cKO mice spent less time (≈175.3 sec; decreased by ≈28.41% ± 7.91%; *p* = 0.0024) than control mice (≈244.9 sec) in closed arms, but their track length (≈663.9 cm; *p* = 0.25) was similar to that of control mice (≈579.2 cm). (G, H) Nurr1 cKO mice travelled more time (≈5.56 sec; increased by ≈5.49 ± 2.58 folds; *p* = 0.049) than control mice (≈0.86 sec), and longer length (≈14.48 cm; increased by ≈5.36 ± 2.83 folds; *p* = 0.077) than control mice (≈2.28 cm) in risk zones. (I–L) Analysis of mouse behaviors in open field. (I) Schematic view of open field test apparatus. The side lengths of the open field square and central zone are 50 cm and 27 cm, respectively. (J) The average velocity of Nurr1 cKO mice (≈8.90 cm/s; *n* = 11) in the open field was significantly higher (*p* = 0.015) than control mice (≈7.31 cm/s; *n* = 11). (K, L) Nurr1 cKO mice spent more time in the central zone of open field (≈48.67 sec) than control mice (≈32.40 sec), increased by ≈50.22% ± 23.58% (*p* = 0.046). The travel distance of Nurr1 cKO mice in the central zone (≈633.4 cm) is longer than that of control mice (≈456.3 cm), increased by ≈38.81% ± 18.12% (*p* = 0.045). (M–Q) Analysis of mouse behaviors by five choice serial reaction time task (5CSRTT). (M) Schematic view of 5CSRTT apparatus. (N, O) The ratio of correct food pellet choices (accuracy) by Nurr1 cKO mice (≈69.27%; *n* = 7) is lower than that of control mice (≈87.18%; *n* = 6), decreased by ≈20.54% ± 5.06% (*p* = 0.0019) in the stimuli responsive tests (SRT) of 5CSRTT (also see “*Methods and Materials*”). The ratio of pellet omission in SRT of Nurr1‐deficient mice (≈20.98%) is higher than that of control mice (≈11.92%), increased by ≈76.05% ± 34.33% (*p* = 0.049). (P, Q) The accuracy of Nurr1 cKO mice in the hold test of 5CSRTT (≈67.28%) is also lower than that of control mice (≈84.71%), decreased by ≈20.58% ± 4.02% (*p* = 0.00033). The ratio of premature responses of Nurr1 cKO mice (≈18.17%) is significantly higher than that of control mice (≈5.64%), increased by ≈2.22 ± 0.80 folds (*p* = 0.018) in the hold test. The statistics for each behavior test parameter (A—Q) was analyzed by two‐sided *Student's t‐test*.

Neural circuitry between amygdala and CLA contributes to modulating responsive behaviors to stress in rodents [8]. Given the genetic disturbance of CLA and impaired amygdala‐CLA communication in Nurr1‐deficient mice, we reasoned if Nurr1 was involved in the regulation of stress‐induced emotional processing. We first tested control and Nurr1‐deficient mice using elevated plus maze (EPM). Nurr1‐deficient mice visited the open arms more frequently (Figure S8B), and exhibited significantly longer travelling time and distance at higher walking speed in the open arms of EPM than control mice (Figure 3A–D). Moreover, Nurr1‐deficient mice spent less time in closed arms than controls despite their similar moving distances (Figure 3E,F). These results implicate Nurr1‐deficient mice are less anxious to external stressors. We also defined the open arm ledges (10 cm) as high risk zones [35], and found that Nurr1‐deficient mice were more exploratory as they visited more often the risk zones (Figure S8C) and travelled longer time and distance in this area than control mice (Figure 3G,H), indicating that Nurr1‐deficient mice are more resistant to the fear of taking risk. We next assessed animals’ anxiety induced by a new environment using the open field. Nurr1‐deficient mice exhibited greater locomotion in the open field, and travelled longer time and distance in the central zone of the open field than control mice (Figure 3I–L). These data indicate that genetic abolishment of CLA ensemble by Nurr1 deficiency makes mice more resistant to stress‐elicited behavioral responses.

CLA is known to promote volitional attention via top‐down innervation from ACC [10]. To study if the impaired synchronous activity between CLA and ACC in Nurr1‐deficient mice causes attentional deficits, we performed five choice serial time task (5CSRTT). All mice were trained to procure food rewards only when they made a correct choice responding to the light stimulus in 5CSRTT (Figure 3M). Nurr1‐deficient mice exhibited decreased accuracy (Figure 3N; Figure S8D) but increased omission (Figure 3O and Figure S8E) than control mice to the rewards guided by light stimuli, indicating their suboptimal attentional performances. Impulsiveness was reported to be regulated by synaptic communication between CLA and ACC or prefrontal cortex [12, 14]. We then tested the mouse ability to withhold their impetuous choices during inter‐trial interval time till a visual stimulus in 5CSRTT, and found the premature responses of Nurr1‐deficient mice were increased at the expense of their accuracy compared with control mice (Figure 3P,Q and Figure S8F,G), suggesting Nurr1‐deficient mice are less capable of controlling impulsivity. Collectively, our data indicate that Nurr1+ neurons in the CLA play a critical role in optimizing such aspects of cognition as anxiety, fear, attention, and impulsiveness.

### 2.4 Claustro‐Insular Single Cell Transcriptomic Analysis Reinforces the Molecular and Functional Characterization

**FIGURE 4:**
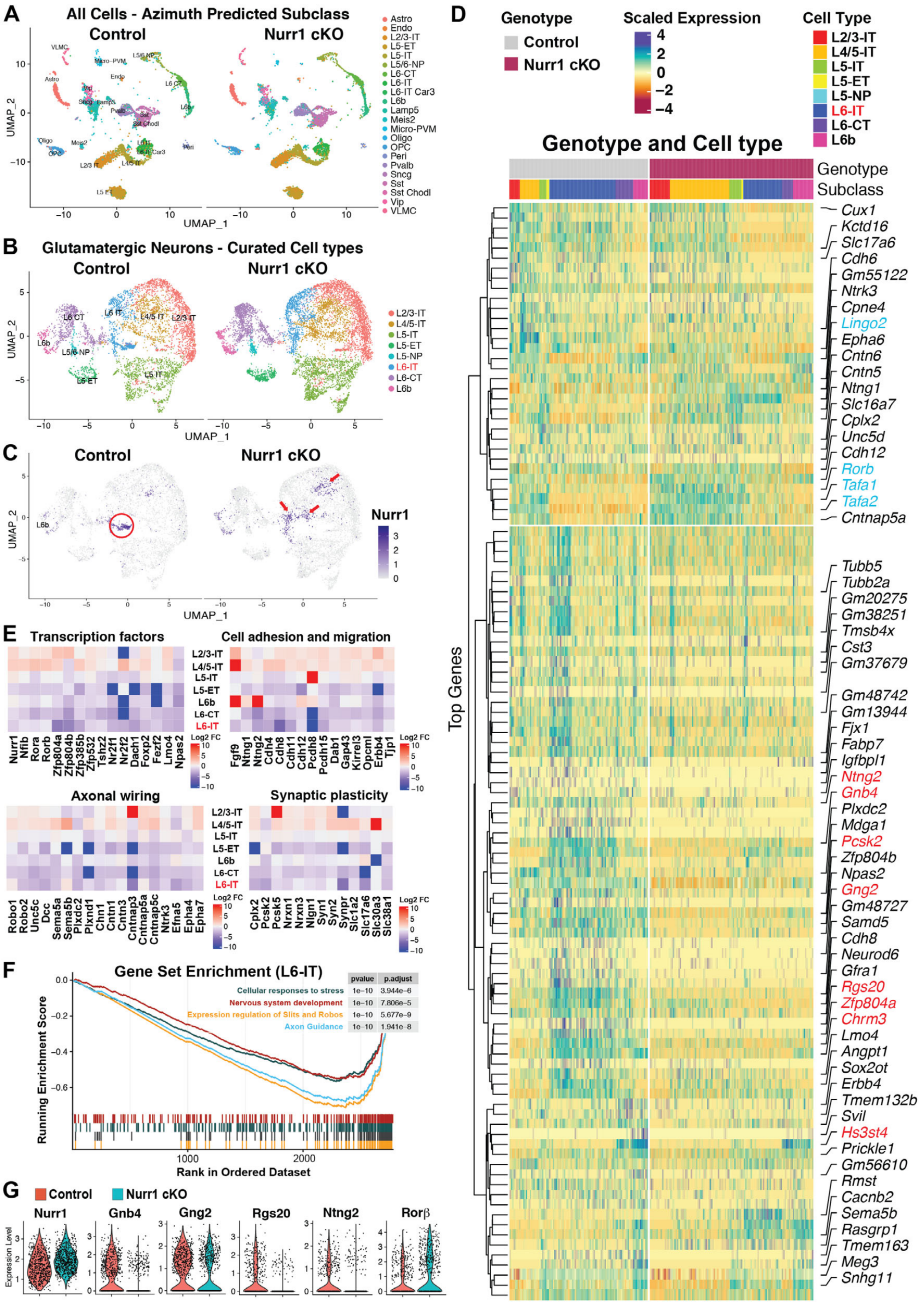
Transcriptomic profile of claustro‐insular tissue is misregulated in Nurr1‐deficient brains. (A) Uniform manifold approximation and projection (UMAP) of single cell transcriptomes based on all cells isolated from claustro‐insular tissues in control and Nurr1 cKO brains. Each point denotes an individual cell. Cell subtypes in UMAP are grouped in clusters and marked by colors. Astro, astrocytes; Endo, endothelial cells; L2/3‐IT, layer 2 and 3 intra‐telencephalon projecting glutamatergic neurons; L5‐ET and ‐IT, layer 5 extra‐ and intra‐telencephalon projecting glutamatergic neurons; L5/6‐NP, layer 5/6 near projecting glutamatergic neurons; L6‐CT and ‐IT, layer 6 corticothalamic and intra‐telencephalon projecting glutamatergic neurons; L6‐IT Car3, Car3+ subset of L6‐IT neurons; L6b, layer 6b neurons; Micro‐PVM, microglia or perivascular macrophage; Oligo, oligodendrocyte; OPC, oligodendrocyte precursor cells; Peri, pericytes; VLMC, vascular lepotomeningeal cell; GABAergic neuron clusters: Lamp5, Meis2, Pvalb, Sncg, Sst, Sst Chodl, Vip. (B, C) UMAP analysis of glutamatergic neurons populations in claustro‐insular tissues of control and Nurr1 cKO brains (B) and distribution of Nurr1+ cell in glutamatergic neuron UMAP (C). Nurr1+ cell subtypes are mainly distributed in L6b and L6‐IT clusters in controls, but their distribution is significantly altered in Nurr1 cKO brains. Nurr1+ CLA cells are mainly distributed in a subset of L6‐IT cluster in controls (red circle), but these cells in Nurr1 cKO brains are distributed in the L4/5‐IT (layer 4/5 intra‐telencephalon projecting glutamatergic neurons) cluster and other subpopulations of L6‐IT cells (red arrowheads). The expression level is marked from light blue (minimal) to dark blue (maximal). (D) Heatmap of top differentially expressed genes (DEGs) due to loss of Nurr1 across glutamatergic neuron subtypes: control (left panel) and Nurr1 cKO brains (right panel). Cell columns are grouped by cell subtype with unsupervised clustering of genes in rows. The DEG selection criteria are in “*Methods and Materials*”. The genes verified by ISH (Figure 2A and figure S11) are marked (upregulated in blue and downregulated in red). (E) Genes of interest with substantially altered expression in Nurr1 cKO brains were grouped with regard to their neural functions in cell type‐specific heatmaps (analyzed based on Nurr1+ glutamatergic neurons). Log2 fold changes (Log_2_‐FC) in expression levels of Nurr1 cKO brains relative to those of control brains are marked by color bars in the maps. (F) Gene set enrichment analysis for selected significantly enriched reactome pathways in L6‐IT cluster. The upper line plot is running enrichment score for each gene set. The lower plot represents the positions of genes from each respective gene set in the rank‐ordered dataset. (G) Violin plots for selected genes in the Nurr1+ glutamatergic neurons of claustro‐insular tissues. The expression level of Nurr1 transcripts in control brains is equivalent to Nurr1‐Cterm transcripts in Nurr1 cKO brains. The expression levels of Gnb4, Gng2, Rgs20 and Ntng2 are downregulated, whereas Rorβ expression is upregulated in Nurr1‐Cterm+ cells of Nurr1 cKO brains.

We next aimed to identify downstream effectors of Nurr1 that control CLA neuron activities. We isolated the anterior claustro‐insular portions of control and Nurr1‐deficient cortices at P0 and immediately disassociated cells for single nucleus transcriptome analysis. We recovered 15239 nuclei isolated from 15 control cortices and 19814 nuclei from 18 Nurr1‐deficient cortices. After projecting to a cell type annotation using Azimuth (mouse cortex reference) [36], we first identified three main cell populations: glutamatergic neurons, GABAergic neurons, and non‐neuronal cells in our tissues (Figure S9A) and generated a detailed landscape of claustro‐insular cell heterogeneity (Figure 4A). In order to observe the cell type‐specific changes in gene expression due to Nurr1 deficiency, we next performed Uniform manifold approximation and projection (UMAP) analysis for glutamatergic neuron population (Figure 4B) [37]. Nurr1+ cells were mainly detected in L6b (Sp) and L6‐IT (layer 6 intra‐telencephalon projecting glutamatergic neurons) clusters in control brains (Figure 4C), same as Nurr1 ISH and IF. The presence of truncated Nurr1 mRNA (Nurr1‐Cterm) in mutant brains enabled us to trace the distribution of Nurr1 lineage cells in Nurr1‐deficient brains. Interestingly, we found that a cell subset in L6‐IT cluster was missing in Nurr1‐deficient brains (Figure S9B), and that Nurr1 expression alteration was particularly notable in these cells (Figure 4C). Gnb4+/Car3+ neurons have been reported to be the major population of Nurr1+ CLA cells [36, 38]. UMAP analysis for Gnb4 and Car3 indicated that they were both strongly expressed in this subpopulation in control brains, which disappeared in Nurr1‐deficient brains (Figure S9C,D). These results together suggest that the missing population inside L6‐IT cluster in Nurr1‐deficient brains largely consists of CLA (and dEn) neurons. This conclusion is further corroborated by UMAP analysis for multiple CLA‐expressing genes (Figure S10A–F). Remarkably, there is a significant increase of Nurr1‐Cterm+ cells in L4/5‐IT (layer 4/5 intra‐telencephalon projecting neurons) as well as in Nurr1‐negative L6‐IT subpopulations in Nurr1‐deficient brains (Figure 4C). We then subset to Nurr1+ cells by evidence of at least one Nurr1 transcript in glutamatergic neurons, resulting in 541 nuclei in control and 639 nuclei in mutant tissues. In Nurr1‐deficient brains, the proportions of Nurr1 lineage cells in L4/5‐IT glutamatergic neurons were considerably elevated at the expense of L6‐IT cluster (Table S1). Our transcriptomic data are thus consistent with the above finding that CLA cells change their identity into neocortical neuron subtypes when Nurr1 is absent.

We next generated a heatmap with the top differentially expressed genes in Nurr1‐deficient brains (Figure 4D). ISH for CLA‐enriched genes testified to the cell type specificity of the map (Figure 2A and Figure S11). To better understand the biological effects of Nurr1, we grouped many of these genes by neural functionality and calculated the Log_2_ fold changes in a cell type‐specific manner. Specific TF expression is one of the principal properties in cell identity [30]. The Nurr1‐Cterm+ cells in claustro‐insular region of Nurr1‐deficient brains ectopically upregulated TFs normally expressed in InC neurons, such as Rorβ, Foxp2, and Nfib (Figure 4E), again strengthening that these neurons have turned into InC‐like cells. Consistent with the impaired CLA morphogenesis and FC in Nurr1‐deficient brains, several genes involved in cell migration, axon wiring, and synaptic plasticity were found misregulated in the Nurr1‐Cterm+ mutant neurons, particularly in the L6‐IT cluster (Figure 4E). We next performed gene set enrichment analysis (GSEA) for Nurr1 lineage cells in the L6‐IT cluster. GESA plot exhibited that the mRNA fractions in several reactome gene sets, such as nervous system development, axon guidance, and responses to stresses, were significantly downregulated (Figure 4F). This provides molecular interpretations for FC impairment and stress resistance in Nurr1‐deficient mice. Additionally, we could verify the expression alteration of those genes highlighted in Nurr1‐deficient CLA cells by violin and UMAP plots (Figure 4G and Figure S10). The transcriptomic data thus concretely support our molecular, FC, and behavioral characterization associated with CLA in Nurr1‐deficient brains.

### 2.5 Claustral Cell Positioning and Cell Fate Specification Relies on Suppression of Gαs‐PKA Signaling by Nurr1

**FIGURE 5:**
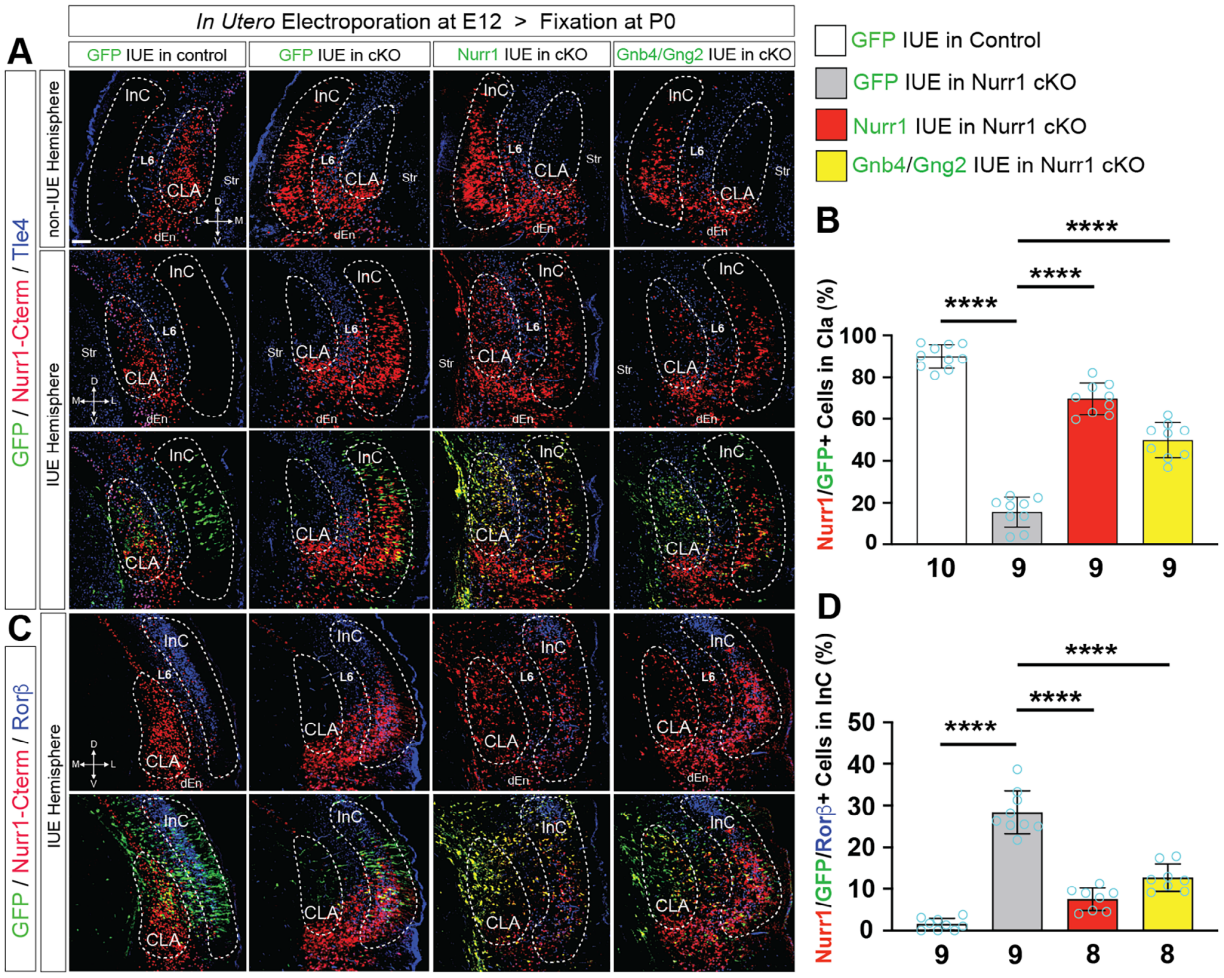
Nurr1 regulates claustral neuron positioning and cell fate by regulating G‐protein signaling. (A, B) IF for Nurr1‐Cterm and Tle4 on the coronal sections of non‐electroporated (non‐IUE) and electroporated hemispheres of control and cKO brains at P0 (A) and quantification for the proportions of Nurr1‐Cterm+/GFP+ cells in CLA relative to all double positive cells in various electroporation conditions (B). Non‐electroporated hemispheres serve as internal references to the electroporated ones of the same brains. GFP IUE into control embryos at E12.5 demonstrated almost all double positive cells resided in CLA (≈89.94%; *n* = 10), but GFP IUE into Nurr1 cKO embryos reduced the proportion to ≈15.44% (*n* = 9), depicting Nurr1‐deficient CLA cells largely migrate across Tle4+ cells into InC. IUE of bicistronic Nurr1‐GFP into mutant embryos restricted the Nurr1‐Cterm+ cells in the CLA area, elevating the proportion by ≈3.51 ± 0.22 folds (≈69.63% ± 3.47%; *n* = 9; *p* < 0.0001) relative to that of GFP IUE into mutant embryos. Moreover, Gnb4/Gng2 co‐electroporation into mutant embryos restricted a subpopulation of Nurr1‐Cterm+ cells in the CLA area, elevating the proportion by ≈2.23 ± 0.24 folds (≈49.89% ± 3.70%; *n* = 9; *p* < 0.0001). Notably, the Nurr1‐Cterm+ cells in the Nurr1‐ or Gnb4/Gng2‐electroporated mutant hemispheres were visibly less in InC areas than the non‐electroporated contralateral hemispheres. The statistical data of GFP‐electroporated control and Nurr1 cKO brains, or of GFP‐ and Nurr1‐(Gnb4/Gng2‐) electroporated Nurr1 cKO brains (B), were analyzed by two‐sided Student's *t*‐test (also in D). Scale bar: 100 µm. (C, D) IF for Nurr1‐Cterm, GFP and Rorβ on coronal brain sections (C) and quantification for the proportions of Nurr1‐Cterm/GFP/Rorβ triple positive cells relative to all Nurr1‐Cterm+/GFP+ cells (D). Nurr1 rarely colocalized with Rorβ in GFP‐electroporated control brains (≈1.54%, *n* = 9), but the proportion was increased to ≈28.37% ± 1.80% in cKO brains (*n* = 9; *p* < 0.0001). Nurr1 restoration by IUE into cKO brains lowered the proportion to ≈7.53% ± 2.05% relative to GFP‐electroporated cKO brains (*n* = 8; *p* < 0.0001). IUE of Gnb4/Gng2 into cKO brains significantly decreased the proportion to ≈12.71% ± 2.15% (*n* = 8; *p* < 0.0001).

Several top‐rated regulated genes by Nurr1 in our transcriptomic analysis were found to be involved in the control of G‐protein signaling, such as Gnb4/2 and RGS family members (Figures 2A and 4G; Figure S10). Additionally, Gng2 (Gγ2) can heterodimerize with Gnb4/2 (Gβ4/2) to stabilize the silent Gα effectors [39, 40]. We thus hypothesized that low activity of Gα subunits might be vital for CLA development. To test this, we performed “genetic rescue” experiments by in utero electroporation (IUE) of target genes in Nurr1‐deficient CLA cells at E12.5, the stage when birth peak of CLA neurons is in mice [41]. We first electroporated GFP into control and Nurr1‐deficient embryos as references and quantified the proportions of Nurr1+/GFP+ cells positioned in CLA area relative to the total double‐positive cells. While Nurr1+/GFP+ cells populated mainly in the CLA of GFP‐electroporated control brains (≈89.94%), only a small fraction of Nurr1‐Cterm+/GFP+ cells were still present in the CLA of GFP‐electroporated mutant brains (≈15.44%). Instead, the majority of Nurr1‐Cterm+/GFP+ cells were detected in InC area, consistent with their phenotype in the non‐electroporated hemispheres (Figure 5A,B). We then analyzed Nurr1‐GFP electroporated mutant brains, and found that Nurr1 restoration in mutant CLA cells prevented most of Nurr1+/GFP+ cells from migrating to InC (≈69.63% in CLA). The fraction should be even larger given some non‐CLA cells carrying enforced Nurr1 expression were counted into denominator. Notably, co‐electroporation of Gnb4‐ and Gng2‐GFP into Nurr1‐deficient CLA cells increased the proportion of Nurr1‐Cterm+/GFP+ cells in CLA by ≈2.23 ± 0.24 folds (*p* < 0.0001) relative to GFP‐electroporated mutant brains (Figure 5A,B). Restoration of Gα inactivators in Nurr1‐deficient CLA cells thus facilitates their positioning inside the CLA territory. We next asked if inhibition of Gα effectors is also involved in CLA cell specification. To this end, we performed IF for Nurr1‐Cterm, GFP, and Rorβ in the electroporated brains, and quantified the proportions of Nurr1‐Cterm+/GFP+/Rorβ+ triple positive cells relative to the total Nurr1‐Cterm+/GFP+ cells. This ratio was very low (≈1.54%) in the GFP‐electroporated control brains as Rorβ was rarely expressed in the normal Nurr1+ CLA neurons (Figures 2D and 5C), but it was increased up to ≈28.37% ± 1.80% in the GFP‐electroporated mutant brains (Figure 5D). Remarkably, IUE of Nurr1 and co‐IUE of Gnb4/Gng2 into the mutant brains decreased the ratios to ≈7.53% ± 2.05% and ≈12.71% ± 2.15%, respectively (Figures 5C,D). These results indicate that these Nurr1‐deficient CLA neurons, which have been retained at CLA by Gα inhibition, no longer express InC specific genes.

To test whether these Nurr1‐deficient neurons that are resident at CLA as a result of Gnb4/Gng2 restoration regain the connectivity of CLA neurons, we performed DiI retrograde tracing on the electroporated brains. When we placed the DiI dye in the hypothalamic nuclei, the ratio of Nurr1‐Cterm+/GFP+/DiI+ triple positive cells in the InC relative to all DiI+ cells in the GFP‐electroporated control brains was merely ≈1.30% due to absence of Nurr1+ cells in the InC, but this ratio in the GFP‐electroporated mutant brains was as high as ≈11.76% (Figure S12A). These results are in line with the previous observations in Figure S7B. Notably, the ratio in the Gnb4/Gng2‐electroporated mutant brains was reduced to ≈4.81% (Figure S12A). On the other hand, we also seeded DiI+ crystals at thalamus and quantified the ratios of Nurr1‐Cterm+/GFP+/DiI+ positive cells in the CLA relative to all DiI+ cells. DiI+ signals were often detectable in the Nurr1+/GFP+ CLA neurons of GFP‐electroporated control brains (≈11.51%), but rarely in the GFP‐electroporated mutant brains (≈0.61%) (Figure S12B), similar to the tendency in Figure S7C. Nevertheless, IUE of Gnb4/Gng2 in the mutant brains elevated the ratio up to ≈8.82% (Figure S12B), suggesting that a subset of Nurr1‐deficient CLA neurons retrieve their axonal projection to thalamus. These data imply that the Nurr1‐deficient neurons over‐expressing Gnb4/Gng2 regain the axonal projection trajectories of normal CLA neurons.

**FIGURE 6:**
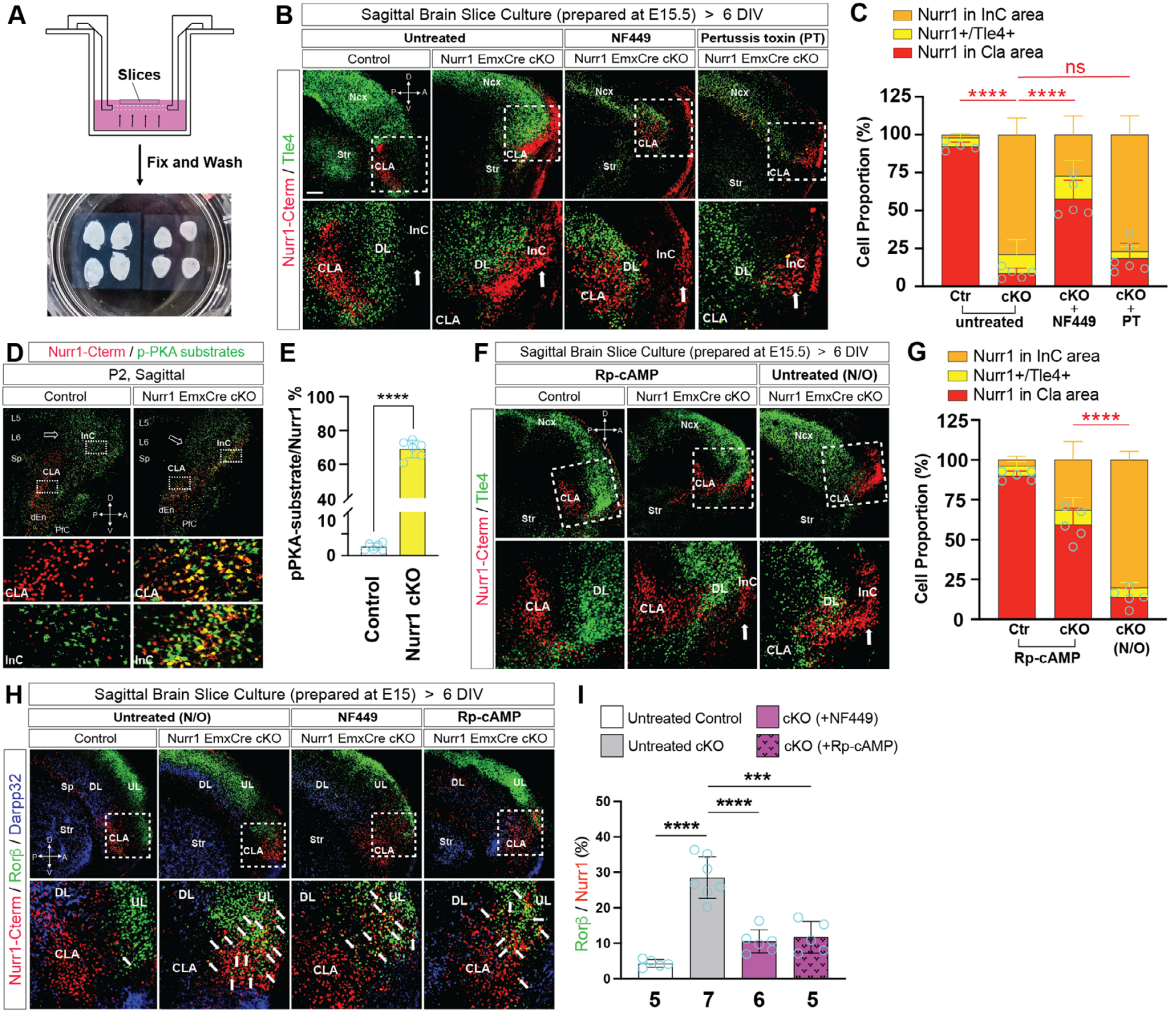
Low activity of PKA signaling is essential for claustral morphogenesis. (A‐C) Schematic view of organotypic brain slice culture (A). IF for Nurr1‐Cterm and Tle4 on sagittal brain slices of control and cKO brains (B) and quantification for the proportions of Nurr1‐Cterm+ cells in CLA (red bars) relative to all Nurr1‐expressing cells (C). The brain slices were prepared at E15.5 and cultured for 6 days in vitro. Most Nurr1+ CLA neurons (≈92.29%; *n* = 4) were properly positioned in control brains, but only a small fraction of Nurr1‐Cterm+ cells (≈8.78%; *n* = 5) were detected posterior to Tle4+ DL neurons in Nurr1 cKO slices. The proportion of Nurr1‐Cterm+ cells in CLA in cKO brains was increased by ≈5.58 ± 0.65 folds when NF449 (10 µm) was applied (≈57.74% ± 5.72%; *n* = 5; *p* < 0.0001), but not significantly altered when pertussis toxin (PT, 100 ng/mL) was applied (≈18.65% ± 4.62%; *n* = 6; *p* = 0.062), relative to that of untreated mutant brains. White arrowheads in (E) and (I) indicate the remaining ectopic Nurr1‐Cterm+ cells in InC. The statistical data of untreated control and Nurr1 cKO brains, or of untreated and NF449‐ (or PT‐) treated Nurr1 cKO brains (C), were analyzed by two‐sided *Student's t‐test* (also in G and I). Scale bar: 200 µm. (D, E) IF for Nurr1‐Cterm and phosphorylated PKA (p‐PKA) substrates at P2 and quantification for the proportions of double positive cells relative to all Nurr1‐expressing cells. The Nurr1‐Cterm+ cells in Nurr1 cKO brains exhibited tremendously elevated levels of p‐PKA substrates (≈69.07% ± 2.14%; *n* = 6) compared with that of control brains (≈2.06%; *n* = 6). The statistics in (E) was analyzed by two‐sided *Student's t‐test*. (F, G) IF for Nurr1‐Cterm and Tle4 on sagittal brain slices and quantification for the proportions of Nurr1‐expressing cells in CLA. Rp‐cAMP (100 µm) treated Nurr1+ cells in control brains were mostly located in CLA (≈90.05%; *n* = 5), similar to that of untreated control brains (≈92.29%). The proportion of Nurr1‐Cterm+ cells in the CLA of Rp‐cAMP treated mutant hemispheres was elevated to ≈59.30% ± 5.28% (*n* = 5; *p* < 0.0001) in comparison to that of untreated mutant hemispheres (≈13.96%; *n* = 5). (H, I) IF for Nurr1‐Cterm, Rorβ and Tle4 on sagittal brain slices (H) and quantification for the proportions of Nurr1+/Rorβ+ cells relative to all Nurr1‐expressing cells (I). The proportion in untreated Nurr1 cKO slices was up to ≈28.54% ± 2.69% (*n* = 7) as compared with that in untreated control brain slices (≈4.33%; *n* = 5). Treatment of cKO brain slices with NF449 and Rp‐cAMP reduced the proportions to ≈10.55% ± 2.70% (*n* = 6; *p* < 0.0001) and ≈11.81% ± 3.11% (*n* = 5; *p* = 0.00030), respectively, relative to that of untreated cKO slices.

Gα effector proteins are divided into several subclasses: Gαs, Gαi, Gαq and Gα_12/13_. Among them Gαs and Gαi cascades are known to be primarily involved in the regulation of cell motility [42]. In order to further investigate the effect of Gα downstream signaling on CLA cell behaviors, we combined brain slice culture with acute pharmacological treatment. The brain slices were prepared from E15.5 embryos, cultured for six days, and then fixed for IF analysis. The resulting tissues appeared morphologically intact (Figure 6A). Nurr1+ CLA cells in untreated control slices dwelled normally between striatal and Tle4+ InC DL neurons, but the majority of Nurr1‐Cterm+ cells in untreated mutant slices migrated across InC DL neurons toward the pia, similar to Nurr1‐deficient CLA cells in vivo (Figure 6B). We then quantified the proportions of Nurr1+ or Nurr1‐Cterm+ cells positioned in CLA relative to the total Nurr1 lineage cells. There was a huge fraction (≈92.29%) of Nurr1+ cells in the CLA of untreated control slices, but merely ≈8.78% Nurr1‐Cterm+ cells in the CLA of untreated mutant slices (Figure 6C). We next treated Nurr1‐deficient slices with NF449, a Gαs‐specific cell‐permeable inhibitor [43, 44], and found a larger fraction of Nurr1‐Cterm+ cells ceasing posterior to InC DL neurons (≈57.74%). In contrast, the treatment with pertussis toxin, a Gαi‐specific inhibitor [45], rarely exhibited any rescue effect (Figure 6B,C). Our data indicate that excessive activity of Gαs signaling in Nurr1‐deficient CLA neurons causes, at least in part, their migration into InC.

PKA signaling is the most common downstream cascade of Gαs [42, 46]. We found the phosphorylation levels of PKA substrates, but not PKC substrates (Figure S13A), were ectopically elevated in Nurr1‐deficient neurons (≈69.13%), in contrast to the very low levels in control brains (≈2.06%) (Figure 6D,E). Interestingly, one regulatory subunit of PKA complex, Prkar2b, was downregulated (Figure S10G–I), whereas one catalytic subunit, Prkacb, was upregulated in the claustro‐insular area of Nurr1‐deficient brains (Figure S13B). To investigate if CLA cell mislocalization in Nurr1‐deficient brains is linked to ectopic PKA activities, we prepared brain slice cultures from two hemispheres of the same brain and treated only one group with a cell‐permeable PKA inhibitor Rp‐cAMP [47]. PKA inhibition by Rp‐cAMP led to a larger population of Nurr1‐Cterm+ cells resident in CLA area in the treated mutant hemispheres (≈59.30%) than that of non‐treated ones (≈13.96%) (Figure 6F,G), mimicking the situation of NF449 treatment. Therefore, inhibition of PKA signaling is necessary to prevent CLA cells from migrating into InC territory. We next aimed to test if low PKA activity is also necessary for CLA cell specific gene expression. We performed IF for Nurr1‐Cterm, Rorβ, and Tle4 (marking the anatomic structures) in the cultured brain slices of varied conditions, and quantified the proportions of Nurr1‐Cterm+/Rorβ+ cells relative to all Nurr1‐expressing cells. While this ratio was ≈4.33% in the untreated control brains, it was significantly elevated to ≈28.54% ± 2.69% in untreated Nurr1‐deficient slices. Additionally, treatment with NF449 and Rp‐cAMP reduced the ratios to ≈10.55% ± 2.70% and ≈11.81% ± 3.11%, respectively (Figure 6H,I). Taken all together, the minimal activity of Gαs‐PKA signaling in CLA neurons is required for their appropriate settlement inside CLA and cell fate specification.

## 3 Discussion

There has been a longtime debate on if CLA is the main processor for mammalian consciousness. This attracted a constantly growing interest in CLA in the last two decades. CLA has been shown to be one of the most interconnected tissues to coordinate a wide range of sophisticated behaviors. However, molecular mechanisms underlying CLA formation and physiological functions remain little known. Here we report experimental evidences that Nurr1 is a master regulator to control CLA morphogenesis, cell identity determination, connectivity, and CLA‐dependent cognition.

### 3.1 Nurr1‐Deficient Mice As a Model to Investigate Forebrain Development and Evolution

The developmental origin of CLA has long been a subject of debate as well. Luis Puelles and Charles Watson recently proposed a ‘*tetrapartite pallium model*’ based on Nurr1 expression pattern, hypothesizing that principal CLA neurons derived from lateral pallial origin are born earlier and populate initially at the brain surface, and later‐born cells of the same origin pass CLA to form InC in an inside‐out manner similar to neocortex [1, 18]. This latest model differs from the old concept of ‘*claustro‐amygdaloid complex*’, however, direct experimental proof has been yet insufficient. Here we show that CLA neurons deficient for Nurr1 TF bypass CLA region and migrate along radial glia into InC. In parallel, these neurons downregulate CLA transcriptome but activate InC‐specific gene expression profile. These results uphold that CLA and InC neurons share the same homolog and migrate along the same radial columns, and that Nurr1 is the key factor to segregate the earlier born clusters (mainly at E12.5) [41] to form subcortical CLA from the later arriving InC neurons. Nevertheless, we did not observe any tangentially migrating Nurr1‐Cterm+ cells in mutant brains throughout our study, challenging the hypothesized viewpoint of ‘*tetrapartite pallium model*’ that some Nurr1+ CLA glutamatergic neurons may migrate tangentially to become dorsal Sp neurons or Arimatsu cells. One report demonstrated a tangentially migrating subpopulation of Dbx1 lineage of glutamatergic neurons originating from the pallial‐subpallial border [48], but the other researchers argued that these Dbx1‐expressing neurons were derived from local dorsal pallial progenitors and incorporated in the neocortex via stereotypical radial migration [49]. In our study, we did not observe any tangentially migrating neurons from the pallial‐subpallial border throughout our IUE assays, instead these neurons first followed ventral migration stream (VMS) along the external capsule and then turned to migrate radially to CLA or InC territory (Figure S14). Additionally, Nurr1‐deficient CLA cells constitutively migrated along a radial scaffold to InC after they left VMS and did not contribute to Sp, raising a question for more investigation if CLA is a spatially extended or independent structure of Sp despite their partially common evolutionary origin [19, 50].

The homologs of mammalian claustro‐insular complex have been suggested to be in the mesopallium of bird brains and the rostromedial part of pallial dorsal ventricular ridge (DVR) of reptile brains, respectively, in earlier descriptive anatomic studies [1, 51, 52]. Notably, Nurr1 was also the most frequently used signature gene to identify CLA neurons in Sauropsids, as in rodents and primates [1, 19, 50, 51, 52]. Latest advances in single cell transcriptomics corroborated the similarity of mammalian CLA proper to bird mesopallial or reptile DVR cell subpopulations by the means of spatial mapping in gene expression profiling [2, 52], though the evolvement of Sauropsids’ pallia may be more divergent from one‐to‐one correspondence in topographic subdivisions as compared to mammalian pallia and exhibit transcriptome convergence across cell types [53]. These findings indicate that CLA is one of the ancient forebrain structures already in the last stem amniote ancestor, and that Nurr1 expression, as well as possibly its downstream targets, is preserved in CLA cells during pallium evolution. This conservation, supported by our results, suggests that Nurr1‐dependent molecular pathways may serve as common paradigms in the regulation of CLA morphogenesis, pallial localization or interaction with neighboring tissues, among amniote species, highlighting the importance of our study in the framing of evo‐devo biology.

CLA is surrounded by striatum and Tle4+ InC DL neurons in mouse brains where a clear extreme capsule structure lacks. CLA cells in Nurr1‐deficient brains easily invade InC, whereas neighboring DL cells appear to occupy the original CLA territory. These results suggest that there is mutual repulsion between CLA and InC cells to delineate claustro‐insular structure, and that Nurr1 presides over the CLA cohort and its deficiency alone can disrupt the interaction. Notably, our transcriptomic data revealed a number of cell‐cell contact clues that were dysregulated due to Nurr1 deficiency, such as members in ephrin‐Eph and Semaphorin‐Plexin signaling. These proteins are often axon membrane‐bound and mediate repelling interplay *in trans* between contacted tissues [54, 55]. This may provide a hint for how CLA is silhouetted by white matter (external and extreme capsules) in human brains. Nurr1‐deficient mice thus may serve as a potent genetic model to better our insights into the development of various Nurr1+ forebrain structures, though further efforts are required.

Nurr1 can be the major regulator of CLA neuron fate specification, in that many CLA signature genes are downregulated while InC‐specific genes are ectopically upregulated in Nurr1‐deficient CLA cells. Others’ and our single‐cell transcriptomic analysis verified CLA‐specific gene expression was broadly affected due to Nurr1 deficiency [56]. These independent studies confirm the conclusion that Nurr1 is a key TF that controls the genetic program of CLA neuron identity. In our IUE experiments, not all cells carrying Nurr1 expressing constructs were converted into CLA neurons. This suggests that Nurr1 alone is necessary but may not be sufficient to determine CLA cell identity. Nurr1 is also strongly expressed in Sp and Sb, and has been long known as marker genes in these tissues, but its absence seems not to affect their cell identity or anatomic structure. Nurr1's role in the control of CLA cell fate is plausibly cell type‐ or region‐specific, and may depend on interaction with local environmental factors. This is also consistent with our findings that suppression of Gαs‐PKA signaling rescued both CLA cell positioning and fate specification.

### 3.2 The Connectivity and Behaviors Tied to Claustrum Depends on Its Structural Integrity Controlled by Nurr1

Amygdala‐CLA inter‐areal FC and emotion regulation also require CLA integrity regulated by Nurr1, supported by our observations that synchronous activities of amygdala network are impaired in Nurr1‐deficient mice when seeding CLA as ROI in rs‐fMRI, and that Nurr1‐deficient mice are less sensitive to stress‐elicited emotional responses. These findings are consistent with another report that CLA neuron inactivation led to increased resilience to stress‐responsive behaviors controlled by amygdala‐CLA processing circuit [8]. Discrepant observations exist on CLA's roles in attention and impulsivity modulation: some reported CLA negatively regulated attention [14] and facilitated impulsive trials [11, 14], however, others found CLA was supportive for attentional performances [9, 10] and reduced impulsive errors [12]. One of the explanations could be that distinct input‐output circuitries and circuit‐specific subsets of CLA neurons were assessed, such as those connected with ACC [10, 12], prefrontal cortex [14], auditory cortex [9] or motor cortex [11]. Here, we show genetic abolishment of the complete CLA glutamatergic ensemble and impairment of CLA‐ACC communication due to Nurr1 deficiency coincide with inadequate competence for attention and impulse control. This indicates that CLA per se is indeed required for optimization of both cognitive functions.

Notably, other neural networks linked to CLA were also impaired in our seed‐driven fMRI assay, such as the networks embedded in hippocampal formation, including the hippocampus proper, Sb, and entorhinal cortex (Figure 2H). The common feature of these structures is their involvement in spatial navigation and contextual memory consolidation [57, 58]. Some studies have indicated that claustro‐hippocampal or claustro‐entorhinal projections play important roles in these processes [59, 60]. Our results suggest that Nurr1 in CLA neurons may participate in the regulation of neural signal transmission to the hippocampal formation. Though one study previously reported that Nurr1 haploinsufficiency in rodents did not lead to a defect in spatial learning or memory formation [61], this can be explained by the fact that one allelic copy of Nurr1 is adequate to support its functionality in CLA neurons. Recent findings in CLA connectivity mapping of monkeys and humans have delineated the claustro‐cerebellum connections in primates [62, 63], but the authors claimed the absence of this interaction in rodents. Our results challenged their viewpoint by showing that CLA is functionally communicated with the cerebellar network in seed‐based fMRI analysis of our control mice, and that the synchronous activity of cerebellar network to CLA was visibly weakened in the Nurr1‐deficient mice (Figure 2H). The claustro‐cerebellar connectivity has been hypothesized to be involved in sociality and mentalization [62, 63], during which CLA serves as a broadcast hub to coordinate information from prefrontal networks to cerebellar neurons; however, more solid experimental evidence is still required in this newly emerging research. Our mice may provide a promising model to test these hypotheses and to investigate the molecular mechanisms underlying these cognitive behaviors. Collectively, Nurr1 is seated upstream of genetic hierarchy in CLA to control its development‐connectivity‐cognition dimension, in line with its life‐long expression in CLA.

### 3.3 Nurr1 Regulates Claustral Morphogenesis and Cell Fate via Gαs‐PKA Signaling

G‐protein‐mediated signaling is one of the major regulatory pathways in cell motility, but Gα‐subclass effectors play complex and contextually divergent roles in variable cell types [64]. Both Gαs and Gαi subunits have been shown to stimulate promigratory events in certain cell types but suppress cell migration in other circumstances, depending on their expression levels, homeostasis of Gαs/Gαi, and cell type‐specific niche [46, 64, 65, 66, 67, 68]. We thus utilized selective inhibitors to reveal Gαs‐PKA axis to be involved in the control of migration inhibition downstream of Nurr1 in CLA cells. PKA pathway, as one main responsive signaling of Gαs, serves as a central node to regulate cytoskeleton dynamics so as to coordinate almost all key events of cell migration, such as polarization, adhesive interaction with extracellular matrix, leading edge formation, and soma translocation [68, 69, 70]. For instance, Gαs‐PKA signaling facilitates cortical neuron migration by directly phosphorylating the microtubule‐associated protein Doublecortin (*Dcx*) to induce promigratory cytoskeleton reorganization [71]. Consistently, PKA substrate phosphorylation was strongly detected in radially migrating cortical neurons, but rarely in static CLA cells in control brains. In Nurr1‐deficient CLA cells, however, the PKA substrate phosphorylation level was substantially augmented. Despite the delicate spatio‐temporal control of PKA signaling in a motile cell in terms of its abundance, equilibrium of catalytic and regulatory subunits, activation timing and subcellular compartmentation, basic PKA activity is generally required [46, 68, 69, 70, 71, 72]. In this regard, minimizing PKA activity may be the most efficient implementation to prevent CLA cell migration into InC.

Nurr1 has been identified as an immediately early gene responsive to electronic or chemical stimuli. Multiple lines of evidence have indicated that Nurr1 expression can be activated or enhanced when exposed to a wide range of extracellular signals, such as growth factors, hormones, neurotransmitters, ions, et al., in diverse tissues [73]. In the nervous system, the activity dependence of Nurr1 expression has been mainly reported to be involved in the processes of memory formation and consolidation [74], or neuroprotection from aging or lesions [75] in postnatal mice. Activity‐induced upregulation of Nurr1 is mostly attributed to increased levels of intracellular cAMP due to stimuli, and consequent activities of PKA and cAMP‐response‐element‐binding‐protein (CREB) [74, 75, 76, 77]. Consistently, there is a functional ‘half‐CRE’ element responsive to CREB signaling in the Nurr1's promoter region [78]. Here, we report that Nurr1 may suppress the expression of G‐protein complex components and thus the levels of cAMP and PKA activity, suggesting a likely role of Nurr1 in the inhibitory feedback loop to cAMP‐PKA‐CREB axis when CREB activity is abundant. Whether Nurr1's basal expression is sufficient, or its activity‐dependent enhancement is required in embryonic neural development still lacks evidence and remains an interesting question for investigation.

Given the deep position and tight interplay with neighboring tissues of CLA in mouse brains, a cell type‐specific Cre line serves as a better tool for genetic study than artificial lesions [79], which debilitates complete and neat gene inactivation in CLA. However, the CLA‐specific Cre lines that have been reported so far trigger recombination either in a relatively small subset of CLA neurons (Egr2‐Cre) [9] or only in adult CLA (Tbx21‐Cre) [80]. Our study initiates a new avenue for cell‐type‐specific investigation of CLA.

## 4 Methods and Materials

### 4.1 Mice

Nurr1^flox/flox^; Emx1^cre/wt^ and Nurr1^flox/flox^; Nex^cre/wt^ mice were obtained by crossing Nurr1^flox/flox^ with Emx1^cre/wt^ and Nex^cre/wt^ mouse lines, respectively. The control mice used in this study were Cre‐negative Nurr1^flox/flox^ mice. The genotyping strategies for these lines were reported previously [17] and the genotyping primers are listed in Table S2. The term ‘Nurr1‐deficient mice’ in text means Nurr1 conditionally deficient mice recombined by Emx1^cre/wt^ (Nurr1^flox/flox^; Emx1^cre/wt^) unless noted otherwise. Experiments involving living mice conform to German regulatory standards and were approved by the authority (LaGeSo Berlin). The licenses for animal experiments are T0033 (sample collection) and G0184 (IUE).

### 4.2 Molecular Cloning and Constructs

The plasmid backbone used in this study is a Cre‐dependent bicistronic expression vector (pCAG‐FPF‐GFP) as previously described [81]. The full‐length open reading frames of Nurr1, Gnb4, and Gng2 with the kozak sequences were subcloned into pCAG‐FPF‐GFP plasmids by proper restriction enzymes. All cloned genes in this study were verified by sequencing. Cloning primers are listed in Table S2.

### 4.3 Immunofluorescence

The mouse brains for immunofluorescence (IF) were fixed in 4% paraformaldehyde (PFA, diluted in DEPC‐treated PBS, DPBS) overnight (O/N) and subsequently incubated in DPBS containing 15% for 6 h (hour) and 25% sucrose O/N at 4°C. The fixed brains were embedded in Tissue‐Tek OCT and sectioned at −20°C (16 µm in thickness). The brain sections were incubated in blocking solution (BS: 10% horse serum, 2% BSA, 0.5% Triton X‐100 in PBS) for 1 h following incubation with primary antibodies (in BS) O/N at 4°C. On the second day, the sections were incubated with secondary antibodies for 1 h. The sections were washed 3 times in 1x PBS between each incubation step (10 min per wash). Primary antibodies used in IF are listed in Table S3. Fluorescent secondary antibodies used in IF are all from Jackson ImmunoResearch (all were raised in donkey and diluted 1:500).

### 4.4 Nissl Staining

The embedded brains were cut into sagittal or coronal sections at a thickness of 16 µm and rehydrated in 1x PBS for 15 min. The sections were stained in 0.5% cresyl violet solution for 15 min at RT and washed 3 times with 1x PBS. Eventually, the stained sections were dehydrated in an ascending alcohol series (50%–80%–90%–100%, 5 min each) and 100% Xylol for 5 min, and then mounted in Permount toluene media.

### 4.5 In Situ Hybridization

The mouse brains for in situ hybridization (ISH) were processed as in IF. First Day: brain sections were dried in a vacuum for 30 min, fixed in 4% PFA (in DPBS) for 15 min, and incubated in proteinase K solution (20 mm Tris pH 7.5, 1 mm EDTA pH8.0, 20 µg/mL proteinase K) for 2.5 min. Subsequently, brain sections were washed in 0.2% Glycine (in DPBS), post‐fixed in 4% PFA containing 0.2% glutaraldehyde (SIGMA) for 15 min, and followed by prehybridized in hybridization buffer (HB: 50% deionized formamide, 5x SSC, 1% blocking reagent from Roche, 5 mm EDTA, 0.1% Tween20, 0.1% CHAPS, 100 µg/mL Heparin, 100 µg/mL yeast RNA, 50 µg/mL Salmon sperm DNA) at 65°C for 2 h. Eventually the slides carrying targeted probes (in HB) were incubated at 68°C O/N. The sections were washed twice in DPBS between each step (5 min per wash).

Second Day: the brain sections were washed once in 2x SSC, incubated in RNase solution (0.5 m NaCl, 10 mm Tris pH 8.0, 20 µg/mL RNase A) for 30 min at 37°C, washed once in 2x SSC, and then washed stringently 3 times in 50% formamide/ 2x SSC at 63°C (30 min each), and eventually washed 3 times in KTBT buffer (50 mm Tris pH7.5, 150 mm NaCl, 10 mm KCl, 1% Triton X‐100) for 10 min each. The sections were next incubated in blocking solution (BS, KTBT containing 20% sheep serum) for 2 h, followed by incubation with anti‐digoxigenin antibody (conjugated with alkaline phosphatase, 1:1500) in BS at 4°C O/N.

Third day: the brain sections were washed 3 times in KTBT for 30 min each, washed twice in NTMT buffer (100 mm Tris pH 9.5, 100 mm NaCl, 50 mm MgCl2, 0.1% Tween 20) for 15 min each, and were eventually incubated with NBT/BCIP substrates (in NTMT). The staining was monitored hourly until the signals showed up. The stained sections were subjected to an ascending alcohol series (50%–80%–90%–100%, 5 min each), clearing solution (benzyl alcohol: benzyl benzoate = 1: 2) for 15 min, and finally mounted in Permount toluene media.

### 4.6 Retrograde DiI Labeling

The mouse brains were fixed in 4% PFA at 4°C O/N, followed by two washes with 1x PBS. The lipophilic DiI crystal was carefully buried in the primary motor cortex (P12, intact brains), hypothalamus (P5 or P0, remove about 1/5 of caudal portions of brains in length to expose the hypothalamus) and thalamus (P5 or P0, sagittal hemispheres) of fixed brains, which were subsequently incubated in 1x PBS containing 0.2% sodium azide (NaN_3_) in the dark at 37°C for 3 weeks. The brains carrying DiI were sectioned at 200 µm in thickness by a vibratome, which were then analyzed by IF.

### 4.7 In Utero Electroporation

In utero electroporation (IUE) was performed as previously described [81]. During IUE, the pregnant mice carrying E12.5 embryos were kept lying down on a heating pad and anesthetized by constant inhalation of isoflurane blended with oxygen. Subcutaneous administration of tamgesic was done before the operation was started. The embryos were gently pulled out with ring‐headed forceps from an incision (*≈*15 mm) along the abdomen midline. DNA constructs (500 ng/µL each, mixed with fast green dye) in a glass capillary were enforced by vacuum pico‐pump into either side of the cerebral lateral ventricles. The electrodes were positioned ventro‐caudally to biauricular to target claustrum (anode at the side of injection). Electroporation was achieved by an electroporator (setup: 6 times pulses, 30 V voltage, 40 ms pulse duration, and 999 ms interval time). 1x PBS containing antibiotics (100 units/mL Penicillin‐Streptomycin) was applied to each operated embryo right after electroporation. After surgery, the mice were put back to an individual cage marked with electroporated genes and dates. The operated mice were monitored every day until sacrifice.

### 4.8 Organotypic Brain Slice Culture

The protocol was modified from previously described [82]. The embryonic brains at E15.5 were dissected out and separated into two hemispheres from the midline, which were placed on the sagittal plane and immediately embedded in low melting‐point agarose (kept at 45°C before use) in a plastic mold. The brains were sliced in cold Krebs buffer (12.6 mm NaCl, 0.25 mm KCl, 0.12 mm NaH_2_PO_4_, 0.21 mm CaCl_2_, 0.12 mm MgCl_2_, 100 mm HEPES buffer, 0.5 mg/mL Gentamicin, 100 units/mL Penicillin–Streptomycin) using a vibratome (300 µm in thickness). The brain slices were gently transferred by blunt‐ended microspatulas onto a Millicell cell culture insert (PET membrane, pore size 1 µm) in a well of 6‐well plate and incubated in 1 m MEM medium (10% fetal bovine serum, 0.5% Glucose,100 units/mL Penicillin–Streptomycin in MEM solution) at the bottom for 1 h in a cell culture incubator (37°C and 5% CO_2_). Subsequently, the slices were cultured in Neurobasal medium (5% fetal bovine serum, 2% B27 supplement, 1% N2 supplement, 0.5% Glucose, 1% Glutamine, 100 units/mL Penicillin‐Streptomycin in Neurobasal solution) with or without inhibitors in the incubator for 6 days. The medium was renewed every 24 h. The brain slices were fixed by 4% PFA O/N and washed in 1x PBS 3 times (1 h each) after culture. The tail of each embryo was collected for genotyping.

### 4.9 Functional Magnetic Resonance Imaging

1) Experimental procedures: anesthesia was induced by 2% isoflurane in a mixture of 80% air and 20% O2. A subcutaneous catheter was placed, and freely‐breathing animals were measured at 7 T (BioSpec 70/20 USR, Bruker, Germany) with a Tx/Rx 1H‐cryoprobe and ParaVision 6.0.1 software. The protocol consisted of localizer scans, B0 mapping and shimming, T2w MRI, and resting state functional magnetic resonance imaging (rs‐fMRI). A bolus of medetomidine (0.1 mg/kg subcutaneously, Cepetor, CP Pharma) was applied during B0 mapping, and isoflurane gradually reduced to 0.5% over 3–5 min and kept at least for 5 min at 0.5% isoflurane before the start of rs‐fMRI, adapting the protocol as previously reported [83]. During the switch of anesthesia regimes, T2w images were acquired with a 2D‐RARE sequence with repetition time/echo time (TR/TE) = 4300 ms / 33 ms, RARE factor 8, 2 averages, 40 contiguous axial slices with a slice thickness of 0.4 mm, field of view (FOV) = 19.2 × 19.2 mm^2^, matrix (MTX) = 192 × 192, bandwidth (BW) = 34.7 kHz and total acquisition time (TA) = 3:26 min. Rs‐fMRI images were acquired with a 2D gradient echo EPI sequence (TR/TE = 1000 ms / 13 ms, flip angle FA = 50°, 16 contiguous axial slices with a slice thickness of 0.75 mm, FOV = 19.2 × 12 mm^2^, matrix of 128 × 80, BW = 400 kHz, 300 repetitions, and TA = 5:00 min).

2) MRI data analysis: Rs‐fMRI data were preprocessed in RABIES (https://github.com/CoBrALab/RABIES) [84]. Briefly, data were corrected for motion, susceptibility distortion, and signal that was confound‐corrected via signal confound regression taking the signal from cerebral spinal fluid. The cleaned time courses were then processed using two different approaches. In the first approach, analysis within each functional network was carried out using the dual‐regression framework as described previously in the context of mice [85]. Briefly, spatially delineated non‐thresholded reference maps were used to extract a weighted average BOLD time course for each scan. A general linear model was used to regress these time courses into the individual scans, to obtain parameter estimate maps indicative of network strength at every voxel for each corresponding reference map. The spatial reference maps used to delineate networks to be tested in the dual‐regression analyses were 17 reference resting state networks (including piriform cortex, sensory cortex, motor cortex, barrel cortex, limbic cortex, visual cortex, auditory cortex, anterior cingulate cortex, retrosplenial cortex, hippocampus, striatum, amygdala, thalamus, et al.) as determined in a previous study with group independent component analysis (ICA) [86]. Voxel‐wise statistics on the parameter estimate maps from dual regression were performed using a permutation test and corrected for multiple comparisons using the threshold‐free cluster enhancement (TFCE) approach. For the analysis of connectivity changes in specific regions of interest (ROI), the mean signals of ROI were correlated with all other voxels in the brain in a seed‐based approach for each animal. Since the DSURQE mouse atlas underlying RABIES did not contain all ROI, a brain atlas was derived from the Allen brain atlas (CCFv3) containing a custom set of regions (such as claustrum, insular cortex, anterior cingulate cortex, orbital cortex, amygdala, prelimbic cortex, retrosplenial cortex, hippocampus, motor cortex, visual cortex, auditory cortex, et al.) and was registered to the RABIES template space using ANTx2 (https://github.com/ChariteExpMri/antx2) [87]. Voxel‐wise group statistics on the seed‐based connectivity maps were performed using a permutation test and corrected for multiple comparisons using the TFCE approach.

### 4.10 Single Cell mRNA Sequencing

The claustro‐insular portions (15 control and 18 Nurr1 mutant replicates) of isolated mouse cerebral cortices (amygdala, hippocampal, and posterior neocortical tissues were manually excluded) were dissociated using ‘Neural Tissue Dissociation Kit (P)’ (Miltenyi Biotec) containing 1x protease inhibitors (SIGMA) according to the manufacturer's instruction. The cell suspension was immediately incubated in ‘Nuclei EZ lysis buffer’ (SIGMA) for 5 min on ice and quickly passed through single cell strainers (pore size 70 mm). Approximately 10 000 nuclei were procured for single‐nuclei encapsulation using a 10X single cell controller. 10x Genomics 3' v3 gene expression libraries were prepared and sequenced on a single Novaseq sequencing system (S1 lane).

Computation was performed on the ‘high‐performance computing’ research cluster of the Berlin Institute of Health. Raw FASTQ files were aligned to the mouse mm39 (refdata‐GEX‐mm10‐2024‐A) genome using cell ranger 9.0.1 (10X Genomics). Counts were then read into R, and Seurat object created with min.cells = 3 and min.features = 200. Cells were filtered to exclude those with mitochondrial gene content higher than 5% and less than 400 features in quality. After quality filtering, suspected doublets were removed using ‘DoubletFinder’ with an optimal pK values determined per sample. Data were then integrated using the standard Seurat workflow by taking 2000 variable features, ScaleData with var.to.regress = batch, running UMAP, ‘FindNeighbors’ with dims = 1:30, and ‘FindClusters’ with resolution = 0.5. An initial unbiased cell annotation was then performed by running Azimuth with the available ‘mousecortexref’ reference for the mouse motor cortex [36].

Pyramidal neurons were then subset based on the initial Azimuth annotation, and additional removal of two populations of neurons with high Gad1/2 expression was determined to be interneurons. A pyramidal seurat object was created by taking 2000 variable features, ScaleData with var.to.regress = batch, running UMAP with n.neighbors 30, min.dist = 0.2, ‘FindNeighbors’ with dims = 1:30, and ‘FindClusters’ with resolution = 0.4. A curated cell type annotation for pyramidal neurons was informed by the initial Azimuth annotation and by known marker genes (supplement reference to pyramid marker gene UMAPS). Pyramidal clusters were annotated L2/3 IT, L5 IT, L5 ET, L5/6 NP, L6b, L6 CT, and L6 IT.  L6 IT subclass includes the subset of robust Nurr1+ cells. Differentially expressed genes (DEG) by genotype were determined using ‘FindMarkers’ within each cell type with logfc.threshold = 0.25 and min.pct = 0.1 (Table. S1). A gene set enrichment analysis (GSEA) within Nurr1+ subset was performed by first converting mouse gene symbols to human ones and next analyzing gene ranking using the ‘gseGO’ package (minGSSize = 50, maxGSSize = 500) from the ‘ClusterProfiler’ package. The resulting GSEA categories were plotted using ‘gseaplot2’ from ‘ClusterProfiler’ package, and the enrichment map was created using the ‘EnrichPlot’ package.

### 4.11 Animal Behavior Analysis

1) Open field: The open field test is used to assess general locomotor activity levels and anxiety in rodents. On the day of testing, the animals were moved to a neighboring holding room 30 min before the test in order to allow the animals to acclimate to the room conditions. Temperature was maintained between 20°C–24°C, humidity between 45%–65%, ventilation, noise intensity, and lighting intensity were also kept at appropriate levels for mice for the duration of the experiments (the same to elevated plus maze). The maze size is 50 cm × 50 cm, and the center size is 27 cm × 27 cm. The mouse was gently placed at the peripheral area when the test began. The program was set as a delay time (10 sec) followed with testing time (10 min). All its movement was recorded by a camera (video encoder) with “tracking mode” of the “Viewer” software (BIOBSERVE), which also carried out face‐tail validity, head direction, and body centroid (the same to elevated plus maze). The amount of time spent in the central zone of the arena, travel distance in the central and peripheral zones, number of visits to the center, and average velocity are measured. The apparatus was cleaned by 5% EtOH after each test. The test does not discriminate sexes.

2) Elevated plus maze: This test is used to assess anxiety‐related responses in rodents. The maze apparatus consists of 2 open arms and 2 closed arms, of which each is 50 cm in length and white in color. The barriers of closed arms are 15 cm in height. The far‐end 10 cm of the open arms is defined as a risk area. The maze is elevated on a 40 cm‐high platform. A mouse was gently placed at the joint area of open and closed arms (central area) in the beginning of a test and allowed to explore for 5 min. The mouse behaviors were also recorded the “Viewer” software. The amount of time spent in open and closed arms, as well as in rick zones, the travel distance in each area, the number of visits to each area, and average velocity were measured. The time and track length in risk zones were exclusive to those in open arms. The apparatus was cleaned by 5% EtOH after each test. The test does not discriminate sexes.

3) Five Choice serial reaction time task (5CSRTT): 5CSRTT using mouse nose pokes is designed to assess their cognitive functions, such as attention and impulsivity. Before the start of the testing day, animals were habituated to experimental conditions for 30 min in a room adjacent to the experimental room. Temperature was maintained between 20°C–24°C, humidity between 45–65%, ventilation, noise intensity, and lighting intensity were also kept at appropriate levels for mice during experiments. Mice were food‐restricted to 80% of their ad libitum body weight and trained over several days, with multiple pre‐training and training sessions to ensure task proficiency. Animals undergo all pre‐training, training, and testing using Imetronics operant chambers and nose poke walls. The pre‐training sessions begin with **Session 1 (Habituation)**, where animals undergo a 30 min session with 50 trials involving only food pellets, with no visual stimuli, and a variable interval (one pellet dispensed every 30 sec on average, with a minimum of 0 sec and a maximum of 60 sec). Animals must enter the reward tray 20 times before progressing. In **Sessions 2–3 (Habituation with Nose Poking)**, a 30 min session of 50 trials was conducted where all nose poke holes are illuminated, and a reward is dispensed when the animal pokes any of the holes. Animals must complete 20 stimulus touches on two consecutive days before advancing to the training phase. **Training sessions** were conducted in five stages, with gradually decreasing stimulus durations (32,16,8,6,4 sec). Each training session lasts 30 min and includes 50 trials with a 5‐sec intertrial interval (ITI), where the nose poke holes are lit up randomly, and animals must reach a criterion of ≥50 trials, ≥80% accuracy, and ≤20% omissions before progressing. **Test sessions** were conducted in **two conditions**: the **ITI Hold Test** where the ITI is randomized between 5, 10, 15, 20, and 25 s, and the **Stimuli Responsive Test** where stimulus durations are randomized between 5, 5.5, 6, 6.5, and 7 s, with both test conditions including 100 trials within a 30 min session. Throughout the procedure, the performances of each mouse, including accuracy, inaccuracy, omissions, and premature responses were recorded so as to assess sustained attention and cognitive flexibility.

### 4.12 Quantification and Statistics

Quantification between two groups (such as control and Nurr1‐deficient brains, untreated and treated brains in pharmacological assays) was analyzed with two‐sided *Student's t*‐test. The charts present mean values ± standard deviation (SD). The statistics was analyzed using Prism software (version 9). Significance: *p* < 0.0001, ^****^; *p* < 0.001, ^***^; *p* < 0.01, ^**^; *p* < 0.05, ^*^; *p* ≥ 0.05, ns.

## Author Contributions

K.Y., V.T. carried out conceptualization; K.Y. established the cellular and surgical methods, such as brain slice culture, IUE, et al. K.Y. and P.L. developed the molecular and cellular biological methods, such as ISH., IF., DiI labeling, et al. A.G.N. developed the work flowline for single cell transcriptomics. M.M., C.F., and P.B.S. introduced and upgraded the analytic pipeline of rs‐fMRI. K.Y., P.D., and M.L. developed the animal behavior tests.

K.Y. and P.L. performed molecular and cellular characterization of Nurr1‐deficient mouse model. K.Y. carried out IUE and brains slice culture experiments. K.Y. prepared the single cell suspension, and A.G.N. analyzed the single cell mRNA transcriptomic data. S.M. and M.F. performed rs‐fMRI examination. S.P.K. and P.B.S. analyzed the rs‐fMRI data. K.Y. and P.D. performed animal behavior tests under M.L.'s supervision, and K.Y. analyzed the statistical data. K.Y., V.T. carried out supervision; V.T., D.S., P.B.S. acquired funding; K.Y., V.T. wrote the original draft; K.Y., V.T., A.G.N., P.B.S., D.S. wrote, reviewed, and edited the final manuscript.

## Funding

Experiments on molecular characterization of the mouse model, sequencing, and animal behavior tests were financially supported by the German Research Foundation (DFG grant TA 303/14‐1 and DFG Germany's Excellence Strategy Exc‐2049‐390688087), the Ministry of Science and Higher Education of the Russian Federation (project no. FSWR‐2023‐0029). Experiments required for revision (e.g. IF for DiI+ brains) were partially financed by the Russian state program of the ‘Sirius’ Federal Territory ‘Scientific and technological development of the “Sirius” Federal Territory’ (Agreement No. 20–03 dated 27.09.2024). Rs‐fMRI analysis: funding was provided by the German Federal Ministry of Education and Research (BMBF) under the ERA‐NET NEURON scheme (01EW2305), and the German Research Foundation (DFG, project BO 4484/2‐1, Project‐ID 424778381‐TRR 295 ReTune and EXC‐2049‐390688087 NeuroCure).

## Conflicts of Interest

The authors declare no conflict of interest.

## Supporting information




**Supporting File 1**: advs73465‐sup‐0001‐SuppMat.pdf.

**Supporting File 2**: advs73465‐sup‐0002‐Figure S1.png.

**Supporting File 3**: advs73465‐sup‐0003‐Figure S2.png.

**Supporting File 4**: advs73465‐sup‐0004‐Figure S3.png.

**Supporting File 5**: advs73465‐sup‐0005‐Figure S4.png.

**Supporting File 6**: advs73465‐sup‐0006‐Figure S5.png.

**Supporting File 7**: advs73465‐sup‐0007‐Figure S6.png.

**Supporting File 8**: advs73465‐sup‐0008‐Figure S7.png.

**Supporting File 9**: advs73465‐sup‐0009‐Figure S8.png.

**Supporting File 10**: advs73465‐sup‐0010‐Figure S9.png.

**Supporting File 11**: advs73465‐sup‐0011‐Figure S10.png.

**Supporting File 12**: advs73465‐sup‐0012‐Figure S11.png.

**Supporting File 13**: advs73465‐sup‐0013‐Figure S12.png.

**Supporting File 14**: advs73465‐sup‐0014‐Figure S13.png.

**Supporting File 15**: advs73465‐sup‐0015‐Figure S14.png.

**Supporting File 16**: advs73465‐sup‐0016‐Table S1.docx.

**Supporting File 17**: advs73465‐sup‐0017‐Table S2.docx.

**Supporting File 18**: advs73465‐sup‐0018‐Table S3.docx.

## Data Availability

All data that are needed to evaluate the conclusions in the manuscript are presented in the paper or supplementary materials. The materials used in this study can be made available upon reasonable request and issuance of data sharing agreements between research institutions. The single cell transcriptomic data have been deposited in Gene Expression Omnibus and are available under accession GSE287618. The ‘R’ analysis script used for single cell mRNA sequencing is available (https://github.com/qoldt/Nurr1‐snRNAseq/blob/main/scNurr1.Rmd).

## References

[1] L. Puelles , “Current Status of the Hypothesis of a Claustro‐Insular Homolog in Sauropsids,” Brain, Behavior and Evolution 96 (2022): 212–241. 34753135 10.1159/000520742

[2] H. Norimoto , L. A. Fenk , H. H. Li , et al., “A Claustrum in Reptiles and Its Role in slow‐wave Sleep,” Nature 578 (2020): 413–418. 32051589 10.1038/s41586-020-1993-6

[3] J. B. Smith , A. K. Lee , and J. Jackson , “The Claustrum,” Current Biology 30 (2020): R1401–R1406. 33290700 10.1016/j.cub.2020.09.069

[4] F. C. Crick and C. Koch , “What Is the Function of the Claustrum?,” Philosophical Transactions of the Royal Society B: Biological Sciences 360 (2005): 1271–1279. 10.1098/rstb.2005.1661 PMC1569501 16147522

[5] Q. Wang , L. Ng , J. A. Harris , et al., “Organization of the Connections Between Claustrum and Cortex in the Mouse,” Journal of Comparative Neurology 525 (2017): 1317–1346. 27223051 10.1002/cne.24047 PMC5324679

[6] S. R. Krimmel , M. G. White , M. H. Panicker , F. S. Barrett , B. N. Mathur , and D. A. Seminowicz , “Resting state Functional Connectivity and Cognitive Task‐related Activation of the human Claustrum,” Neuroimage 196 (2019): 59–67. 30954711 10.1016/j.neuroimage.2019.03.075 PMC6629463

[7] H. Qadir , B. W. Stewart , J. W. VanRyzin , et al., “The Mouse Claustrum Synaptically Connects Cortical Network Motifs,” Cell Reports 41 (2022): 111860. 36543121 10.1016/j.celrep.2022.111860 PMC9838879

[8] M. Niu , A. Kasai , M. Tanuma , et al., “trum Mediates Bidirectional and Reversible Control of stress‐induced Anxiety Responses,” Science Advances 8 (2022): abi6375. 10.1126/sciadv.abi6375 PMC8932664 35302853

[9] G. Atlan , A. Terem , N. Peretz‐Rivlin , et al., “The Claustrum Supports Resilience to Distraction,” Current Biology 28 (2018): 2752–2762. 30122531 10.1016/j.cub.2018.06.068 PMC6485402

[10] M. G. White , M. Panicker , C. Mu , et al., “Anterior Cingulate Cortex Input to the Claustrum Is Required for Top‐Down Action Control,” Cell Reports 22 (2018): 84–95. 29298436 10.1016/j.celrep.2017.12.023 PMC5779631

[11] M. Chevée , E. A. Finkel , S. J. Kim , D. H. O'Connor , and S. P. Brown , “Neural Activity in the Mouse Claustrum in a Cross‐Modal Sensory Selection Task,” Neuron. 110 (2022): 486–501. 34863367 10.1016/j.neuron.2021.11.013 PMC8829966

[12] G. Atlan , N. Matosevich , N. Peretz‐Rivlin , et al., “Claustrum Neurons Projecting to the Anterior Cingulate Restrict Engagement During Sleep and Behavior,” Nature Communications 15 (2024): 5415. 10.1038/s41467-024-48829-6 PMC11208603 38926345

[13] H. Atilgan , M. Doody , D. K. Oliver , et al., “Human Lesions and Animal Studies Link the Claustrum to Perception, Salience, Sleep and Pain,” Brain 145 (2022): 1610–1623. 35348621 10.1093/brain/awac114 PMC9166552

[14] J. Liu , R. Wu , B. Johnson , J. Vu , C. Bass , and J. X. Li , “The Claustrum‐Prefrontal Cortex Pathway Regulates Impulsive‐Like Behavior,” The Journal of Neuroscience 39 (2019): 10071–10080. 31704786 10.1523/JNEUROSCI.1005-19.2019 PMC6978937

[15] J. Jackson , M. M. Karnani , B. V. Zemelman , D. Burdakov , and A. K. Lee , “Inhibitory Control of Prefrontal Cortex By the Claustrum,” Neuron 99 (2018): 1029–1039. 30122374 10.1016/j.neuron.2018.07.031 PMC6168643

[16] R. H. Zetterström , L. Solomin , L. Jansson , B. J. Hoffer , L. Olson , and T. Perlmann , “Dopamine Neuron Agenesis in Nurr1‐deficient Mice,” Science 276 (1997): 248–250. 9092472 10.1126/science.276.5310.248

[17] B. Kadkhodaei , T. Ito , E. Joodmardi , et al., “Nurr1 is Required for Maintenance of Maturing and Adult Midbrain Dopamine Neurons,” The Journal of Neuroscience 29 (2009): 15923–15932. 20016108 10.1523/JNEUROSCI.3910-09.2009 PMC6666174

[18] C. Watson and L. Puelles , “Developmental Gene Expression in the Mouse Clarifies the Organization of the Claustrum and Related Endopiriform Nuclei,” Journal of Comparative Neurology 525 (2017): 1499–1508. 27159785 10.1002/cne.24034

[19] H. Bruguier , R. Suarez , P. Manger , et al., “In Search of Common Developmental and Evolutionary Origin of the Claustrum and Subplate,” Journal of Comparative Neurology 528 (2020): 2956–2977. 32266722 10.1002/cne.24922

[20] J. A. Gorski , T. Talley , M. Qiu , L. Puelles , J. L. Rubenstein , and K. R. Jones , “Cortical Excitatory Neurons and Glia, but Not GABAergic Neurons, Are Produced in the Emx1‐expressing Lineage,” The Journal of Neuroscience 22 (2002): 6309–6314. 12151506 10.1523/JNEUROSCI.22-15-06309.2002 PMC6758181

[21] Á. J. García‐Yagüe , P. Rada , A. I. Rojo , I. Lastres‐Becker , and A. Cuadrado , “Nuclear Import and Export Signals Control the Subcellular Localization of Nurr1 Protein in Response to Oxidative Stress,” Journal of Biological Chemistry 288 (2013): 5506–5517. 23283970 10.1074/jbc.M112.439190 PMC3581400

[22] A. Hoerder‐Suabedissen and Z. Molnár , “Molecular Diversity of Early‐born Subplate Neurons,” Cerebral Cortex 23 (2013): 1473–1483. 22628460 10.1093/cercor/bhs137

[23] P. Arlotta , B. J. Molyneaux , J. Chen , J. Inoue , R. Kominami , and J. D. Macklis , “Neuronal Subtype‐specific Genes That Control Corticospinal Motor Neuron Development in Vivo,” Neuron 45 (2005): 207–221. 15664173 10.1016/j.neuron.2004.12.036

[24] J. V. Nielsen , M. Thomassen , K. Møllgård , J. Noraberg , and N. A. Jensen , “Zbtb20 defines a Hippocampal Neuronal Identity Through Direct Repression of Genes That Control Projection Neuron Development in the Isocortex,” Cerebral Cortex 24 (2014): 1216–1229. 23283686 10.1093/cercor/bhs400

[25] T. Shaker , G. J. Dagpa , V. Cattaud , et al., “A Simple and Reliable Method for Claustrum Localization Across Age in Mice,” Molecular Brain 17 (2024): 10. 38368400 10.1186/s13041-024-01082-w PMC10874566

[26] S. Goebbels , I. Bormuth , U. Bode , O. Hermanson , M. H. Schwab , and K. A. Nave , “Genetic Targeting of Principal Neurons in Neocortex and Hippocampus of NEX‐Cre Mice,” Genesis 44 (2006): 611–621. 17146780 10.1002/dvg.20256

[27] A. Watakabe , “In Situ Hybridization Analyses of Claustrum‐Enriched Genes in Marmosets,” Journal of Comparative Neurology 525 (2017): 1442–1458. 27098836 10.1002/cne.24021

[28] S. H. Kim , K. An , H. Namkung , et al., “Anterior Insula–Associated Social Novelty Recognition: Pivotal Roles of a Local Retinoic Acid Cascade and Oxytocin Signaling,” American Journal of Psychiatry 180 (2023): 305–317. 36128683 10.1176/appi.ajp.21010053 PMC13520127

[29] J. P. Christianson , “An Insula‐Enriched Regulator of Retinoic Acid Marks a New Intersection in the Neural Circuitry of Mouse Social Behavior,” American Journal of Psychiatry 180 (2023): 262–264. 37002691 10.1176/appi.ajp.20230110

[30] L. C. Greig , M. B. Woodworth , M. J. Galazo , H. Padmanabhan , and J. D. Macklis , “Molecular Logic of Neocortical Projection Neuron Specification, Development and Diversity,” Nature Reviews Neuroscience 14 (2013): 755–769. 24105342 10.1038/nrn3586 PMC3876965

[31] D. A. Gehrlach , C. Weiand , T. N. Gaitanos , et al., “A whole‐brain Connectivity Map of Mouse Insular Cortex,” Elife 9 (2020): 55585. 10.7554/eLife.55585 PMC7538160 32940600

[32] V. N. Nikolenko , N. A. Rizaeva , N. M. Beeraka , et al., “The Mystery of Claustral Neural Circuits and Recent Updates on Its Role in Neurodegenerative Pathology,” Behavioral and Brain Functions 17 (2021): 8. 34233707 10.1186/s12993-021-00181-1 PMC8261917

[33] H. Wright , X. Li , N. B. Fallon , et al., “Differential Effects of Hunger and Satiety on Insular Cortex and Hypothalamic Functional Connectivity,” European Journal of Neuroscience 43 (2016): 1181–1189. 26790868 10.1111/ejn.13182 PMC4982083

[34] Q. Wang , Y. Wang , H. C. Kuo , et al., “Regional and Cell‐type‐specific Afferent and Efferent Projections of the Mouse Claustrum,” Cell Reports 42 (2023): 112118. 36774552 10.1016/j.celrep.2023.112118 PMC10415534

[35] C. Fernandes and S. E. File , “The Influence of Open Arm Ledges and Maze Experience in the Elevated Plus‐maze,” Pharmacology Biochemistry and Behavior 54 (1996): 31–40. 8728536 10.1016/0091-3057(95)02171-x

[36] Z. Yao , H. Liu , F. Xie , et al., “A Transcriptomic and Epigenomic Cell Atlas of the Mouse Primary Motor Cortex,” Nature 598 (2021): 103–110. 34616066 10.1038/s41586-021-03500-8 PMC8494649

[37] Y. Gao , C. T. J. van Velthoven , C. Lee , et al., “Continuous Cell‐type Diversification in Mouse Visual Cortex Development,” Nature 647 (2025): 127–142. 41193844 10.1038/s41586-025-09644-1 PMC12589121

[38] Y. Lei , Y. Liu , M. Wang , et al., “Single‐cell Spatial Transcriptome Atlas and Whole‐brain Connectivity of the macaque Claustrum,” Cell 188 (2025): 3863–3863. 40185102 10.1016/j.cell.2025.02.037

[39] D. Rosskopf , C. Nikula , I. Manthey , et al., “The Human G Protein β4 Subunit: Gene Structure, Expression, Gγ and Effector Interaction,” FEBS Letters 544 (2003): 27–32. 12782285 10.1016/s0014-5793(03)00441-1

[40] B. Stallmeyer , J. Kuß , S. Kotthoff , et al., “A Mutation in the G‐Protein Gene GNB2 Causes Familial Sinus Node and Atrioventricular Conduction Dysfunction,” Circulation Research 120 (2017): e33–e44. 28219978 10.1161/CIRCRESAHA.116.310112

[41] A. Hoerder‐Suabedissen , G. Ocana‐Santero , T. H. Draper , et al., “Temporal Origin of Mouse Claustrum and Development of Its Cortical Projections,” Cerebral Cortex 33 (2023): 3944–3959. 36104852 10.1093/cercor/bhac318 PMC10068282

[42] M. Jo and S. T. Jung , “Engineering therapeutic antibodies targeting G‐protein–coupled receptors,” Experimental & Molecular Medicine 48 (2016): 207. 10.1038/emm.2015.105 PMC4892866 26846450

[43] X. Zhang , M. Candas , N. B. Griko , R. Taussig , and L. A. J. Bulla , “A Mechanism of Cell Death Involving an Adenylyl Cyclase/PKA Signaling Pathway Is Induced by the Cry1Ab Toxin of Bacillus thuringiensis,” Proceedings of the National Academy of Sciences U S A. 103 (2006): 9897–9902. 10.1073/pnas.0604017103 PMC1502550 16788061

[44] P. Lin , T. Cheng , S. Ma , et al., “Bacillus Bombysepticus α‐Toxin Binding to G Protein‐Coupled Receptor Kinase 2 Regulates cAMP/PKA Signaling Pathway to Induce Host Death,” PLOS Pathogens 12 (2016): 1005527. 10.1371/journal.ppat.1005527 PMC4811588 27022742

[45] D. R. Littler , S. Y. Ang , D. G. Moriel , et al., “Structure–function analyses of a pertussis‐Like toxin From pathogenic Escherichia Coli Reveal a Distinct Mechanism of Inhibition of Trimeric G‐Proteins,” Journal of Biological Chemistry 292 (2017): 15143–15158. 28663369 10.1074/jbc.M117.796094 PMC5592689

[46] D. J. Ramms , F. Raimondi , N. Arang , F. W. Herberg , S. S. Taylor , and J. S. Gutkind , “Gαs–Protein Kinase A (PKA) Pathway Signalopathies: The Emerging Genetic Landscape and Therapeutic Potential of Human Diseases Driven by Aberrant Gαs‐PKA Signaling,” Pharmacological Reviews 73 (2021): 1326–1368. 10.1124/pharmrev.120.000269 PMC11060502 34663687

[47] B. T. Gjertsen , G. Mellgren , A. Otten , et al., “Novel (Rp)‐cAMPS Analogs as Tools for Inhibition of cAMP‐kinase in Cell Culture,” Journal of Biological Chemistry 270 (1995): 20599–20607. 7657638 10.1074/jbc.270.35.20599

[48] A. Teissier , A. Griveau , L. Vigier , T. Piolot , U. Borello , and A. Pierani , “A Novel Transient Glutamatergic Population Migrating From the Pallial–Subpallial Boundary Contributes to Neocortical Development,” The Journal of Neuroscience 30 (2010): 10563–10574. 20685999 10.1523/JNEUROSCI.0776-10.2010 PMC6634670

[49] E. Rueda‐Alaña , I. Martínez‐Garay , J. M. Encinas , Z. Molnár , and F. García‐Moreno , “Dbx1‐Derived Pyramidal Neurons Are Generated Locally in the Developing Murine Neocortex,” Frontiers in Neuroscience 12 (2018): 792. 30429769 10.3389/fnins.2018.00792 PMC6220037

[50] H. Li , A. Duque , and P. Rakic , “Origin and Development of the Claustrum in Rhesus Macaque,” Proceedings of the National Academy of Sciences U S A. 120 (2023): 2220918120. 10.1073/pnas.2220918120 PMC10334778 37406098

[51] L. Puelles , A. Ayad , A. Alonso , et al., “Selective Early Expression of the Orphan Nuclear Receptor Nr4a2 Identifies the Claustrum Homolog in the avian Mesopallium: Impact on Sauropsidian/Mammalian Pallium Comparisons,” Journal of Comparative Neurology 524 (2016): 665–703. 26400616 10.1002/cne.23902

[52] M. A. Tosches , T. M. Yamawaki , R. K. Naumann , A. A. Jacobi , G. Tushev , and G. Laurent , “Evolution of Pallium, Hippocampus, and Cortical Cell Types Revealed by Single‐cell Transcriptomics in Reptiles,” Science 360 (2018): 881–888. 29724907 10.1126/science.aar4237

[53] B. Zaremba , A. Fallahshahroudi , C. Schneider , et al., “Developmental Origins and Evolution of Pallial Cell Types and Structures in Birds,” Science 387 (2025): adp5182. 10.1126/science.adp5182 39946461

[54] M. C. Halloran and M. A. Wolman , “Repulsion or Adhesion: Receptors Make the Call,” Current Opinion in Cell Biology 18 (2006): 533–540. 16930978 10.1016/j.ceb.2006.08.010

[55] D. Rozbesky and E. Y. Jones , “Cell Guidance Ligands, Receptors and Complexes—orchestrating Signalling in Time and Space,” Current Opinion in Structural Biology 61 (2020): 79–85. 31862615 10.1016/j.sbi.2019.11.007 PMC7171467

[56] I. Mantas , I. Flais , Y. Masarapu , et al., “Claustrum and Dorsal Endopiriform Cortex Complex Cell‐identity Is Determined by Nurr1 and Regulates Hallucinogenic‐Like States in Mice,” Nature Communications 15 (2024): 8176. 10.1038/s41467-024-52429-9 PMC11408527 39289358

[57] B. L. McNaughton , F. P. Battaglia , O. Jensen , E. I. Moser , and M. B. Moser , “Path Integration and the Neural Basis of the 'cognitive Map',” Nature Reviews Neuroscience 7 (2006): 663–678. 16858394 10.1038/nrn1932

[58] G. Buzsáki and E. I. Moser , “Memory, Navigation and Theta Rhythm in the Hippocampal‐entorhinal System,” Nature Neuroscience 16 (2013): 130–138. 23354386 10.1038/nn.3304 PMC4079500

[59] C. Medina , S. O. Ramos , A. M. Depino , A. G. Romano , M. C. Krawczyk , and M. M. Boccia , “The Role of the Claustrum in the Acquisition, Consolidation and Reconsolidation of Memories in Mice,” Sci Rep. 14 (2024): 24409. 39420041 10.1038/s41598-024-74419-z PMC11487015

[60] T. Kitanishi and N. Matsuo , “Organization of the Claustrum‐to‐Entorhinal Cortical Connection in Mice,” The Journal of Neuroscience 37 (2017): 269–280. 28077707 10.1523/JNEUROSCI.1360-16.2016 PMC6596572

[61] F. Montarolo , S. Martire , S. Perga S , et al., “NURR1 deficiency Is Associated to ADHD‐Like Phenotypes in Mice,” Translational Psychiatry 9 (2019): 207. 31455763 10.1038/s41398-019-0544-0 PMC6712038

[62] E. J. Holzscherer , A. Zanini , C. Y. Liu , S. Everling , and D. A. Seminowicz , “Resting‐state Functional Connectivity of the Marmoset Claustrum,” Imaging Neuroscience 3 (2025): 109. 10.1162/IMAG.a.109 PMC12344515 40808791

[63] L. Rodríguez‐Vidal , S. Alcauter , and F. A. Barrios , “The Functional Connectivity of the human Claustrum, According to the Human Connectome Project Database,” PLoS ONE 19 (2024): 0298349. 10.1371/journal.pone.0298349 PMC11025802 38635579

[64] M. Cotton and A. Claing , “G protein‐Coupled Receptors Stimulation and the Control of Cell Migration,” Cellular Signalling 21 (2009): 1045–1053. 19249352 10.1016/j.cellsig.2009.02.008

[65] C. R. Surve , J. Y. To , S. Malik , M. Kim , and A. V. Smrcka , “Dynamic Regulation of Neutrophil Polarity and Migration by the Heterotrimeric G Protein Subunits Gαi‐GTP and Gβγ,” Science Signaling 9 (2016): ra22. 26905427 10.1126/scisignal.aad8163 PMC6364554

[66] J. Xu , F. Wang , A. V. Keymeulen , et al., “Divergent Signals and Cytoskeletal Assemblies Regulate Self‐organizing Polarity in Neutrophils,” Cell 114 (2003): 201–214. 12887922 10.1016/s0092-8674(03)00555-5

[67] B. D. Thompson , Y. Jin , K. H. Wu , et al., “Inhibition of Gαi2 Activation by Gαi3 in CXCR3‐mediated Signaling,” Journal of Biological Chemistry 282 (2007): 9547–9555. 17289675 10.1074/jbc.M610931200 PMC2366813

[68] A. K. Howe , “Regulation of Actin‐based Cell Migration by cAMP/PKA,” Biochimica Et Biophysica Acta (BBA)—Molecular Cell Research 1692 (2004): 159–174. 15246685 10.1016/j.bbamcr.2004.03.005

[69] K. A. Newell‐Litwa and A. R. Horwitz , “Cell Migration: PKA and RhoA Set the Pace,” Current Biology 21 (2011): R596–R598. 21820627 10.1016/j.cub.2011.06.032

[70] J. Stoufflet , M. Chaulet , M. Doulazmi , et al., “Primary Cilium‐dependent cAMP/PKA Signaling at the Centrosome Regulates Neuronal Migration,” Science Advances 6 (2020): aba3992. 10.1126/sciadv.aba3992 PMC7467704 32917588

[71] M. Toriyama , N. Mizuno , T. Fukami , et al., “Phosphorylation of Doublecortin by Protein Kinase A Orchestrates Microtubule and Actin Dynamics to Promote Neuronal Progenitor Cell Migration,” Journal of Biological Chemistry 287 (2012): 12691–12702. 22367209 10.1074/jbc.M111.316307 PMC3339951

[72] K. V. Svec and A. K. Howe , “Protein Kinase A in Cellular Migration—Niche Signaling of a Ubiquitous Kinase,” Frontiers in Molecular Biosciences 9 (2022): 953093. 35959460 10.3389/fmolb.2022.953093 PMC9361040

[73] M. A. Maxwell and G. E. Muscat , “The NR4A Subgroup: Immediate Early Response Genes With Pleiotropic Physiological Roles,” Nuclear Receptor Signaling 4 (2006): 002. 10.1621/nrs.04002 PMC1402209 16604165

[74] J. Català‐Solsona , A. J. Miñano‐Molina , and J. Rodríguez‐Álvarez , “Nr4a2 Transcription Factor in Hippocampal Synaptic Plasticity, Memory and Cognitive Dysfunction: A Perspective Review,” Frontiers in Molecular Neuroscience 14 (2021): 786226. 34880728 10.3389/fnmol.2021.786226 PMC8645690

[75] N. Volakakis , B. Kadkhodaei , E. Joodmardi , et al., “NR4A orphan Nuclear Receptors as Mediators of CREB‐Dependent Neuroprotection,” Proceedings of the National Academy of Sciences U S A. 107 (2010): 12317–12322. 10.1073/pnas.1007088107 PMC2901488 20566846

[76] M. S. Bridi , J. D. Hawk , S. Chatterjee , S. Safe , and T. Abel , “Pharmacological Activators of the NR4A Nuclear Receptors Enhance LTP in a CREB/CBP‐Dependent Manner,” Neuropsychopharmacology 42 (2017): 1243–1253. 27834392 10.1038/npp.2016.253 PMC5437882

[77] L. Wu , J. Liu , P. Gao , et al., “Transforming Activity of MECT1‐MAML2 Fusion Oncoprotein is Mediated by Constitutive CREB Activation,” The EMBO Journal 24 (2005): 2391–2402. 15961999 10.1038/sj.emboj.7600719 PMC1173159

[78] X. Zhang , D. T. Odom , S. H. Koo , et al., “Genome‐wide Analysis of cAMP‐response Element Binding Protein Occupancy, Phosphorylation, and Target Gene Activation in human Tissues,” Proceedings of the National Academy of Sciences U S A. 102 (2005): 4459–4464. 10.1073/pnas.0501076102 PMC555478 15753290

[79] T. Gu , J. Dong , J. Ge , et al., “Neurotoxic Lesions of the Anterior Claustrum Influence Cued Fear Memory in Rats,” Frontiers in Psychiatry 15 (2024): 1387507. 38707622 10.3389/fpsyt.2024.1387507 PMC11066318

[80] K. Narikiyo , R. Mizuguchi , A. Ajima , et al., “The Claustrum Coordinates Cortical Slow‐wave Activity,” Nature Neuroscience 23 (2020): 741–753. 32393895 10.1038/s41593-020-0625-7

[81] K. Yan , I. Bormuth , O. Bormuth , et al., “TrkB‐dependent EphrinA Reverse Signaling Regulates Callosal Axon Fasciculate Growth Downstream of Neurod2/6,” Cerebral Cortex 33 (2023): 1752–1767. 35462405 10.1093/cercor/bhac170

[82] J. M. Clegg and T. Pratt , “Organotypic Slice Culture of the Embryonic Mouse Brain,” Bio‐Protocol 10 (2020): 3674. 10.21769/BioProtoc.3674 PMC7842632 33659344

[83] M. Aswendt , U. Wilhelmsson , F. Wieters , et al., “Reactive Astrocytes Prevent Maladaptive Plasticity After Ischemic Stroke,” Progress in Neurobiology 209 (2022): 102199. 34921928 10.1016/j.pneurobio.2021.102199

[84] G. Desrosiers‐Grégoire , G. A. Devenyi , J. Grandjean , and M. M. Chakravarty , “A Standardized Image Processing and Data Quality Platform for Rodent fMRI,” Nature Communications 15 (2024): 6708. 10.1038/s41467-024-50826-8 PMC11306392 39112455

[85] J. Grandjean , D. Buehlmann , M. Buerge , et al., “Psilocybin Exerts Distinct Effects on Resting state Networks Associated With Serotonin and Dopamine in Mice,” Neuroimage 225 (2021): 117456. 33069863 10.1016/j.neuroimage.2020.117456

[86] V. Zerbi , J. Grandjean , M. Rudin , and N. Wenderoth , “Mapping the Mouse Brain With rs‐fMRI: An Optimized Pipeline for Functional Network Identification,” Neuroimage 123 (2015): 11–21. 26296501 10.1016/j.neuroimage.2015.07.090

[87] S. Koch , S. Mueller , M. Foddis , et al., “Atlas Registration for Edema‐corrected MRI Lesion Volume in Mouse Stroke Models,” Journal of Cerebral Blood Flow & Metabolism 39 (2019): 313–323. 28829217 10.1177/0271678X17726635 PMC6360485

