# Supplementary material for: Nurr1 Orchestrates Claustrum Development and Functionality
Source: Adv Sci (Weinh). 2025 Dec 22;13(13):e08999. doi: 10.1002/advs.202508999 (PMC12955989; doi:10.1002/advs.202508999)
Supplement: Supplementary file 1 — Supporting File 1: advs73465‐sup‐0001‐SuppMat.pdf. [file ADVS-13-e08999-s014.pdf]

Supplementary Materials for

Nurr1 Orchestrates Claustrum Development and Functionality

Kuo Yan, *et al*., Victor Tarabykin

*Corresponding authors: Kuo Yan, kyan423@hotmail.com;
Victor, Tarabykin, tarabykin.vs@talantiuspeh.ru

This file includes:

Supplementary figures S1 to S14 (Page 2 - 17)

Tables S1 to S3 (Page 18 - 20)

Supplementary figures and captions

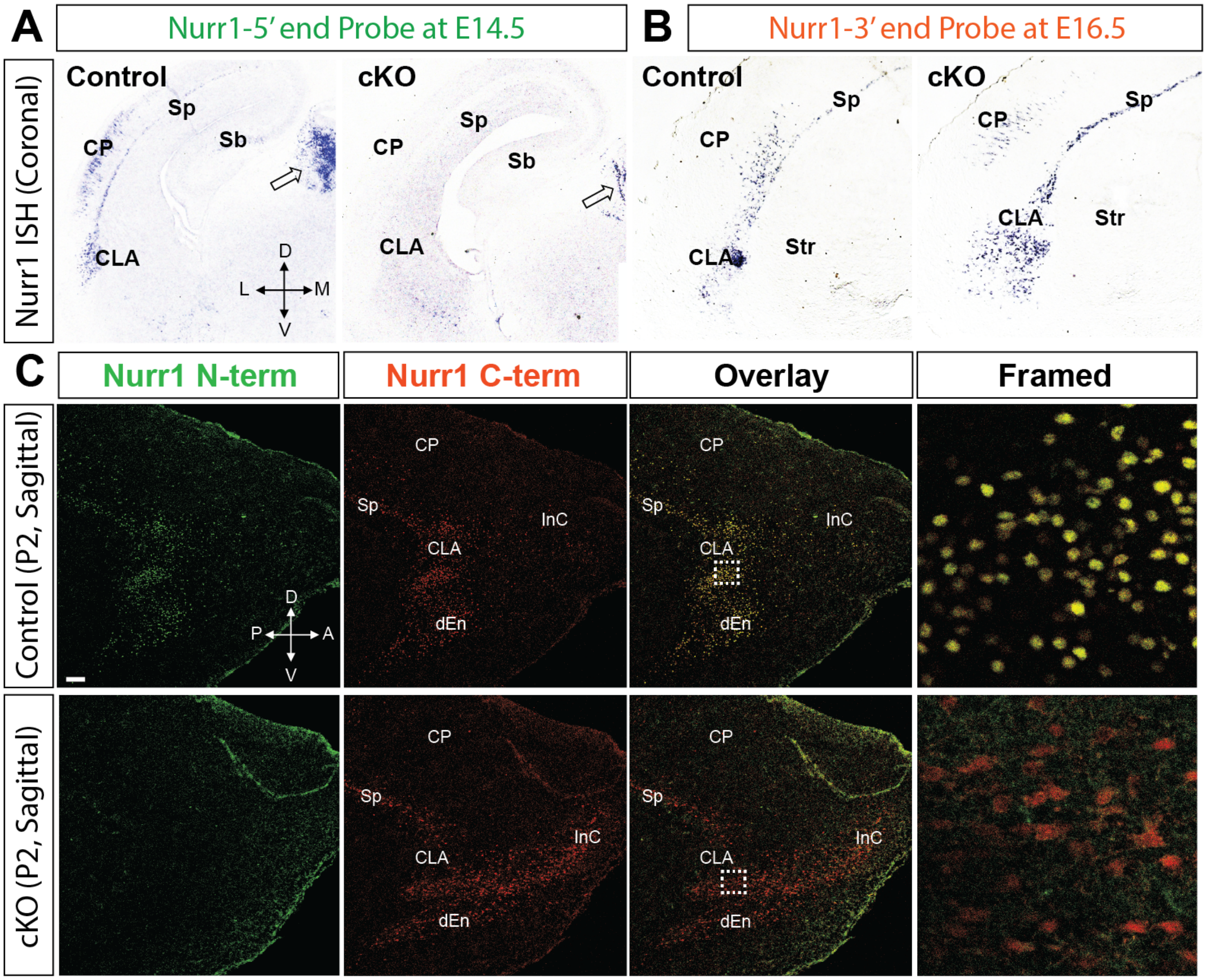

Figure S1 C-terminal truncate polypeptide of Nurr1 is traceable by antibody and *in situ* hybridization probe in Nurr1 lineage cells deficient for transcription regulation.
(A, B) *In situ* hybridization (ISH) using the Nurr1 5'end probe (**A**, at E14.5) or Nurr1 3'end probe (**B**, at E16.5) on coronal sections of control and Emx1$^{Cre/Wt}$ Nurr1 conditional deficient (cKO) brains shows Nurr1 is expressed in the developing subiculum (Sb), subplate (Sp), claustrum (CLA) and cortical plate (CP) in control mice, but ISH signal is undetectable in the cortex of Nurr1 cKO brains. The arrowheads indicate ISH signals in the habenula of control and Nurr1 cKO brains as internal reference. **(B)** The ISH signals of Nurr1-3'end probe are detectable in both control and cKO brains. Str, striatum; D, dorsal; M, medial; V, ventral; L, lateral.
(C) Immunofluorescence (IF) on sagittal sections of control and Nurr1 cKO brains at P2 shows that the Nurr1-Nterm and Nurr1-Cterm antibodies (ab) label the neurons in CLA, dorsal endopiriform nucleus (dEn) and Sp in control mice. The Nurr1-Nterm ab shows negative signal in cKO cortex, however, Nurr1-Cterm ab remains detectable signals in CLA, dEn and Sp, consistent with ISH. The overlay images show that all Nurr1-Cterm+ CLA cells are double positive with Nurr1-Nterm ab signals in controls, suggesting the high specificity of both ab. Nurr1-Cterm polypeptide displays cytoplasmic localization due to loss of its nuclear localization signal (287 – 314 aa). The framed images show magnification views of the dotted-line boxed areas (the same below). InC, insular cortex. A, anterior; P, posterior. Scale bar: 200 µm.

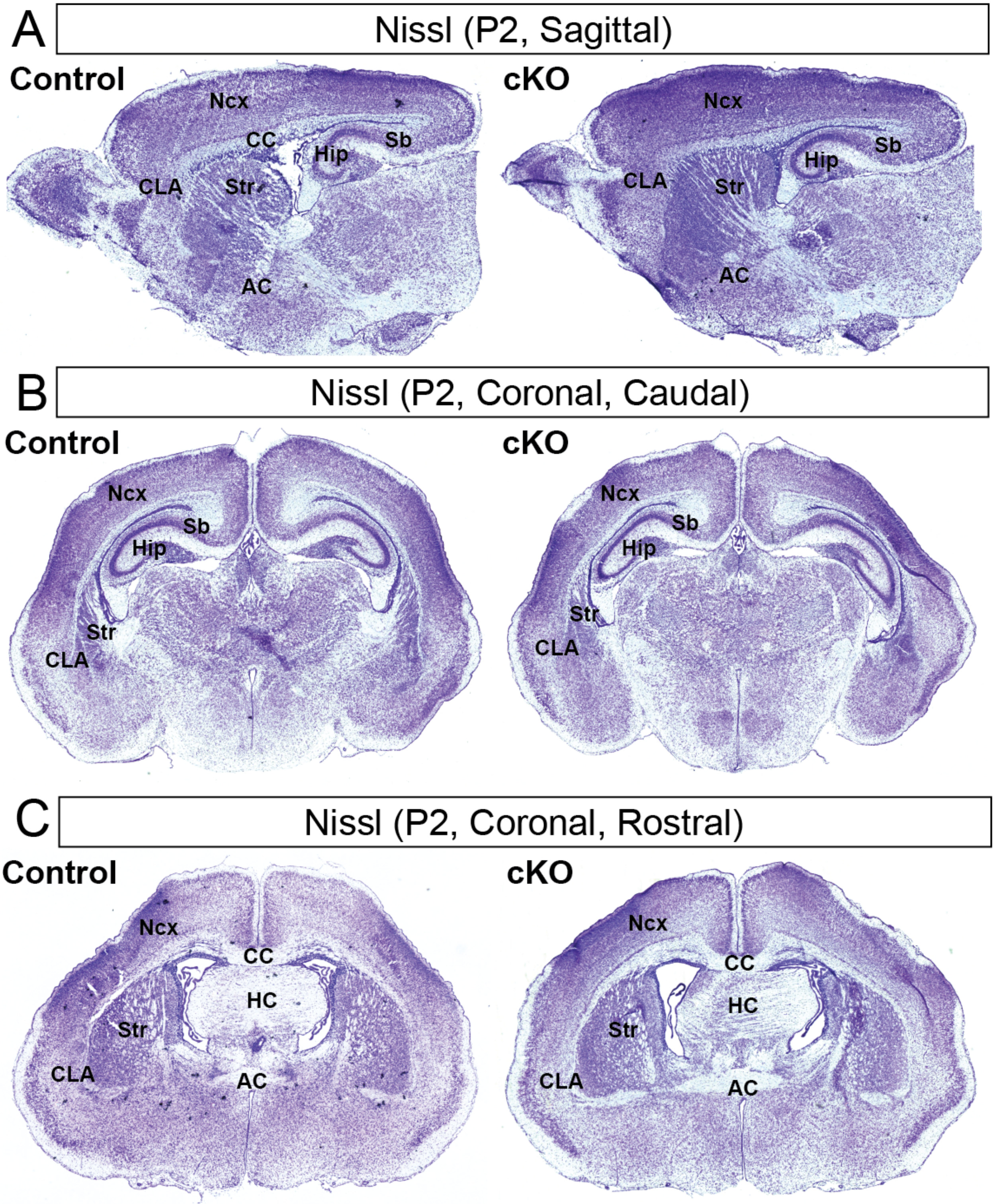

Figure S2 Nurr1 deficiency does not affect morphology of subplate and subiculum. **(A-C)** Nissl staining on sagittal (**A**), caudal (**B**) and rostral (**C**) coronal sections of control and Nurr1 deficient brains shows that the main cerebral structures of Nurr1 deficient mice are basically normal. Hip, hippocampus; Ncx, neocortex; AC, anterior commissure; CC, corpus callosum.

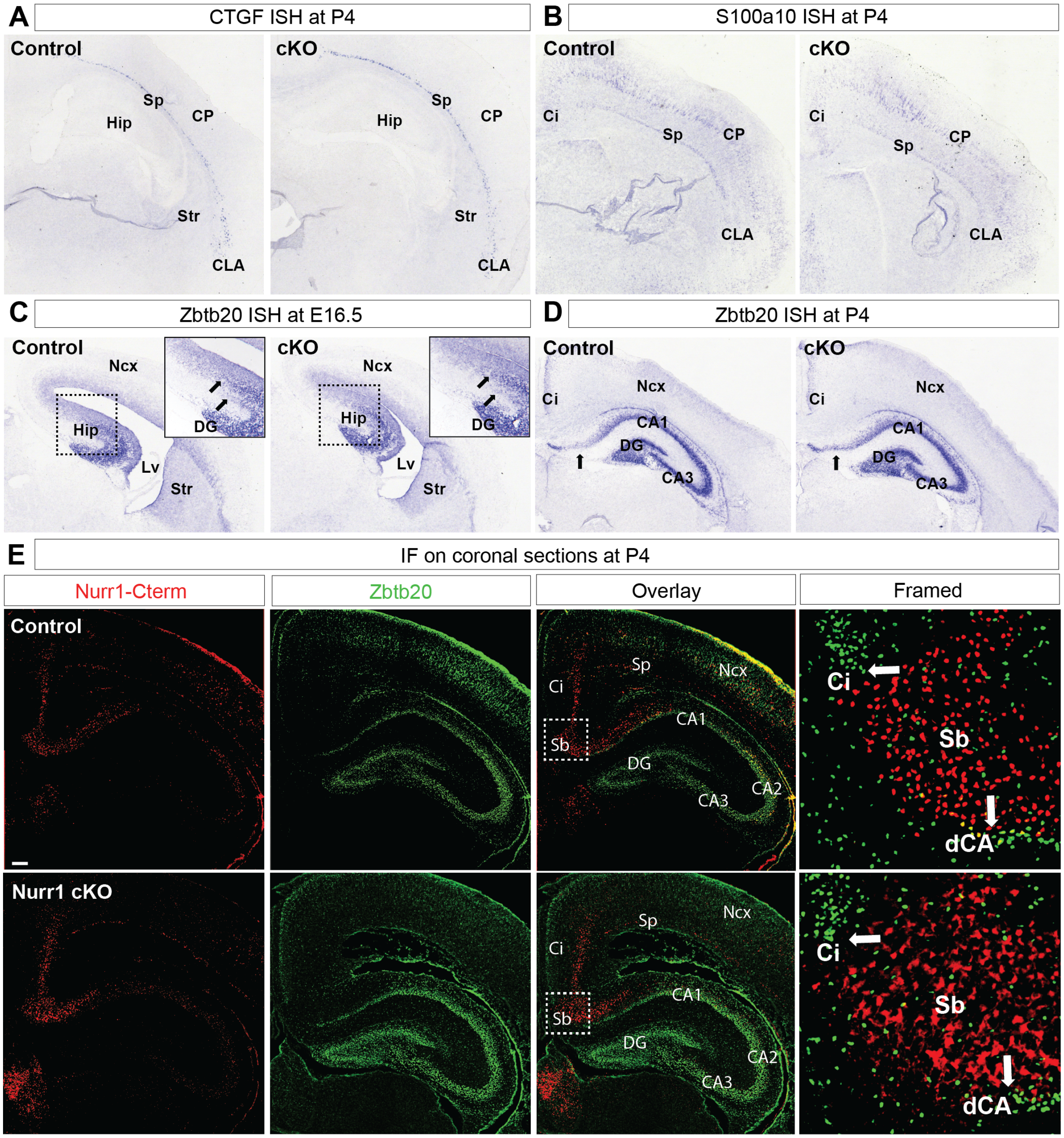

Figure S3 Nurr1 deficiency does not affect subplate and subiculum morphology.
(A, B) ISH using the CTGF (**A**) and S100a10 (**B**) probes on coronal sections of control and Nurr1 cKO brains at P4 shows that the Sp is not disrupted in mutant brains. Ci, cingulate cortex.
(C, D) ISH using the Zbtb20 probe on coronal sections of control and Nurr1 cKO brains at E16.5 (**C**) and P4 (**D**) shows that the borders between Ncx and Hip are unaltered in Nurr1 deficient brains. The arrowheads in (**C, D**) indicate the neocortico-hippocampal borders. Lv, lateral ventricle; DG, dentate gyrus; CA1 and CA3 indicate the *Ammon's* horn subareas.
(E) IF for Nurr1-Cterm and Zbtb20 on coronal sections of control and Nurr1 cKO brains at P4 shows that Nurr1 expression in Sb separates hippocampal from neocortical structures in both control and Nurr1 deficient brains. The framed images show magnification views of the boxed areas in overlay. The arrowheads indicate the neocortico-hippocampal borders. dCA, distal area of *Ammon's* horn CA1. Scale bar: 200 µm.

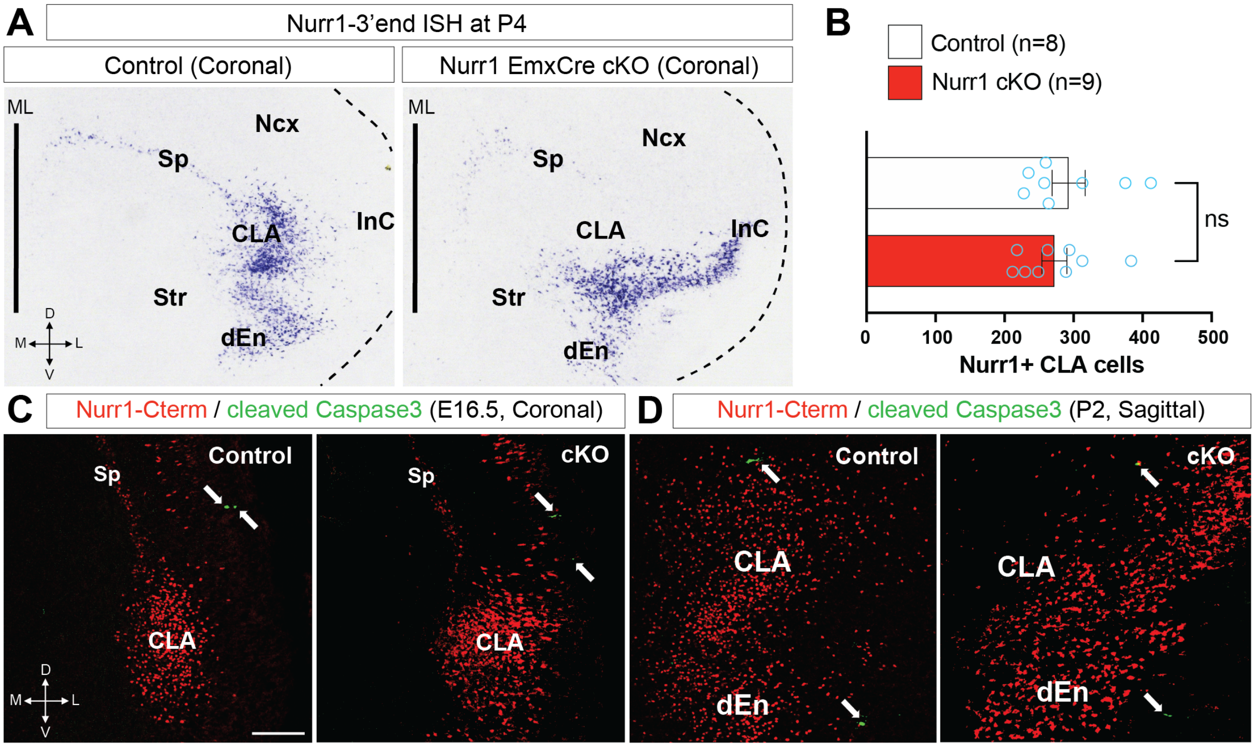

Figure S4 Nurr1 regulates claustrum morphogenesis, but not claustral cell production or survival.

(A) ISH using the Nurr1 3’end probe on coronal sections of control and Nurr1 cKO brains at P4. Nurr1+ CLA cells form a compact crescent-like structure in control brains but these cells over-migrate in InC and thus transform CLA into a wing-like structure in Nurr1 deficient brains. ML, midline. The dotted lines delineate the tissue borders.

(B) Quantification of Nurr1+ CLA neurons in the claustro-insular area of control and Nurr1 cKO brains at P2. The mean value of Nurr1+ CLA cells in control brains is ≈293.0 (n = 8), while the mean value of Nurr1-Cterm+ cells in cKO brains is ≈272.2 (n = 9; $p = 0.50$). The statistics for (**B**) was analyzed by two-sided *Student’s t-test*.

(C, D) IF for Nurr1-Cterm and cleaved-Caspase3 on E16.5 coronal sections and P2 sagittal sections of control and Nurr1 cKO brains shows only a few apoptotic cells in either control or cKO brains, suggesting that Nurr1 does not affect survival/apoptosis of CLA neurons. The arrowheads indicate cleaved-Caspase3 positive signals. Scale bar: 200 µm.

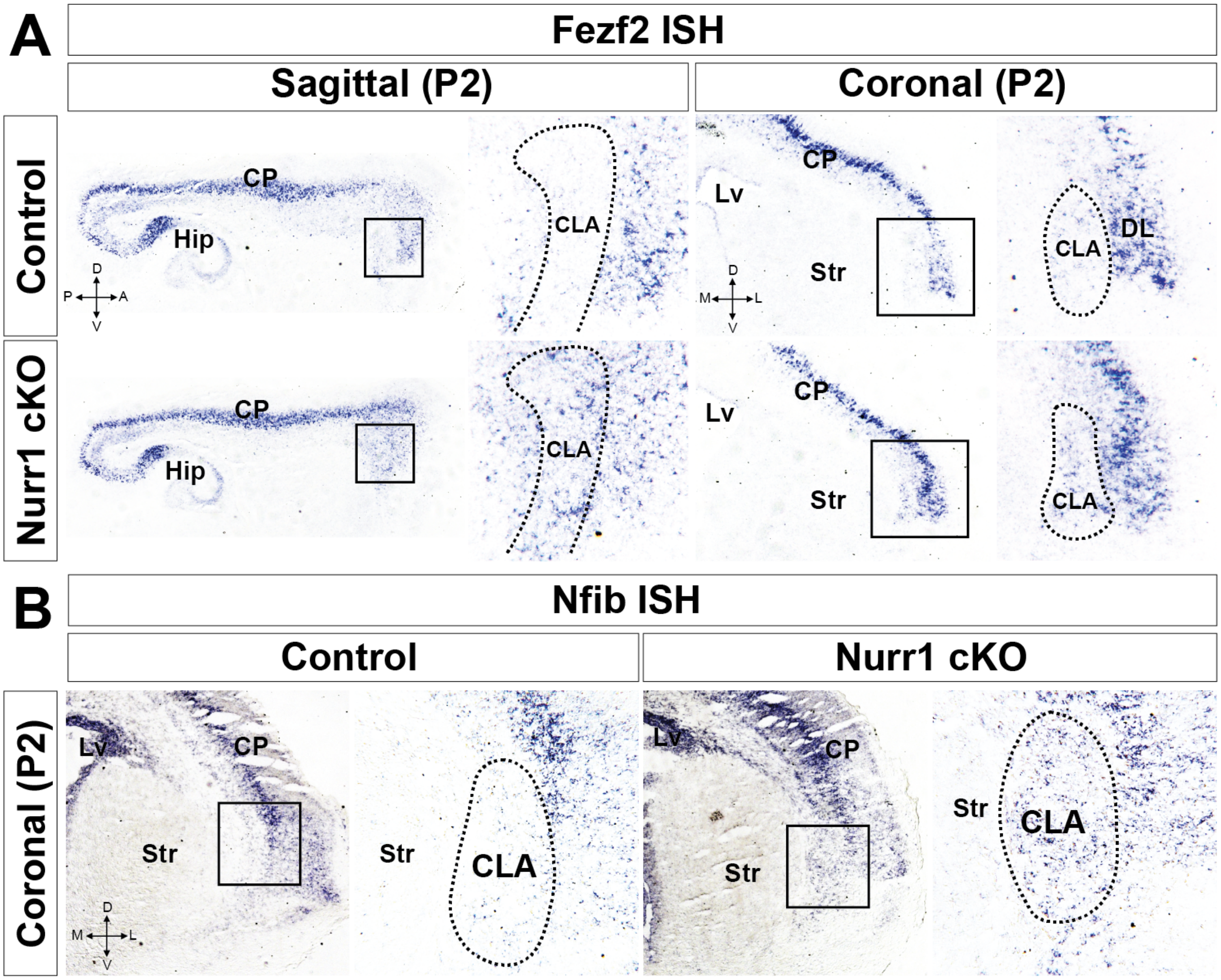

Figure S5 Fezf2 and Nfib expression is ectopically detectable in claustrum of Nurr1 deficient brains.

(A, B) ISH using the Fezf2 and Nfib probes on sagittal (**A**) and coronal (**B**) sections of control and Nurr1 deficient brains at P2. Fezf2 and Nfib expression is barely detected in CLA of normal brains but Fezf2+ and Nfib+ deeper layer (DL) neurons are populated in original CLA area of Nurr1 deficient brains.

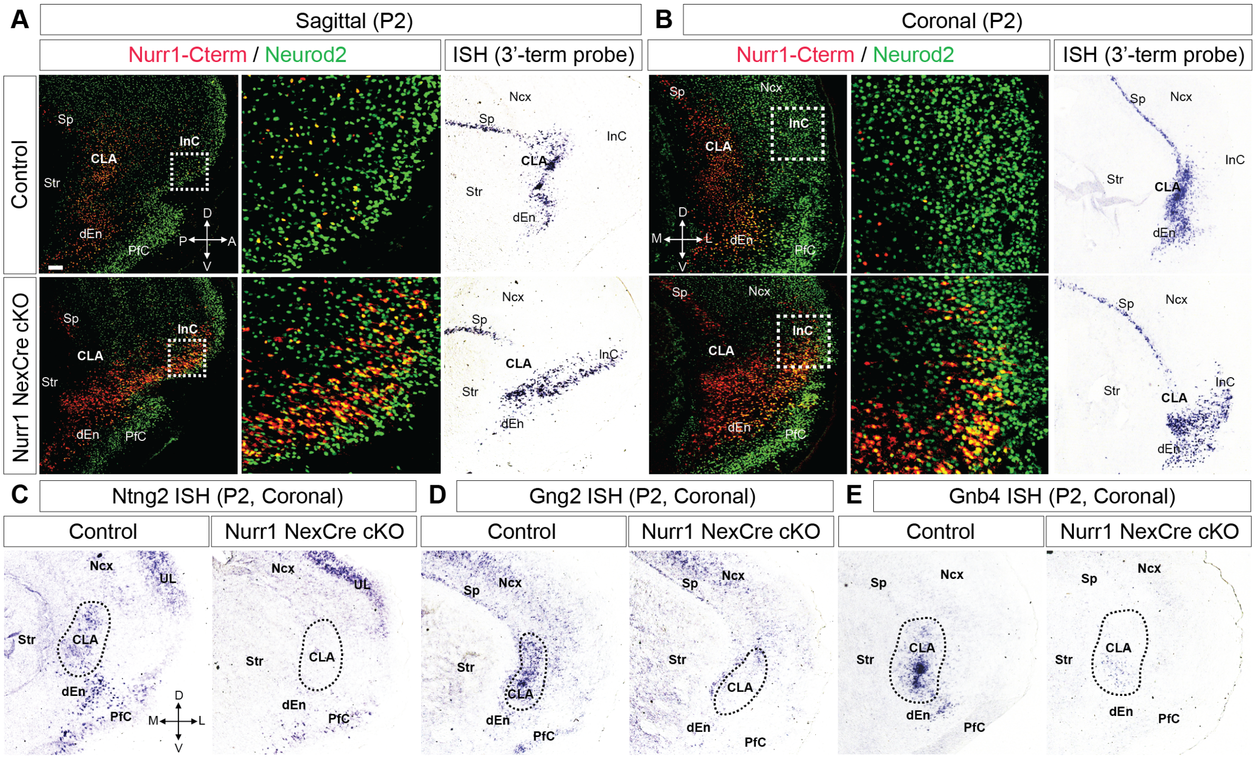

Figure S6 The Nex$^{Cre/Wt}$ Nurr1 deficient claustral neurons recapitulate the same phenotypes as those in Emx1$^{Cre/Wt}$ mutant brains.

(A, B) IF for Nurr1-Cterm and Neurod2 on sagittal and coronal sections of control and Nex$^{Cre/Wt}$ Nurr1 deficient brains at P2 shows that Nurr1+/Neurod2+ neurons aberrantly migrate into InC in Nex$^{Cre/Wt}$ mutant brains, mimicking the same phenotypes as in Emx1$^{Cre/Wt}$ mutant brains. ISH using the Nurr1 3'end probe verifies the same results consistent with IF staining. Scale bar: 200 µm.

(C-E) ISH using the Ntng2 (**C**), Gng2 (**D**) and Gnb4 (**E**) probes on coronal sections of control and Nex$^{Cre/Wt}$ mutant brains at P2 shows that the expression of these genes is considerably downregulated in CLA and/or dEn cells of Nex$^{Cre/Wt}$ mutant brains, similar with Emx1$^{Cre/Wt}$ mutant brains. UL, upper layers of Ncx.

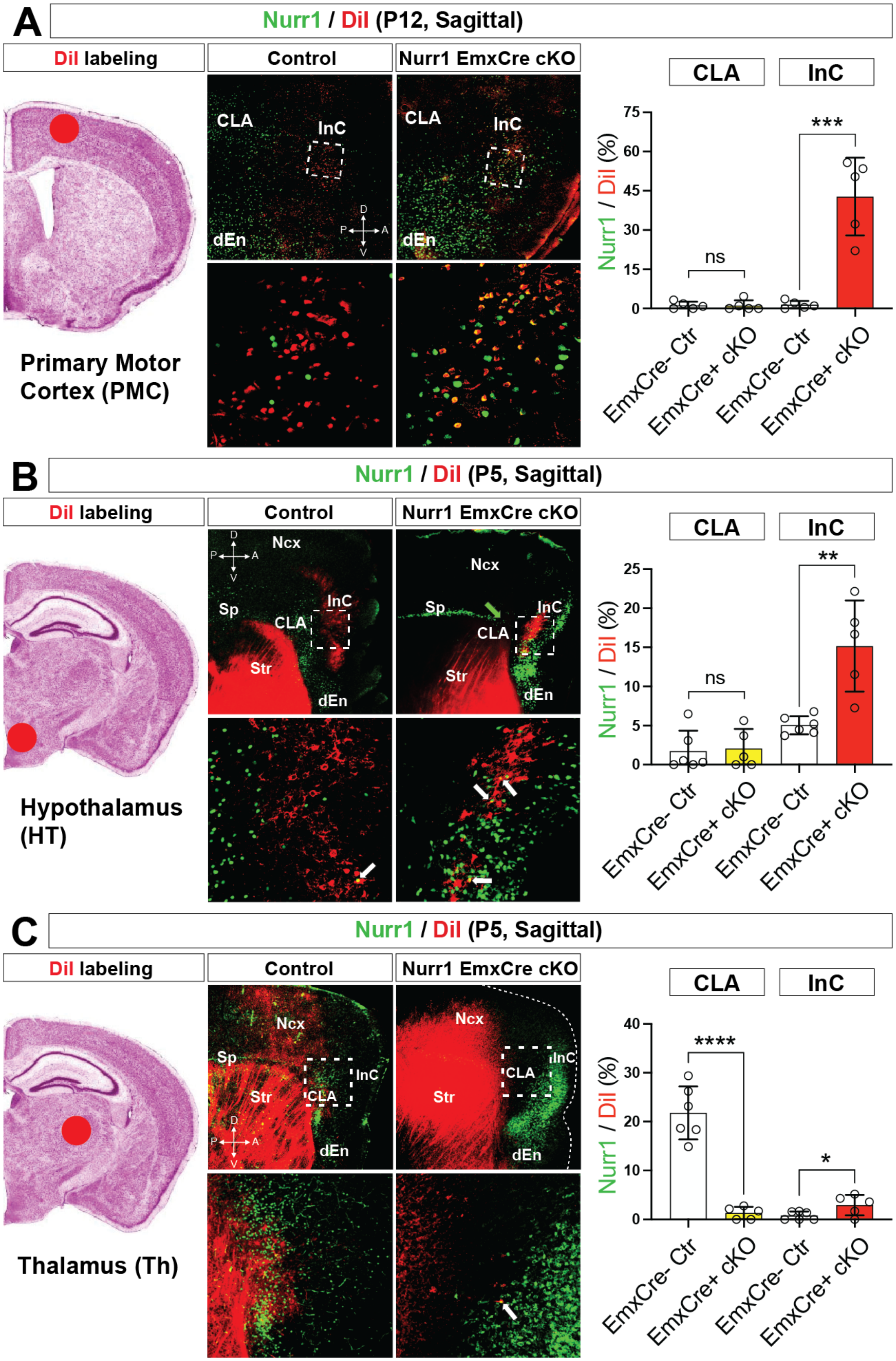

83
84 **Figure S7 Retrograde tracing analysis of claustral axon projection in Nurr1 deficient mice**

(A) DiI crystals were placed into primary motor cortex (PMC) at P12 for axonal retrograde tracing. IF for Nurr1-Cterm on brain sections carrying DiI signals shows that DiI+ cell bodies traced from axonal terminals in PMC were mostly found in InC, but seldom colocalized with Nurr1+ CLA cells in control or Nurr1 cKO brains. In contrast, Nurr1 deficient CLA cells (Nurr1-Cterm+) migrated into InC and were ectopically colocalized with DiI+ signals. The ratio of Nurr1+/DiI+ cells in the InC relative to all DiI+ cells was $\approx$1.45% in control brains ($n = 5$), but significantly elevated up to $\approx$42.81% (by ~28.52 $\pm$ 4.49 folds) in Nurr1 cKO brains ($n = 5$; $p = 0.00026$). The statistics for (**A–C**) was analyzed by two-sided *Student's t-test*.
(B) DiI crystals were buried into hypothalamus (HT) at P5 for axonal retrograde tracing. DiI+ somas traced from HT axonal terminals were also mainly found in the InC neighboring to CLA in control brains, but more Nurr1-Cterm+ cells were found colocalized with DiI+ signals in Nurr1 cKO brains. The green arrowhead indicates the CLA of a Nurr1 deficient brain, and white arrowheads indicate Nurr1+/DiI+ cells. The ratio of Nurr1+/DiI+ cells in the InC was $\approx$5.04% in control brains ($n = 6$), but elevated up to $\approx$15.16% in Nurr1 cKO brains ($n = 5$; $p = 0.0023$). The Nurr1+/DiI+ cells were barely observed in the CLA areas of control and Nurr1 cKO brains.
(C) DiI crystals were placed into thalamus at P5 for axonal retrograde tracing. DiI+ signals from thalamic nuclei were detectable in some of the Nurr1+ CLA neurons in control brains ($\approx$21.78%; $n = 6$), but Nurr1+/DiI+ cells were rarely detected in the CLA of Nurr1 cKO brains ($\approx$1.33%; $n = 5$) due to the over-migration of Nurr1-Cterm+ cells. DiI retrograde signals in the InC are few. The ratio of Nurr1+/DiI+ cells in the InC relative to all DiI+ cells was $\approx$0.78% in control brains ($n = 6$), but slightly increased to $\approx$2.94% in Nurr1 cKO brains ($n = 5$; $p = 0.044$).

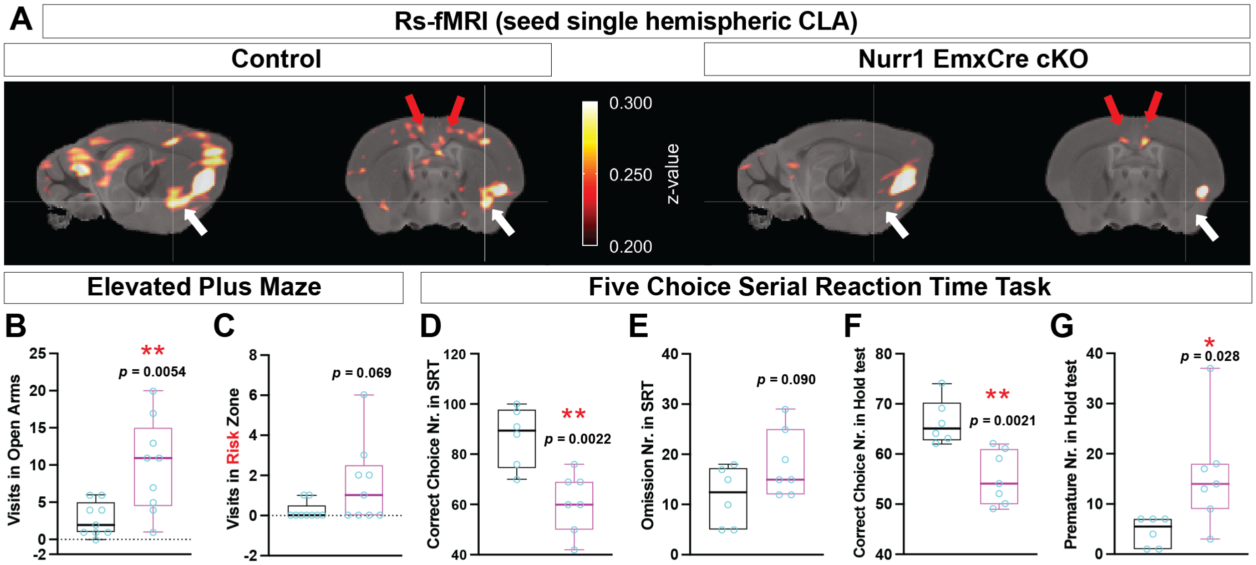

Figure S8 Nurr1 regulates claustral neuron connectivity and claustrum-relevant behaviors. **(A)** The synchronous BOLD signals of rs-fMRI in amygdala (white arrowheads) and anterior cingulate cortex (red arrowheads) networks were specifically weakened in Nurr1 cKO brains when single right hemispheric CLA was seeded as the region of interest in seed-based connectivity mapping approach.

(B, C) In elevated plus maze (EPM), Nurr1 deficient mice exhibited higher visit frequencies to the open arms (≈9.89) and risk zones (≈1.56; $p = 0.069$) than control mice (open arms: ≈2.78; risk zones: ≈0.22).

(D-G) In five choice serial reaction time task (5CSRTT), the number of correct nose-poking responses of Nurr1 cKO mice (≈60.86) was decreased by ≈30.05% ± 7.55% relative to that of control mice (≈87.00), whereas the omission number of Nurr1 cKO mice (≈18.14) was increased by ≈55.49% ± 29.88% ($p = 0.090$) than that of control mice (≈11.67) in the stimuli responsive tests. In the hold test of 5CSRTT, the number of correct nose-poking responses of Nurr1 cKO mice (≈55.29) was significantly decreased by ≈16.66% ± 4.18% than that of control mice (≈66.33), while the number of premature responses of Nurr1 cKO mice (≈15.86) was increased by ~2.52 ± 1.00 folds relative to that of control mice (≈4.50). The statistics for each behavior test parameter (**B - G**) was analyzed by two-sided *Student's t-test*.

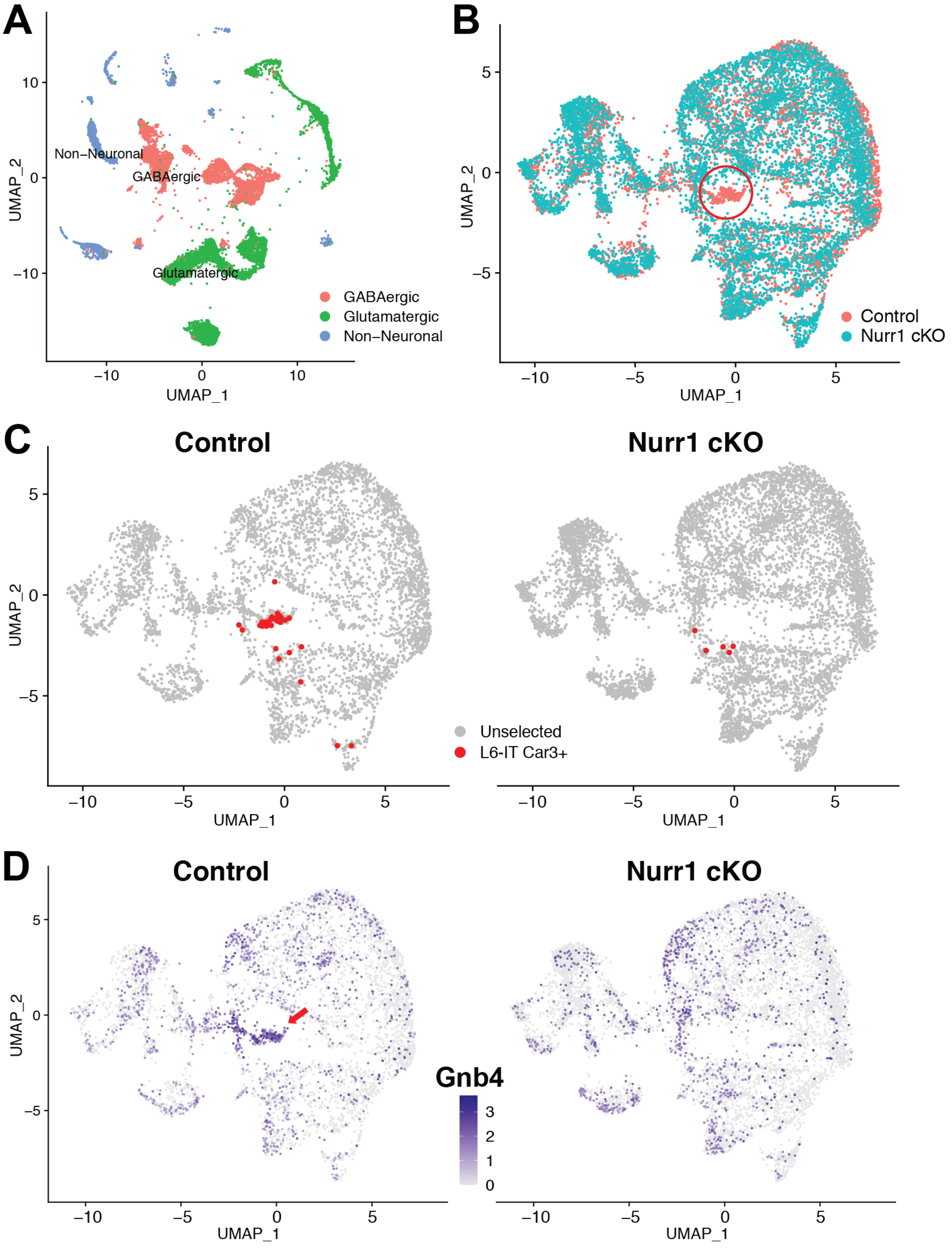

Figure S9 Nurr1 controls specific gene expression profile in claustral cells.

(A) Uniform manifold approximation and projection (UMAP) in single cell transcriptomes based on all cells isolated from claustro-insular tissues defines three main cell populations: glutamatergic neurons, GABAergic neurons and non-neuron populations.

(B) UMAP analysis of glutamatergic neurons populations in claustro-insular tissues of control and Nurr1 cKO brains by genotype. There is a subset of L6-IT (layer 6 intra-telencephalon projecting glutamatergic neurons) cluster missing in the Nurr1 cKO brains (red circle).

(C) UMAP of L6-IT Car3+ cell subpopulation in clastro-insular glutamatergic neurons shows that these cells are largely overlapping with Nurr1+ CLA cell populations in controls, but they disappear in Nurr1 cKO brains.

(D) UMAP of Gnb4 based on glutamatergic neurons shows that Gnb4 expression is specifically downregulated in the Nurr1+/Car3+ cells (mainly CLA cells) in Nurr1 cKO brains (red arrowhead). The expression level is marked from light blue (minimal) to dark blue (maximal).

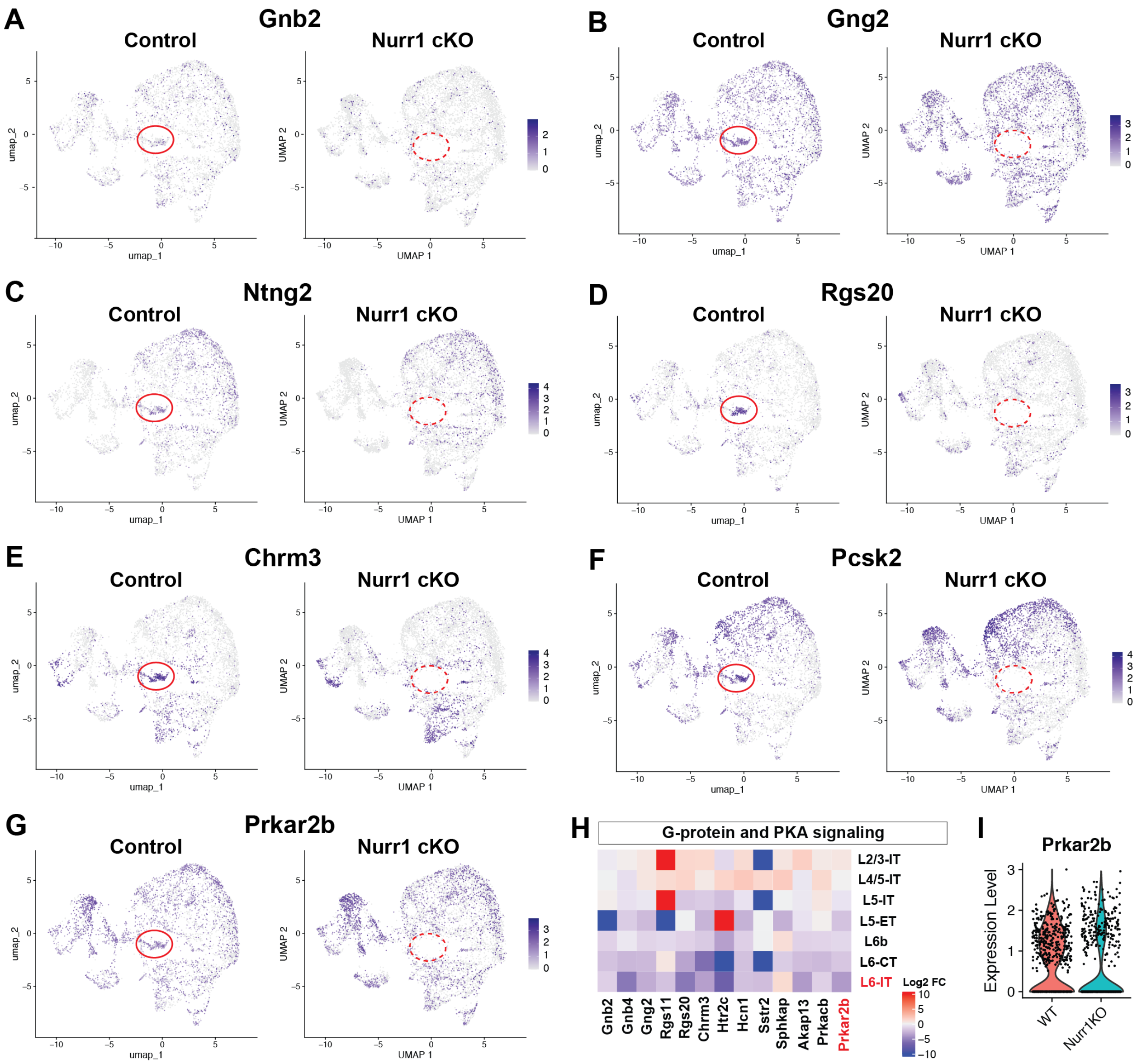

Figure S10 Nurr1 controls specific gene expression profile in claustral cells.
(A-G) UMAP of single cell transcriptomes for Gnb2, Gng2, Ntng2, Rgs20, Chrm3, Pcsk2 and Prkar2b based on clastro-insular glutamatergic neurons shows that these genes are specifically downregulated in Nurr1+ CLA/dEn cell populations of Nurr1 cKO brains. Nurr1 is predominately expressed in the L6-IT cluster containing CLA cells (red circles). The expression level bars are beside each UMAP.
(H) Cell type specific heatmap for the genes involved in G-protein or PKA signalling. These genes altered their expression in Nurr1 lineage cell populations of Nurr1 cKO brains. Log_2 fold changes (Log_2-FC) in expression levels of Nurr1 deficient brains relative to controls are marked by color bars. L2/3- or L4/5-IT, layer 2 and 3 or layer 4 and 5 intra-telencephalon projecting glutamatergic neurons; L5-ET, layer 5 extra-telencephalon projecting glutamatergic neurons; L6b, layer 6b neurons (Sp), L6-CT, layer 6 corticothalamic projecting neurons.
(I) Violin plot for Prkar2b in Nurr1+ glutamatergic neurons in claustro-insular tissues indicates the expression level of Prkar2b is downregulated in Nurr1-Cterm+ neurons of Nurr1 cKO brains.

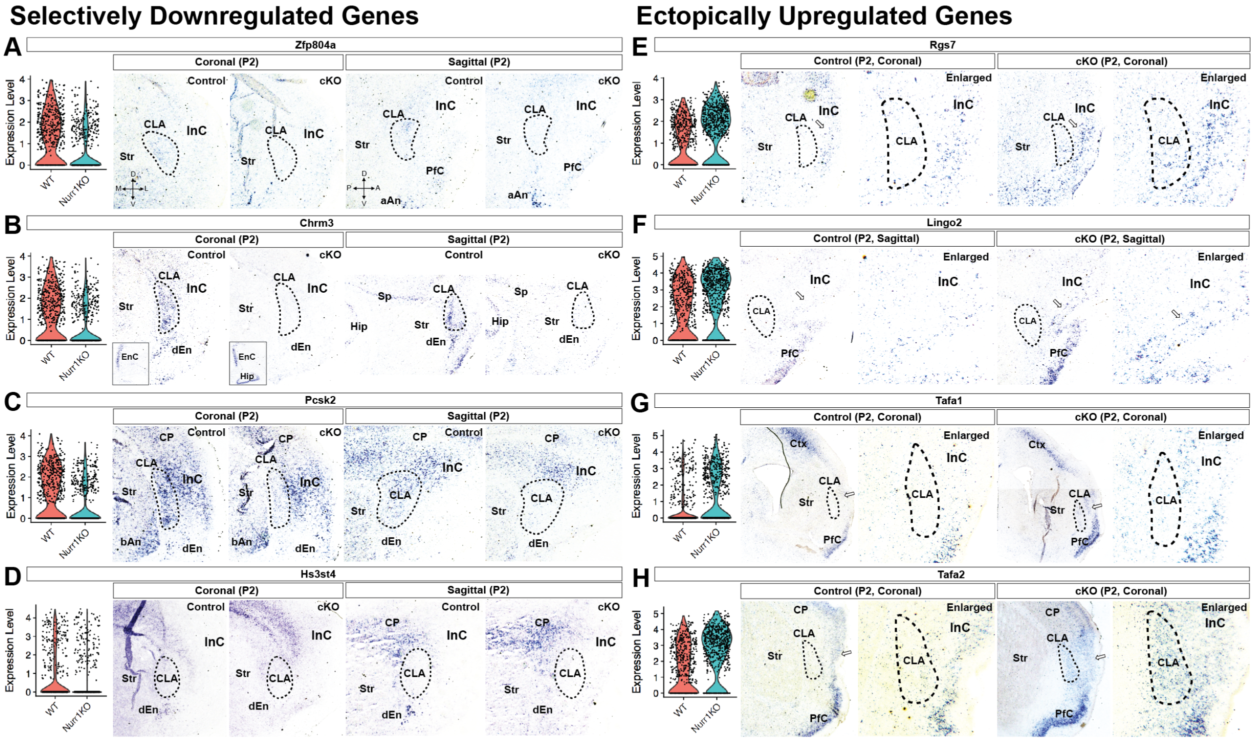

Figure S11 ISH verified the misregulated gene expression in transcriptomic analysis.
(A-D) ISH verification of downregulated genes in EmxCre+ Nurr1 deficient CLA and/or dEn cells. ISH using the Zfp804a (**A**), Chrm3 (**B**), Pcsk2 (**C**) and Hs3st4 (**D**) probes on coronal and sagittal sections of control and Emx1$^{Cre/Wt}$ mutant brains at P2. The violin plots of target genes (**A-H**) resulted from single cell mRNA sequencing are seated left to the ISH data. Zfp8104a expression is downregulated in Nurr1 deficient CLA cells but not in anterior amygdalar nuclei (aAn). Chrm3 expression is selectively downregulated in Nurr1 deficient CLA and dEn cells but not in entorhinal cortex (EnC) or Hip. Pcsk2 expression is downregulated in Nurr1 deficient CLA cells but not in basomedial amygdalar nuclei (bAn) and surrounding cortical neurons. Hs3st4 expression is selectively reduced in Nurr1 deficient dEn cells but not in surrounding DL neurons.
(E-H) ISH verification of ectopically upregulated genes in *Emx1*$^{Cre/Wt}$ Nurr1 deficient CLA cells. ISH using the Rgs7 (**E**), Lingo2 (**F**), Tafa1 (**G**) and Tafa2 (**H**) probes on coronal or sagittal sections of control and Nurr1 deficient brains at P2. The expression of Rgs7, Lingo2, Tafa1 and Tafa2 is barely detectable in CLA cells of control brains, however, their expression is upregulated in neighboring neocortex in Nurr1 deficient brains. The magnification views of CLA and InC are presented in the enlarged images.

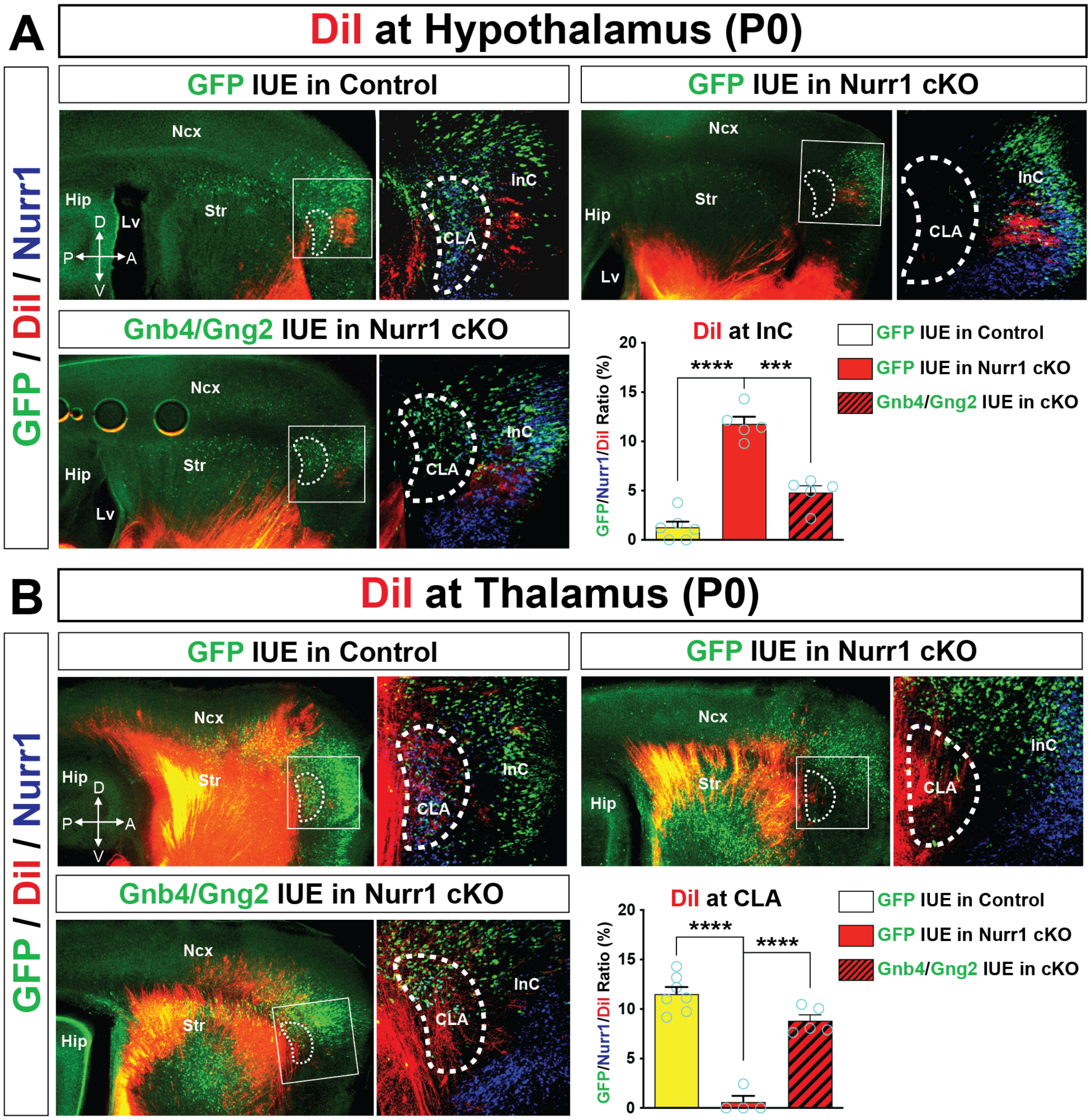

Figure S12 Co-electroporation of Gβ/γ subunits rescued the axonal misrouting of Nurr1 deficient claustral neurons.

(A) IF for Nurr1-Cterm and GFP in combination with DiI+ dye signals traced from hypothalamus (**A-C**) and quantification of the ratios of Nurr1-Cterm/GFP/DiI triple positive cells in the InC relative to all DiI+ cells in the claustro-insular cortical areas (**D**). This ratio was very low in GFP-electroporated control brains (≈1.30%; n = 6), but was elevated to ≈11.76% in GFP-electroporated Nurr1 cKO brains (n = 5; $p < 0.0001$). This ratio was reduced to ≈4.81% when Gnb4/Gng2 (with bicistronic GFP) were co-electroporated into Nurr1 cKO brains as compared to GFP IUE (n = 5; $p = 0.00013$). The magnification images in (**A-C** and **E-G**) depict the framed claustro-insular areas in each condition. The statistics for (**A, B**) was analyzed by two-sided *Student's t-test*.

(B) IF for Nurr1-Cterm and GFP with DiI+ signals traced from thalamus (**E-G**) and the ratios of Nurr1-Cterm/GFP/DiI triple positive cells in the CLA relative to all DiI+ cells (**H**). The ratio in GFP-electroporated control brains was ≈11.51% (n = 7) while the ratio in GFP-electroporated Nurr1 cKO brains was ≈0.61% (n = 4; $p < 0.0001$). In contrast, this ratio was increased to ≈8.82% in the Nurr1 cKO brains carrying co-IUE of Gnb4/Gng2 (n = 5; $p < 0.0001$).

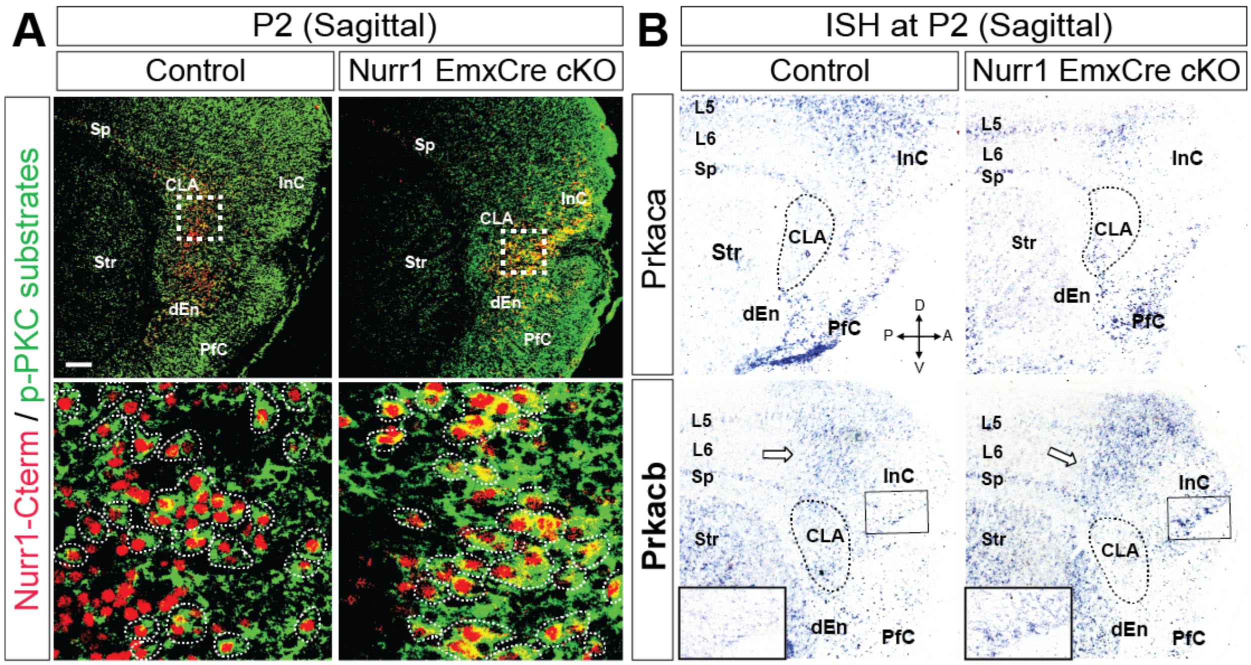

Figure S13 One of PKA catalytic subunits, Prkacb, is ectopically upregulated in insular cortex.

(A) IF for Nurr1-Cterm and phosphorylated substrates of PKC on sagittal sections of control and Nurr1 cKO brains at P2 shows that PKC was active in most Nurr1 lineage cells of both control and Nurr1 cKO brains. Each dotted line circle indicates one or one cluster of double positive cells. Scale bar: 200 µm.

(B) ISH using the Prkaca and Prkacb probes on sagittal sections of control and Nurr1 cKO brains at P2 shows that the expression of Prkacb, one of the PKA catalytic subunit, is ectopically activated in the InC of Nurr1 cKO brains. The hollow arrowheads indicate the borderlines of Prkacb positive signals, highly consistent with IF staining in Figure 6D.

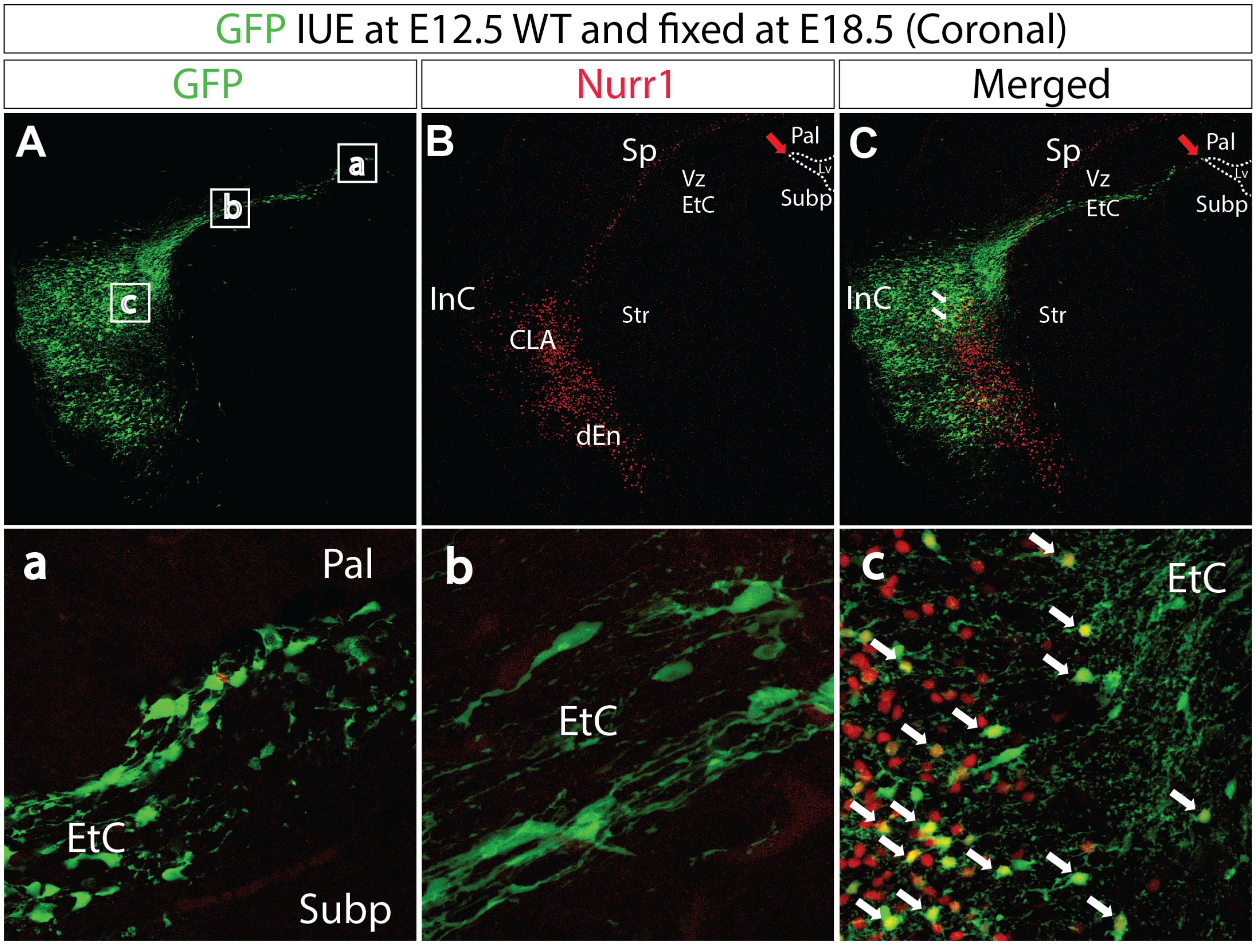

Figure S14 GFP electroporation at E12.5 in the progenitors at pallial-subpallial border targets the claustral cells.

(A-C) In utero electroporation (IUE) of GFP into wild type brains at E12.5 targeting neural progenitors at the VZ of pallial-subpallial (Pal-subp) border illustrates a population of neurons migrate in the external capsule (EtC) towards lateral pallium. These cells later leave EtC, of which a major subpopulation become Nurr1+ CLA cells (yellow). Vz, ventricular zone. The dotted line circles indicate the CLA. The red arrowheads indicate the Pal-subp border.

(a-c) The enlarged views of framed areas in (**A**). These immature cells originate from Pal-subp border (**a**) and migrated in EtC (**b**). CLA cells start to express Nurr1 intermediately after they leave EtC and reside at CLA (**c**). Each white arrowhead indicates one GFP+/Nurr1+ cell.

Table S1 Nucleus numbers and proportions in Nurr1 lineage cells of transcriptomic analysis

Genotype	L2/3-IT	L4/5-IT	L5-IT	L5-ET	L6-IT	L6-CT	L6b	Total
Control	41 (7.59%)	76 (14.07%)	28 (5.19%)	11 (2.04%)	258 (47.78%)	69 (12.78%)	57 (10.56%)	540
Nurr1 cKO	79 (12.40%)	232 (36.42%)	47 (7.38%)	6 (0.94%)	151 (23.70%)	43 (6.75%)	79 (12.40%)	637
Change	63.37% increase	158.85% increase	42.20% increase		50.40% decrease	47.18% decrease	16.86% increase	

Note: 1 and 2 cells in the L5-NP clusters of control and Nurr1 deficient brains are excluded from counting, respectively.

Table S2 Oligos and Primers

**Name:**	**Usage:**	**Sequence: 5' – 3'**
Nurr1-Nterm-Fw	Nurr1 ISH for 5'end	TCCTCGCCTCAAGGAGCCAGCCCCG
Nurr1-Nterm-Rv		AAGTGCGAACACCGTAGTGCTGACA
Nurr1-Cterm-Fw	Nurr1 ISH for 3'end	GTTAAAGAAGTGGTTCGCACGGACA
Nurr1-Cterm-Rv		CTTAGAAAGGTAAGGTGTCCAGGAAA
Cux2-Fw	Cux2 ISH	GATGGAGACAGCCAGCCCCAGG
Cux2-Rv		TTCAGAATTCCCACTCCAGGAC
Cdh13-Fw	Cdh13 ISH	TCGCTACTTATCAACTGTATGTGGA
Cdh13-Rv		TGGGTCCTTGTAGATAGAGTACCTG
Fezf2-Fw	Fezf2 ISH	TCATGTGATGTCAGCTGAATGTAAA
Fezf2-Rv		TGGAGTCCAGGTAGTTGAAGTAGTA
Nfib-Fw	Nfib ISH	TCAATGTATCAGAGCTTGTGAGAGT
Nfib-Rv		AAGGGAATTAGTGACTGTAAGTGCT
NtnG2-Fw	NetrinG2 ISH	GAAGGATTATGTCAAGGTCAAAGTG
NtnG2-Rv		CGATATTGGAGATGGCATAGAAGTA
Gnb4-Fw	Gnb4 ISH	ATATACAACCTAAAGACCCGAGAGG
Gnb4-Rv		GAGAACAGAAAATATGGCACTCAAT
Gng2-Fw	Gng2 ISH	GGGGAAGCTGCTCTCTAACCAAGCC
Gng2-Rv		GACAGCTTATCAGAGGGTATTTGAA
Rgs20-Fw	Rgs20 ISH	AGTAGGAACCGCTCTGACTAGTGTA
Rgs20-Rv		CTGTTTTCTCAGCTAAGGACGTAAG
CTGF-Fw	CTGF (Ccn2) ISH	AGTTACCAATGACAATACCTTCTGC
CTGF-Rv		CCACGGTAGTTAAAAACACAGATTT
S100a10-Fw	S100a10 ISH	ATGATGCTTACGTTTCACAGGTTTG
S100a10-Rv		CCATTGGATTAAGTTTTCTCTCTCA
Zbtb20-Fw	Zbtb20 ISH	GACACATTCACTGACAAACTCTCAC
Zbtb20-Rv		AGTCATAGTCATCTTCCATTTCCTG
Prkaca-Fw	Prkaca ISH	GAAGATCTTAGACAAGCAGAAGGTG
Prkaca-Rv		ATAGTCGTCAAAGTTACTCGTGTCC

Prkacb-Fw	Prkacb ISH	TGGATTGCTATTTATCAGAGAAAGG
Prkacb-Rv		CTTACTATCTCACGGAGTGAAGAGC
Nurr1-Fw-XhoI	Generation of Nurr1 expression construct	TTTTTCTCGAGAGCCATGCCTTGTGTTCAGGCGCAGTAT
Nurr1-Rv-NotI		TTTTTGCGGCCGCTTAGAAAGGTAAGGTGTCCAGGAA
Gnb4-Fw-XhoI	Generation of Gnb4 expression construct	TTTTTCTCGAGGATGAGCGAGCTGGAGCAGCTGAGG
Gnb4-Rv-NotI		TTTTTGCGGCCGCTCAATTCCAGATTCTAAGAAAACT
Gng2-Fw-XhoI	Generation of Gng2 expression construct	TTTTTCTCGAGACCATGGCCAGCAACAACACCGCCA
Gng2-Rv-NotI		TTTTTGCGGCCGCTTAAAGGATGGCGCAGAAGAACT
Tafa1-Fw	Tafa1 ISH	CCTAAAATCCATTATCCCTGTCTTT
Tafa1-Rv		TGCTGTAACTAAGAAATGAGTGCTG
Tafa2-Fw	Tafa2 ISH	TAACCCATTAACCACTCCTAAATCA
Tafa2-Rv		ACAACATGTAGGCTTTCAGAGTTTC
Zfp804a-Fw	Zfp804a ISH	AAGAAACAATGAACACAACAGTGAA
Zfp804a-Rv		GTTTGCTCTGAGTTCTTCTCGATAC
Chrm3-Fw	Chrm3 ISH	CCTTGTAGAAAAGGGGTTTATCAAT
Chrm3-Rv		ATTTGTATGAAACTTGAACTGCACA
Hs3st4-Fw	Hs3st4 ISH	CTCTTCATGTGCACCCTGTC
Hs3st4-Rv		GGGTCAAGAAAGGAGGGACA
Pcsk2-Fw	Pcsk2 ISH	GTGACTCGACCTTTATTTCTGTCAT
Pcsk2-Rv		TCATAACAGGGAACTCTAAAACCAC
Lingo2-Fw	Lingo2 ISH	GTATACCTGACCCACCTTAACCTCT
Lingo2-Rv		TTTTAAGGTCCAGGGAGAAAGTATT
Rgs7-Fw	Rgs7 ISH	AGATTGATCATCCTTGTATCTGAGC
Rgs7-Rv		TCTCTTCTTGTCCACTTTAGCTTGT
Nurr1-GT-Fw	Nurr1 floxed genotyping	GCTGGAGCCAGAGTTGGAAG
Nurr1-GT-Rv		ATTCCTTGGAGACCTTCTCT
EmxCre-PrimerA	EmxCre genotyping	TCGATGCAACGAGTGATGAG
EmxCre-PrimerB		TTCGGCTATACGTAACAGGG
EmxCre-PrimerC		AAGGTGTGGTTCCAGAATCG
EmxCre-PrimerD		CTCTCCACCAGAAGGCTGAG
NexCre-PrimerA	NexCre genotyping	CCGCATAACCAGTGAAACAG
NexCre-PrimerB		GAGTCCTGGAATCAGTCTTTTTC
NexCre-PrimerC		AGAATGTGGAGTAGGGTGAC

ISH, *in situ* hybridization.

Table S3 Primary antibodies

Primary Antibody	Species	Company	Cat #	Dilution	Usage	RRID
Nurr1 N terminal	Mouse	Santa Cruz	sc-376984	1:300	IF	AB_2893391
Nurr1 C terminal	Goat	R&D systems	AF2156	1:500	IF	AB_2153894
Nestin	Mouse	Merck	MAB353	1:800	IF	AB_94911
Zbtb20	Rabbit	SIGMA	HPA016815	1:300	IF	AB_1858947
GFP	Rabbit	ThermoFisher	A-11122	1:500	IF	AB_2307355
GFP	Chicken	Abcam	Ab13970	1:1000	IF	AB_300798
Tle4	Mouse	Santa Cruz	sc-365406	1:500	IF	AB_10841582
Darpp32	Rabbit	Abcam	ab40801	1:500	IF	AB_731843
Cleaved Caspase 3	Rabbit	Cell Signaling	9579	1:500	IF	AB_10897512
Cytochrome P450 26b1 (Cyp26b1)	Mouse	Merck	MABS497	1: 500	IF	unidentified
Neurod1	Rabbit	Proteintech	12081-1-AP	1:1500	IF	AB_2877823
Rorβ	Mouse	R&D systems	PP-N7927-00	1:500	IF	AB_1964364
Phospho-(Ser/Thr) PKA substrates	Rabbit	Cell Signaling	9621	1:800	IF	AB_330304
Phospho-PKC substrates	Rabbit	Cell Signaling	6967	1:800	IF	AB_10949977

IF, immunofluorescence.
