# Supplementary figures and images for: Nurr1 Orchestrates Claustrum Development and Functionality
Source: Adv Sci (Weinh). 2025 Dec 22;13(13):e08999. doi: 10.1002/advs.202508999 (PMC12955989; doi:10.1002/advs.202508999)

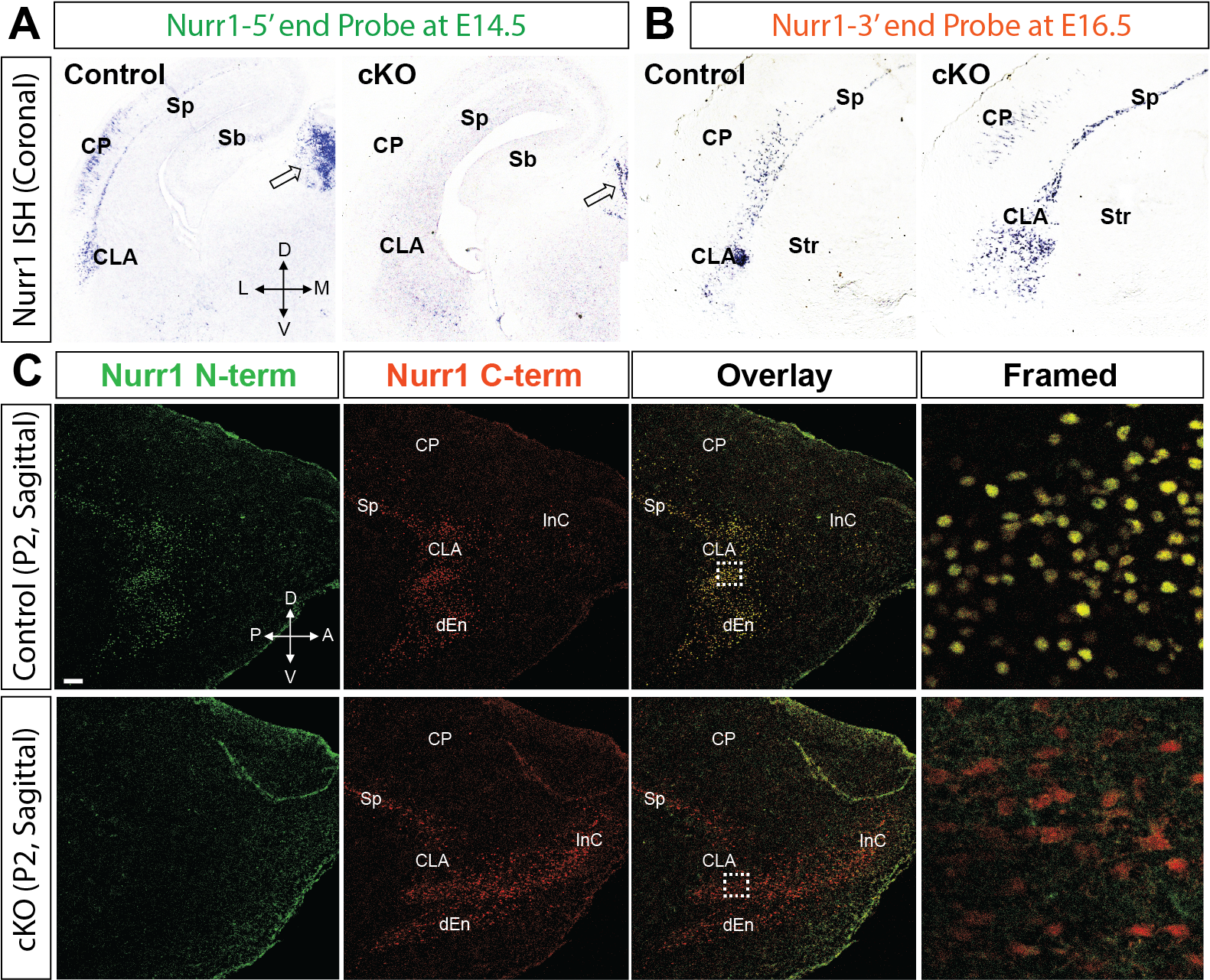

Supplement: Supplementary file 2 — Supporting File 2: advs73465‐sup‐0002‐Figure S1.png. [file ADVS-13-e08999-s017.png]

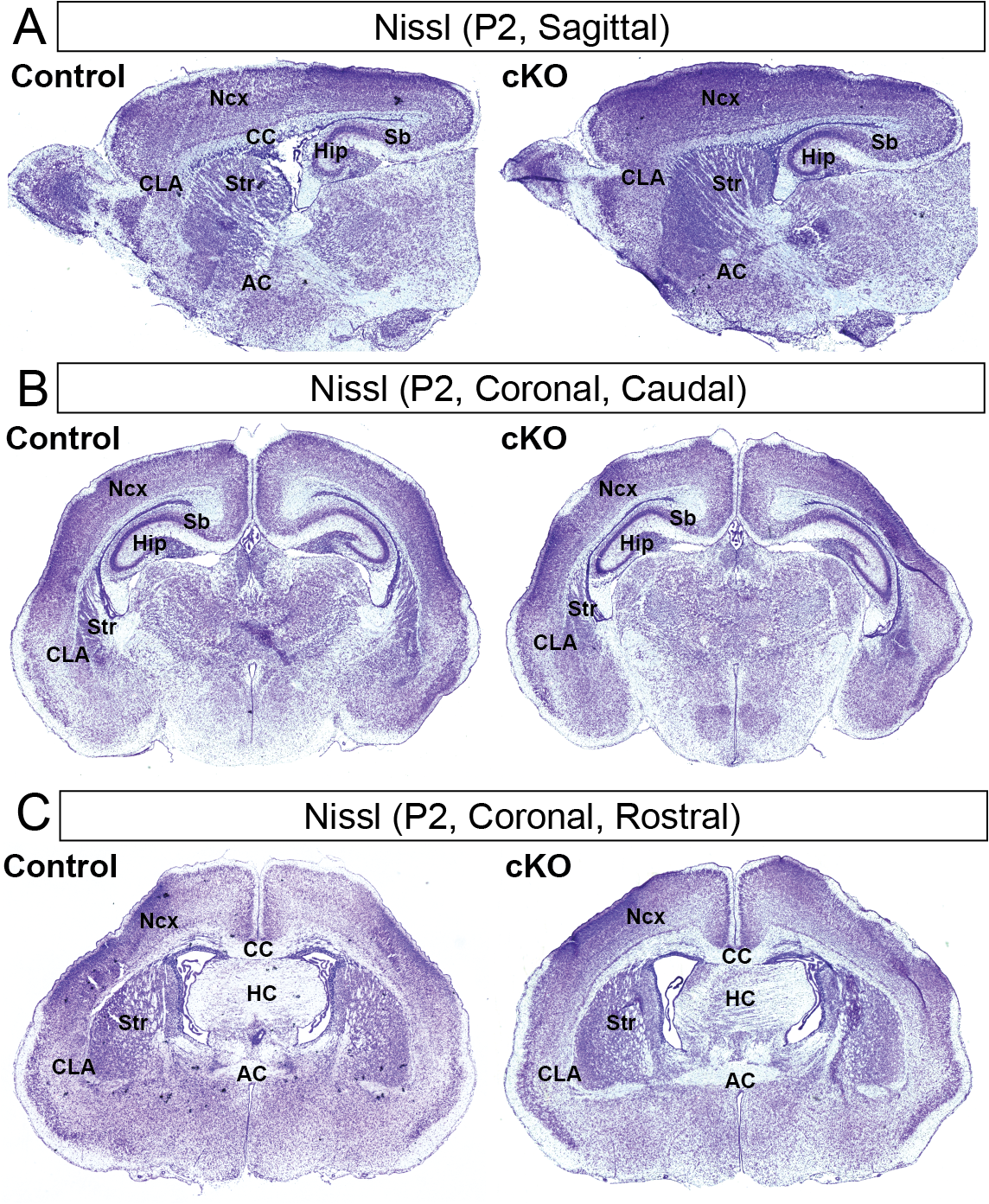

Supplement: Supplementary file 3 — Supporting File 3: advs73465‐sup‐0003‐Figure S2.png. [file ADVS-13-e08999-s001.png]

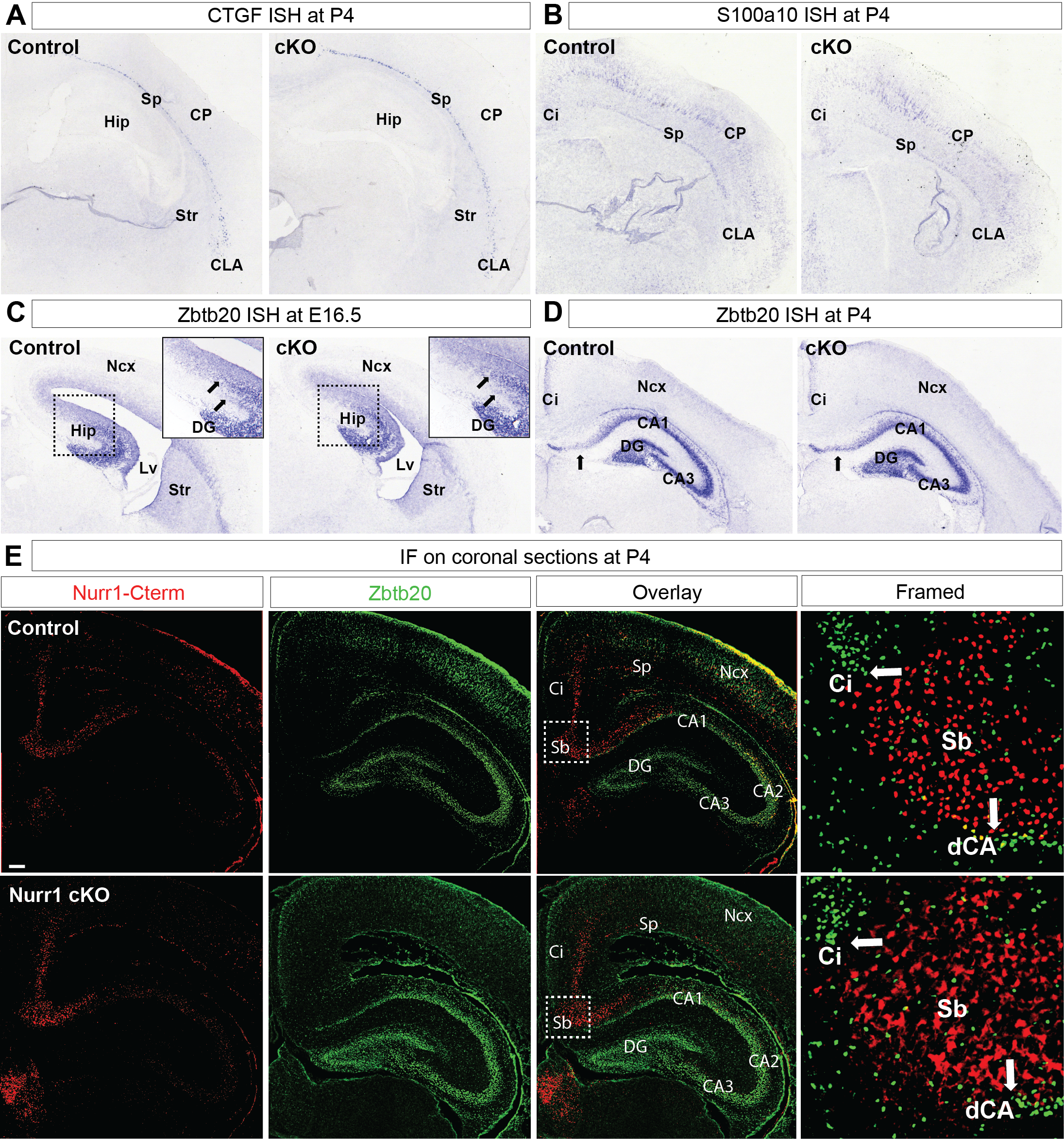

Supplement: Supplementary file 4 — Supporting File 4: advs73465‐sup‐0004‐Figure S3.png. [file ADVS-13-e08999-s006.png]

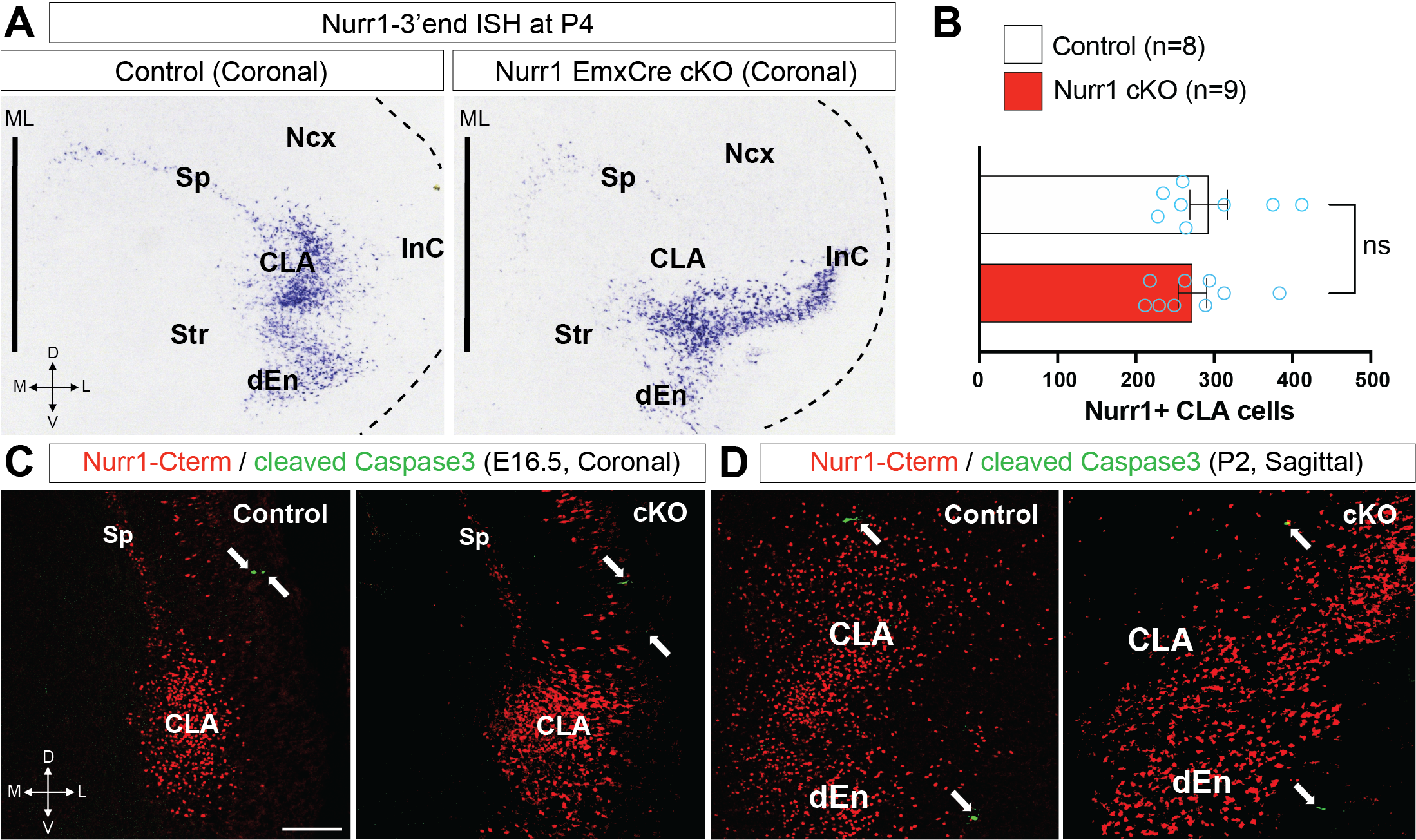

Supplement: Supplementary file 5 — Supporting File 5: advs73465‐sup‐0005‐Figure S4.png. [file ADVS-13-e08999-s005.png]

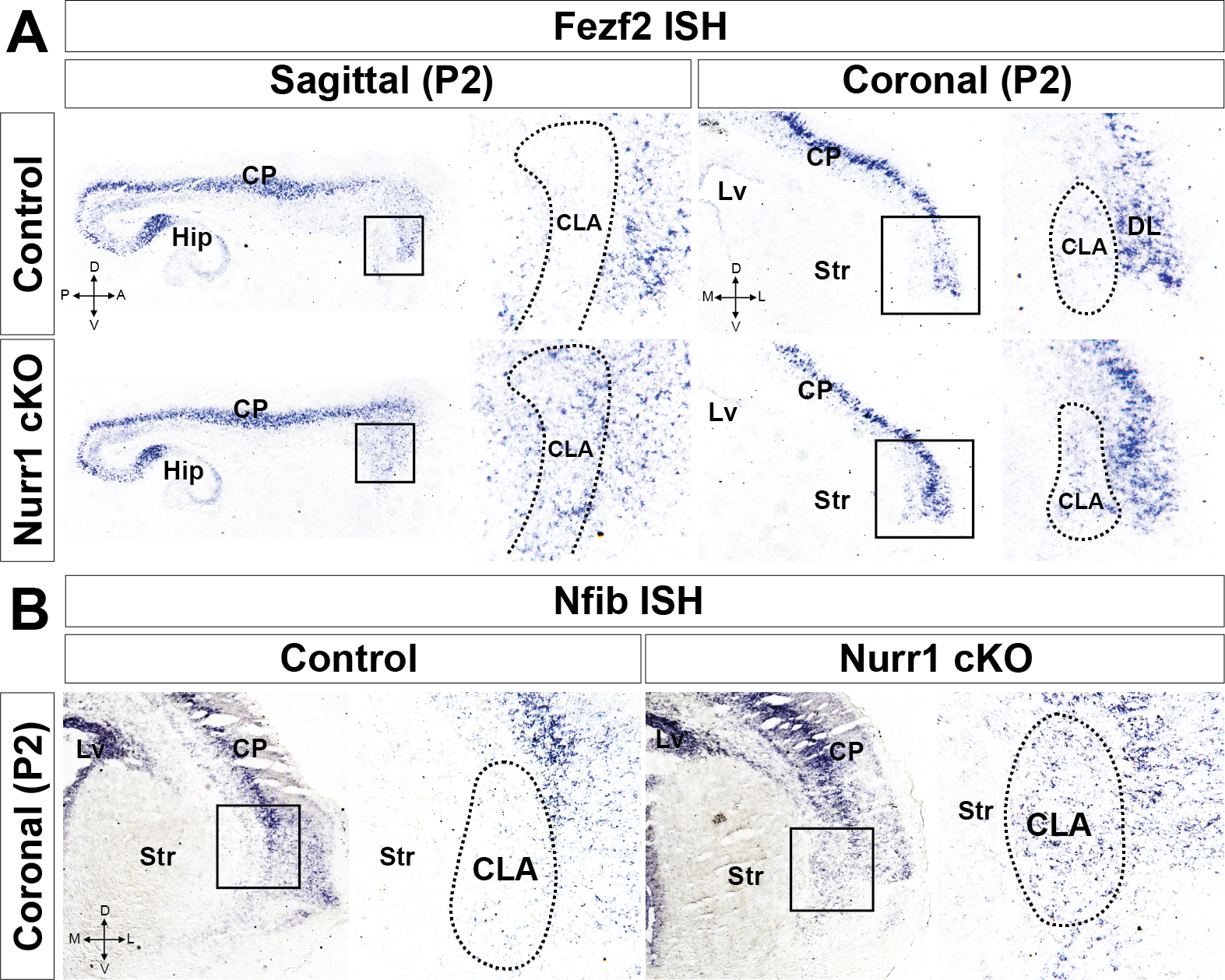

Supplement: Supplementary file 6 — Supporting File 6: advs73465‐sup‐0006‐Figure S5.png. [file ADVS-13-e08999-s002.png]

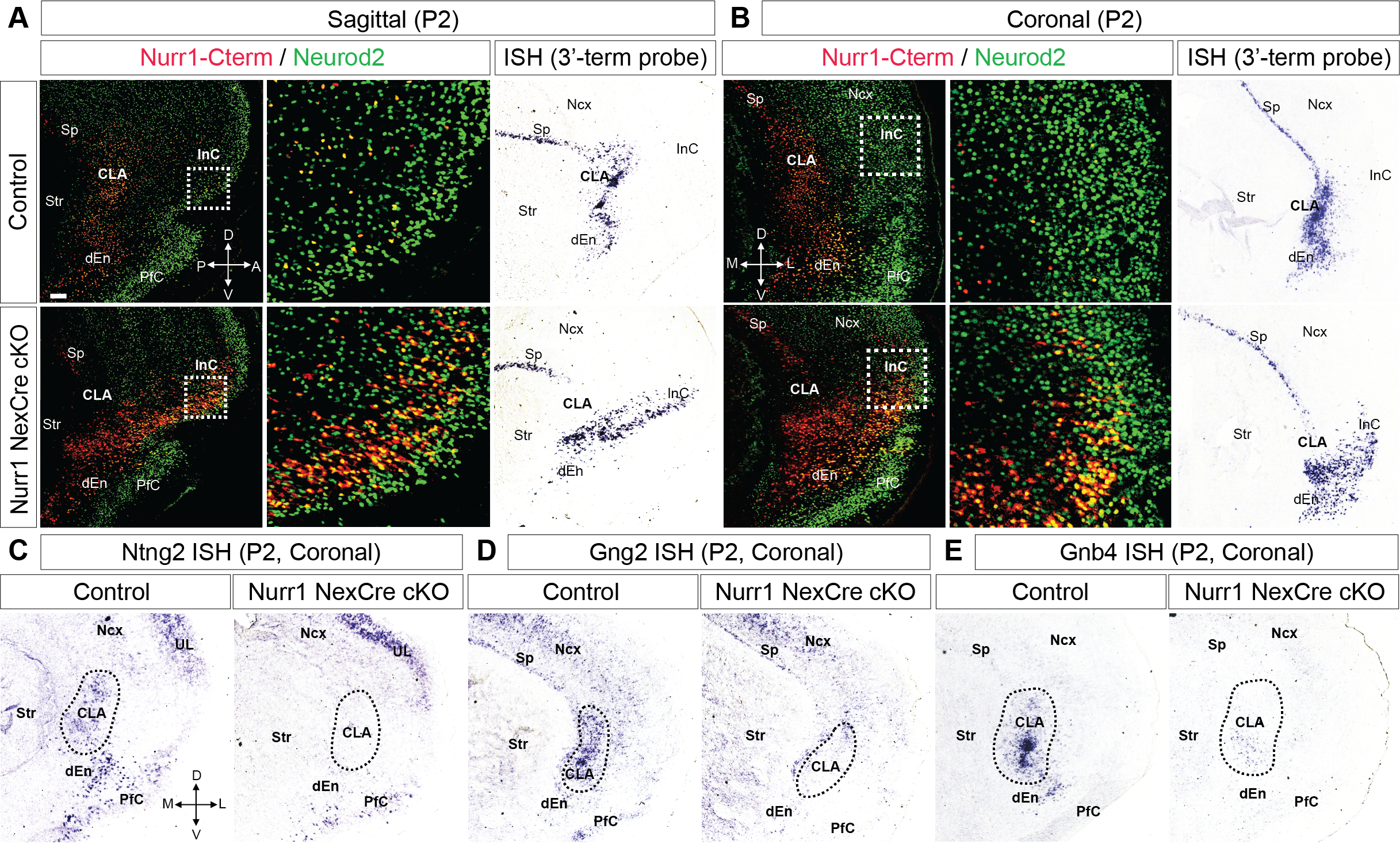

Supplement: Supplementary file 7 — Supporting File 7: advs73465‐sup‐0007‐Figure S6.png. [file ADVS-13-e08999-s013.png]

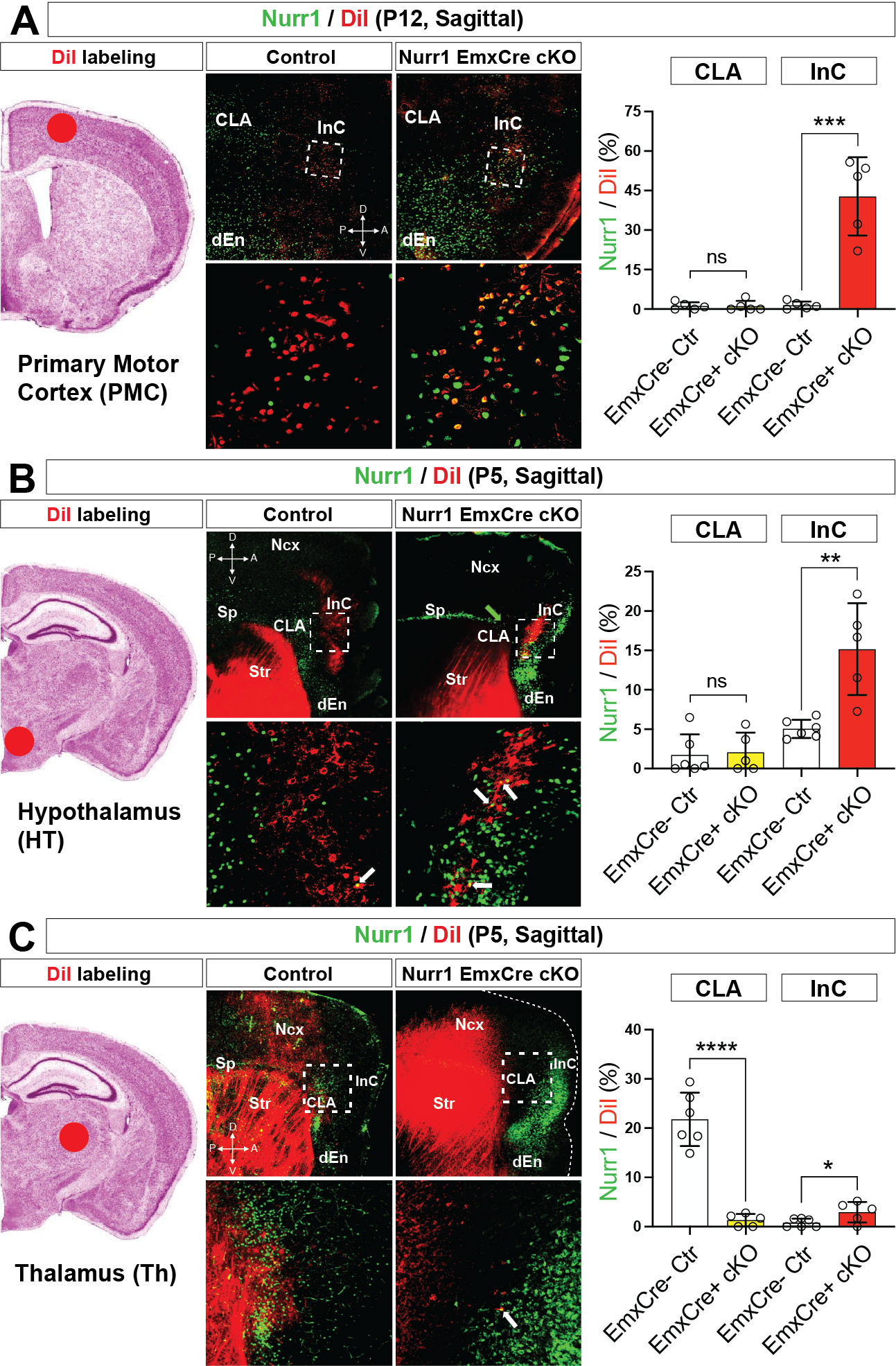

Supplement: Supplementary file 8 — Supporting File 8: advs73465‐sup‐0008‐Figure S7.png. [file ADVS-13-e08999-s010.png]

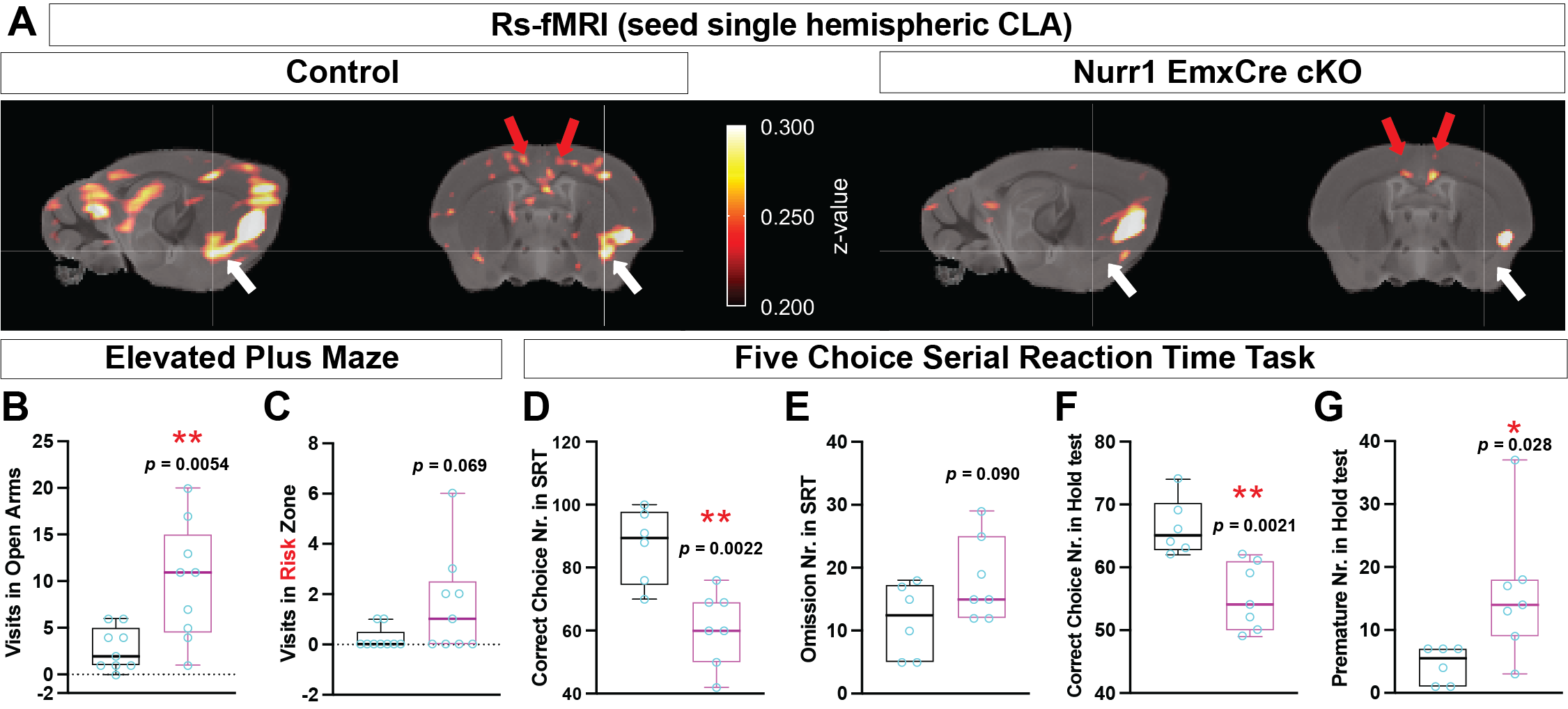

Supplement: Supplementary file 9 — Supporting File 9: advs73465‐sup‐0009‐Figure S8.png. [file ADVS-13-e08999-s011.png]

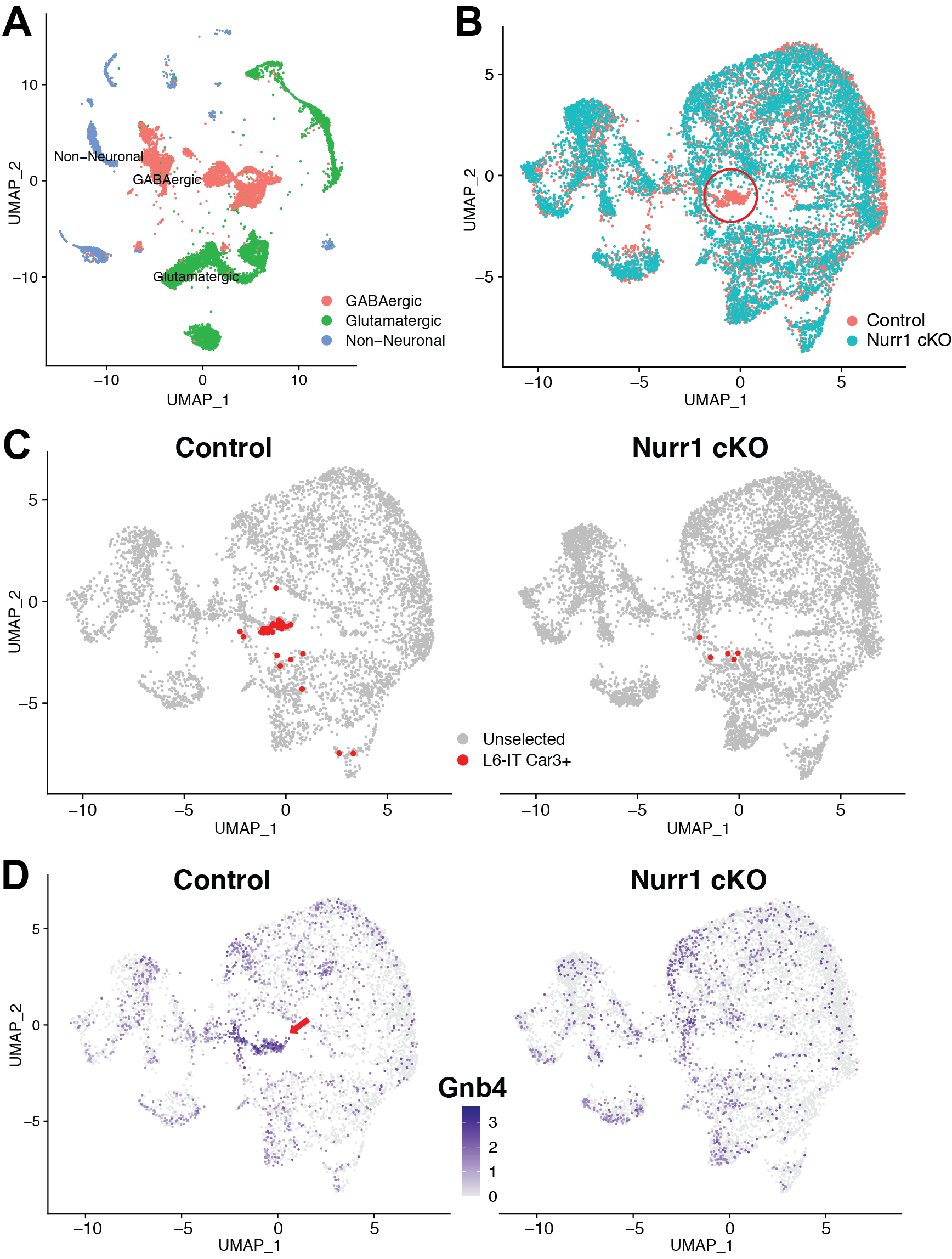

Supplement: Supplementary file 10 — Supporting File 10: advs73465‐sup‐0010‐Figure S9.png. [file ADVS-13-e08999-s008.png]

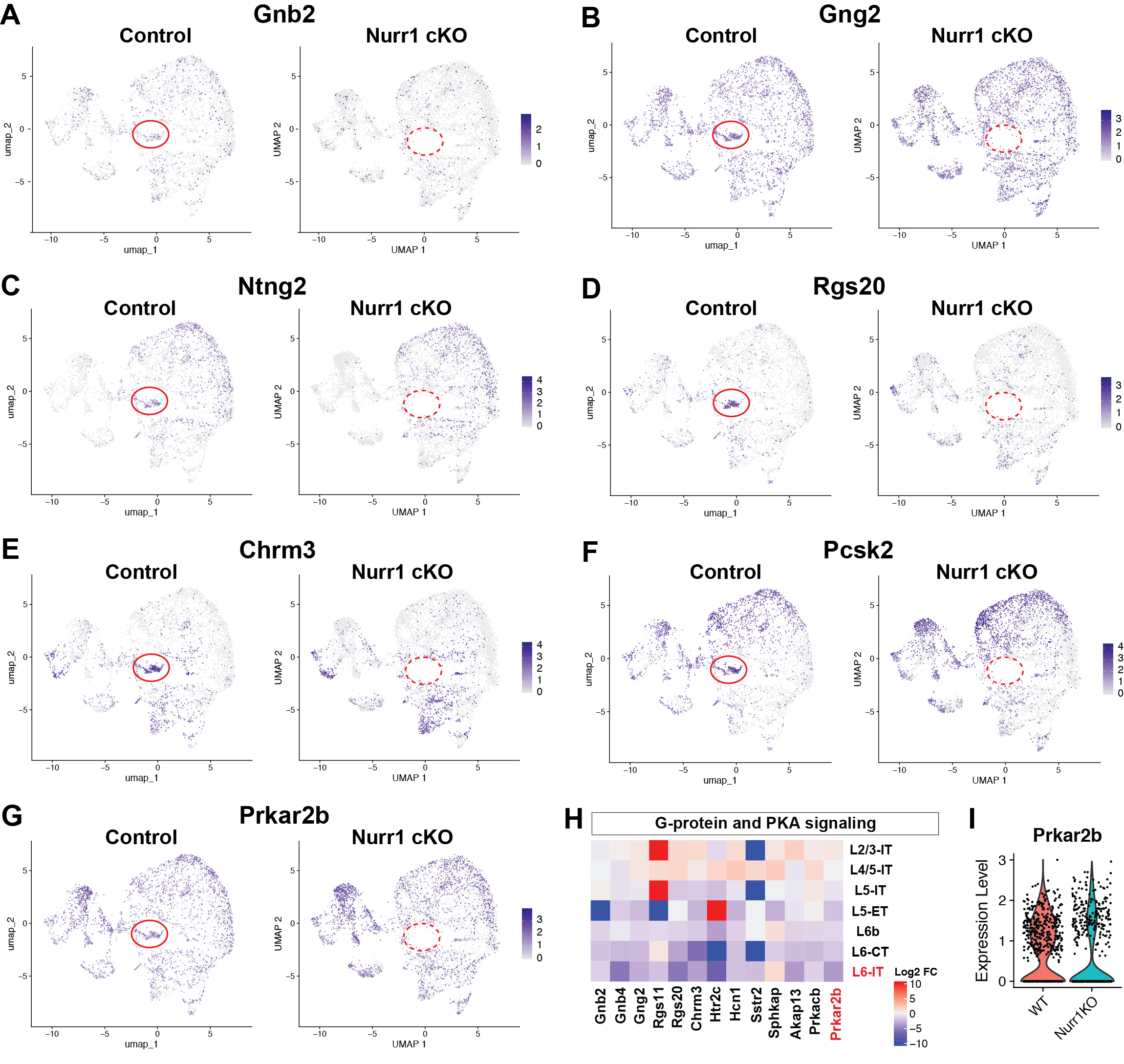

Supplement: Supplementary file 11 — Supporting File 11: advs73465‐sup‐0011‐Figure S10.png. [file ADVS-13-e08999-s007.png]

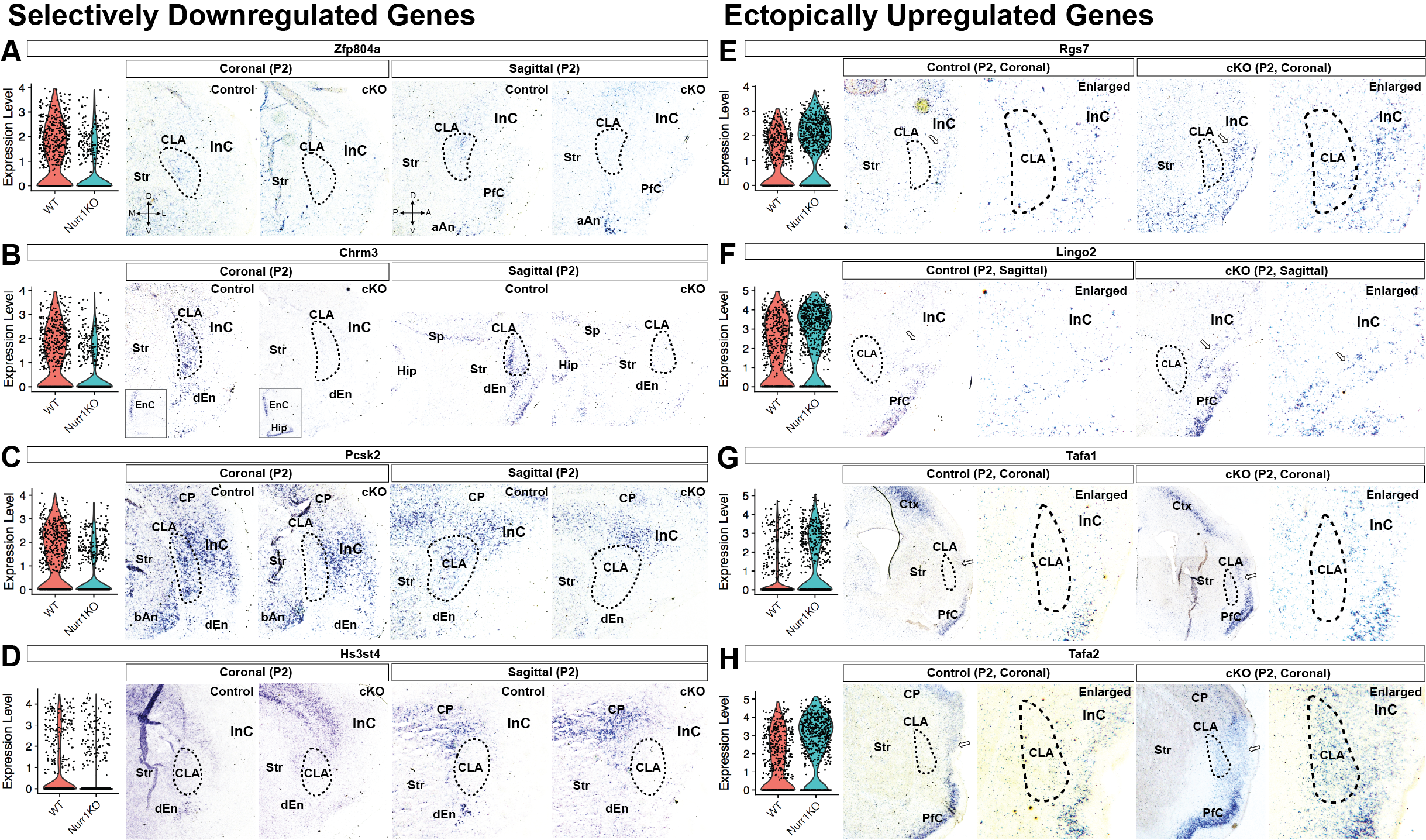

Supplement: Supplementary file 12 — Supporting File 12: advs73465‐sup‐0012‐Figure S11.png. [file ADVS-13-e08999-s012.png]

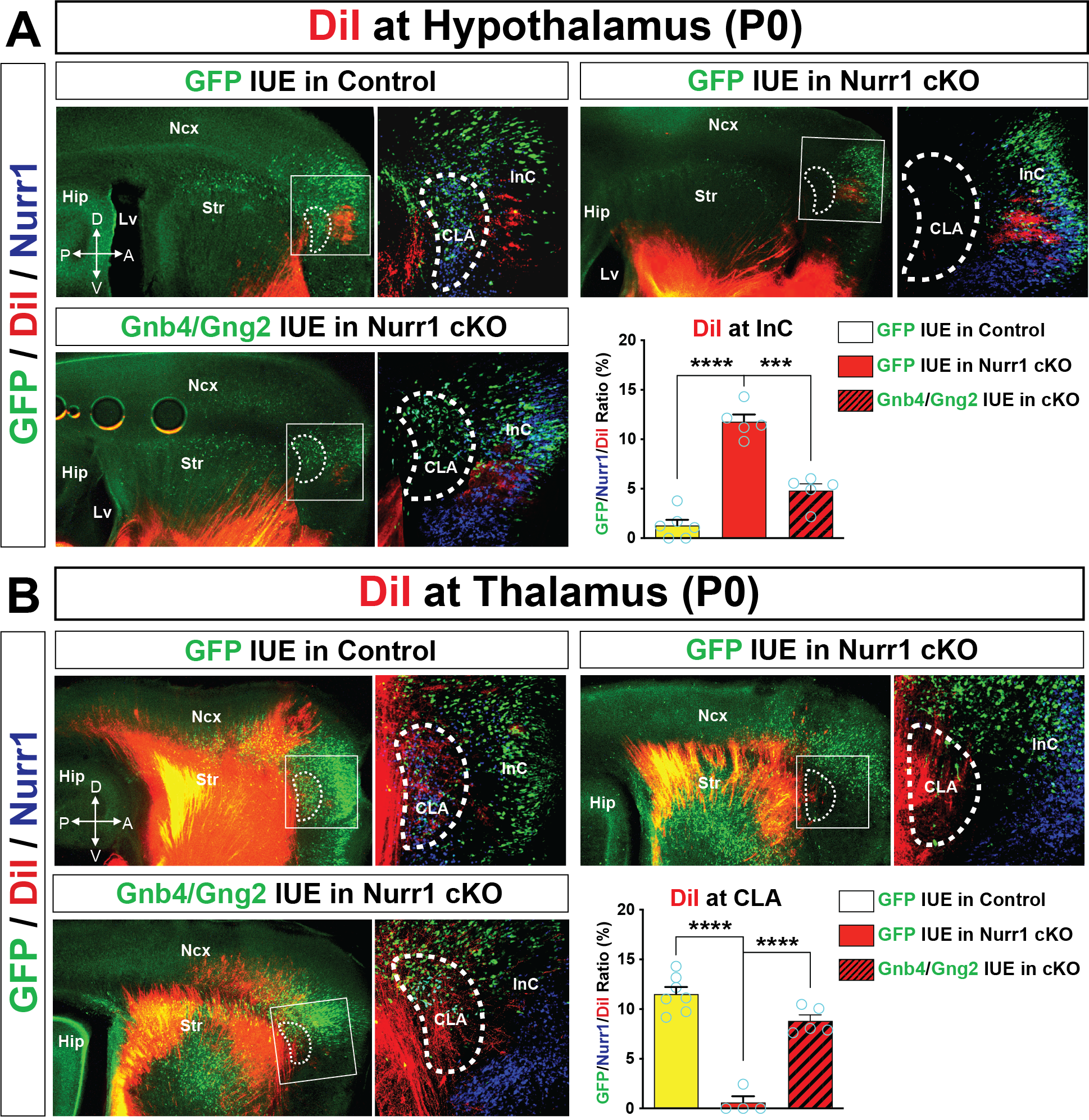

Supplement: Supplementary file 13 — Supporting File 13: advs73465‐sup‐0013‐Figure S12.png. [file ADVS-13-e08999-s004.png]

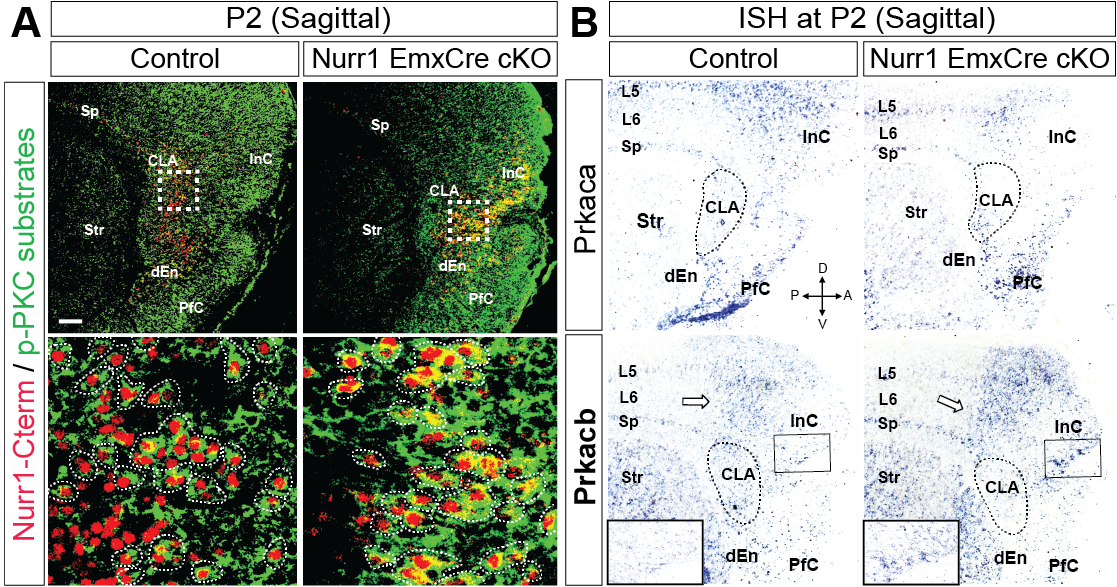

Supplement: Supplementary file 14 — Supporting File 14: advs73465‐sup‐0014‐Figure S13.png. [file ADVS-13-e08999-s016.png]

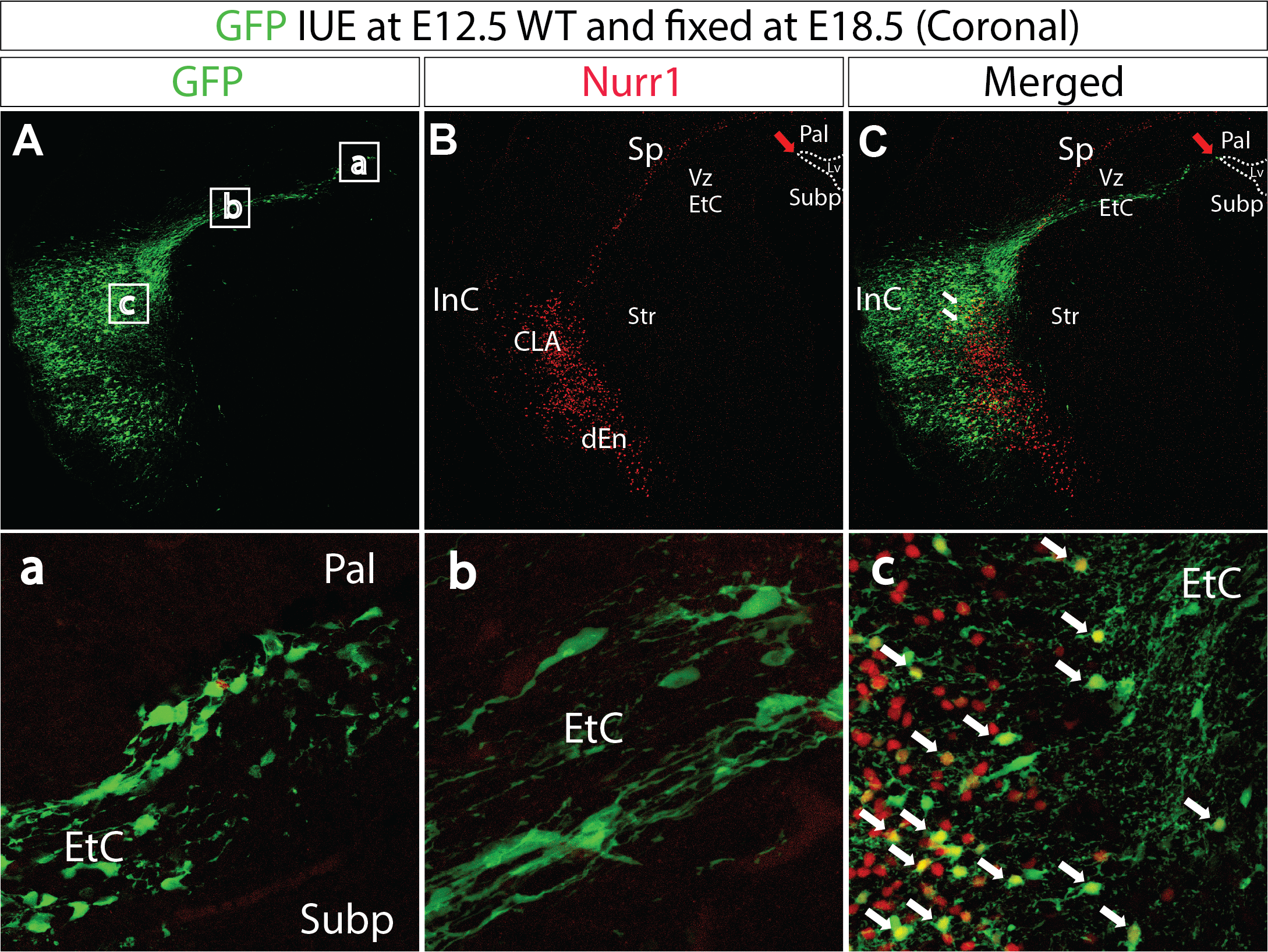

Supplement: Supplementary file 15 — Supporting File 15: advs73465‐sup‐0015‐Figure S14.png. [file ADVS-13-e08999-s015.png]
