# Supplementary material for: Nurr1 Orchestrates Claustrum Development and Functionality
Source: Adv Sci (Weinh). 2025 Dec 22;13(13):e08999. doi: 10.1002/advs.202508999 (PMC12955989; doi:10.1002/advs.202508999)
Supplement: Supplementary file 16 — Supporting File 16: advs73465‐sup‐0016‐Table S1.docx. [file ADVS-13-e08999-s018.docx]

**Table S1 Nucleus numbers and proportions in Nurr1 lineage cells of transcriptomic analysis_­­_**

| Genotype | L2/3-IT | L4/5-IT | L5-IT | L5-ET | L6-IT | L6-CT | L6b | Total |
| --- | --- | --- | --- | --- | --- | --- | --- | --- |
| Control | 41  (7.59%) | 76  (14.07%) | 28  (5.19%) | 11  (2.04%) | 258  (47.78%) | 69  (12.78%) | 57  (10.56%) | 540 |
| Nurr1 cKO | 79 (12.40%) | 232  (36.42%) | 47  (7.38%) | 6  (0.94%) | 151  (23.70%) | 43  (6.75%) | 79 (12.40%) | 637 |
| Change | 63.37%  increase | 158.85%  increase | 42.20%  increase |  | 50.40%  decrease | 47.18%  decrease | 16.86%  increase |  |

Note: 1 and 2 cells in the L5-NP clusters of control and Nurr1 deficient brains are excluded from counting, respectively.
