# Supplementary material for: Nurr1 Orchestrates Claustrum Development and Functionality
Source: Adv Sci (Weinh). 2025 Dec 22;13(13):e08999. doi: 10.1002/advs.202508999 (PMC12955989; doi:10.1002/advs.202508999)
Supplement: Supplementary file 17 — Supporting File 17: advs73465‐sup‐0017‐Table S2.docx. [file ADVS-13-e08999-s003.docx]

**Table S2 Oligos and Primers**

| **Name:** | **Usage:** | **Sequence: 5’ – 3’** |
| --- | --- | --- |
| Nurr1-Nterm-Fw | Nurr1 ISH for 5’end | TCCTCGCCTCAAGGAGCCAGCCCCG |
| Nurr1-Nterm-Rv |  | AAGTGCGAACACCGTAGTGCTGACA |
| Nurr1-Cterm-Fw | Nurr1 ISH for 3’end | GTTAAAGAAGTGGTTCGCACGGACA |
| Nurr1-Cterm-Rv |  | CTTAGAAAGGTAAGGTGTCCAGGAAA |
| Cux2-Fw | Cux2 ISH | GATGGAGACAGCCAGCCCCAGG |
| Cux2-Rv |  | TTCAGAATTCCCACTCCAGGAC |
| Cdh13-Fw | Cdh13 ISH | TCGCTACTTATCAACTGTATGTGGA |
| Cdh13-Rv |  | TGGGTCCTTGTAGATAGAGTACCTG |
| Fezf2-Fw | Fezf2 ISH | TCATGTGATGTCAGCTGAATGTAAA |
| Fezf2-Rv |  | TGGAGTCCAGGTAGTTGAAGTAGTA |
| Nfib-Fw | Nfib ISH | TCAATGTATCAGAGCTTGTGAGAGT |
| Nfib-Rv |  | AAGGGAATTAGTGACTGTAAGTGCT |
| NtnG2-Fw | NetrinG2 ISH | GAAGGATTATGTCAAGGTCAAAGTG |
| NtnG2-Rv |  | CGATATTGGAGATGGCATAGAAGTA |
| Gnb4-Fw | Gnb4 ISH | ATATACAACCTAAAGACCCGAGAGG |
| Gnb4-Rv |  | GAGAACAGAAAATATGGCACTCAAT |
| Gng2-Fw | Gng2 ISH | GGGGAAGCTGCTCTCTAACCAAGCC |
| Gng2-Rv |  | GACAGCTTATCAGAGGGTATTTGAA |
| Rgs20-Fw | Rgs20 ISH | AGTAGGAACCGCTCTGACTAGTGTA |
| Rgs20-Rv |  | CTGTTTTCTCAGCTAAGGACGTAAG |
| CTGF-Fw | CTGF (Ccn2) ISH | AGTTACCAATGACAATACCTTCTGC |
| CTGF-Rv |  | CCACGGTAGTTAAAAACACAGATTT |
| S100a10-Fw | S100a10 ISH | ATGATGCTTACGTTTCACAGGTTTG |
| S100a10-Rv |  | CCATTGGATTAAGTTTTCTCTCTCA |
| Zbtb20-Fw | Zbtb20 ISH | GACACATTCACTGACAAACTCTCAC |
| Zbtb20-Rv |  | AGTCATAGTCATCTTCCATTTCCTG |
| Prkaca-Fw | Prkaca ISH | GAAGATCTTAGACAAGCAGAAGGTG |
| Prkaca-Rv |  | ATAGTCGTCAAAGTTACTCGTGTCC |
| Prkacb-Fw | Prkacb ISH | TGGATTGCTATTTATCAGAGAAAGG |
| Prkacb-Rv |  | CTTACTATCTCACGGAGTGAAGAGC |
| Nurr1-Fw-XhoI | Generation of Nurr1 expression construct | TTTTTCTCGAGAGCCATGCCTTGTGTTCAGGCGCAGTAT |
| Nurr1-Rv-NotI |  | TTTTTGCGGCCGCTTAGAAAGGTAAGGTGTCCAGGAA |
| Gnb4-Fw-XhoI | Generation of Gnb4 expression construct | TTTTTCTCGAGGATGAGCGAGCTGGAGCAGCTGAGG |
| Gnb4-Rv-NotI |  | TTTTTGCGGCCGCTCAATTCCAGATTCTAAGAAAACT |
| Gng2-Fw-XhoI | Generation of Gng2 expression construct | TTTTTCTCGAGACCATGGCCAGCAACAACACCGCCA |
| Gng2-Rv-NotI |  | TTTTTGCGGCCGCTTAAAGGATGGCGCAGAAGAACT |
| Tafa1-Fw | Tafa1 ISH | CCTAAAATCCATTATCCCTGTCTTT |
| Tafa1-Rv |  | TGCTGTAACTAAGAAATGAGTGCTG |
| Tafa2-Fw | Tafa2 ISH | TAACCCATTAACCACTCCTAAATCA |
| Tafa2-Rv |  | ACAACATGTAGGCTTTCAGAGTTTC |
| Zfp804a-Fw | Zfp804a ISH | AAGAAACAATGAACACAACAGTGAA |
| Zfp804a-Rv |  | GTTTGCTCTGAGTTCTTCTCGATAC |
| Chrm3-Fw | Chrm3 ISH | CCTTGTAGAAAAGGGGTTTATCAAT |
| Chrm3-Rv |  | ATTTGTATGAAACTTGAACTGCACA |
| Hs3st4-Fw | Hs3st4 ISH | CTCTTCATGTGCACCCTGTC |
| Hs3st4-Rv |  | GGGTCAAGAAAGGAGGGACA |
| Pcsk2-Fw | Pcsk2 ISH | GTGACTCGACCTTTATTTCTGTCAT |
| Pcsk2-Rv |  | TCATAACAGGGAACTCTAAAACCAC |
| Lingo2-Fw | Lingo2 ISH | GTATACCTGACCCACCTTAACCTCT |
| Lingo2-Rv |  | TTTTAAGGTCCAGGGAGAAAGTATT |
| Rgs7-Fw | Rgs7 ISH | AGATTGATCATCCTTGTATCTGAGC |
| Rgs7-Rv |  | TCTCTTCTTGTCCACTTTAGCTTGT |
| Nurr1-GT-Fw | Nurr1 floxed genotyping | GCTGGAGCCAGAGTTGGAAG |
| Nurr1-GT-Rv |  | ATTCCTTGGAGACCTTCTCT |
| EmxCre-PrimerA | EmxCre genotyping | TCGATGCAACGAGTGATGAG |
| EmxCre-PrimerB |  | TTCGGCTATACGTAACAGGG |
| EmxCre-PrimerC |  | AAGGTGTGGTTCCAGAATCG |
| EmxCre-PrimerD |  | CTCTCCACCAGAAGGCTGAG |
| NexCre-PrimerA | NexCre genotyping | CCGCATAACCAGTGAAACAG |
| NexCre-PrimerB |  | GAGTCCTGGAATCAGTCTTTTTC |
| NexCre-PrimerC |  | AGAATGTGGAGTAGGGTGAC |

**ISH,** *in situ* hybridization.
