# Supplementary material for: Nurr1 Orchestrates Claustrum Development and Functionality
Source: Adv Sci (Weinh). 2025 Dec 22;13(13):e08999. doi: 10.1002/advs.202508999 (PMC12955989; doi:10.1002/advs.202508999)
Supplement: Supplementary file 18 — Supporting File 18: advs73465‐sup‐0018‐Table S3.docx. [file ADVS-13-e08999-s009.docx]

**Table S3 Primary antibodies**

| Primary Antibody | Species | Company | Cat # | Dilution | Usage | RRID |
| --- | --- | --- | --- | --- | --- | --- |
| Nurr1 N terminal | Mouse | Santa Cruz | sc-376984 | 1:300 | IF | AB_2893391 |
| Nurr1 C terminal | Goat | R&D systems | AF2156 | 1:500 | IF | AB_2153894 |
| Nestin | Mouse | Merck | MAB353 | 1:800 | IF | AB_94911 |
| Zbtb20 | Rabbit | SIGMA | HPA016815 | 1:300 | IF | AB_1858947 |
| GFP | Rabbit | ThermoFisher | A-11122 | 1:500 | IF | AB_2307355 |
| GFP | Chicken | Abcam | Ab13970 | 1:1000 | IF | AB_300798 |
| Tle4 | Mouse | Santa Cruz | sc-365406 | 1:500 | IF | AB_10841582 |
| Darpp32 | Rabbit | Abcam | ab40801 | 1:500 | IF | AB_731843 |
| Cleaved Caspase 3 | Rabbit | Cell Signaling | 9579 | 1:500 | IF | AB_10897512 |
| Cytochrome P450 26b1 (Cyp26b1) | Mouse | Merck | MABS497 | 1: 500 | IF | unidentified |
| Neurod1 | Rabbit | Proteintech | 12081-1-AP | 1:1500 | IF | AB_2877823 |
| Rorβ | Mouse | R&D systems | PP-N7927-00 | 1:500 | IF | AB_1964364 |
| Phospho-(Ser/Thr) PKA substrates | Rabbit | Cell Signaling | 9621 | 1:800 | IF | AB_330304 |
| Phospho-PKC substrates | Rabbit | Cell Signaling | 6967 | 1:800 | IF | AB_10949977 |

**IF,** immunofluorescence.
